# NDP52 acts as a redox sensor in PINK1/Parkin‐mediated mitophagy

**DOI:** 10.15252/embj.2022111372

**Published:** 2022-12-14

**Authors:** Tetsushi Kataura, Elsje G Otten, Yoana Rabanal‐Ruiz, Elias Adriaenssens, Francesca Urselli, Filippo Scialo, Lanyu Fan, Graham R Smith, William M Dawson, Xingxiang Chen, Wyatt W Yue, Agnieszka K Bronowska, Bernadette Carroll, Sascha Martens, Michael Lazarou, Viktor I Korolchuk

**Affiliations:** ^1^ Faculty of Medical Sciences, Biosciences Institute Newcastle University Newcastle Upon Tyne UK; ^2^ Max Perutz Labs, Vienna BioCenter (VBC) University of Vienna Vienna Austria; ^3^ Chemistry – School of Natural and Environmental Sciences Newcastle University Newcastle Upon Tyne UK; ^4^ Bioinformatics Support Unit (BSU), Faculty of Medical Sciences Newcastle University Newcastle Upon Tyne UK; ^5^ School of Chemistry University of Bristol Bristol UK; ^6^ College of Veterinary Medicine Nanjing Agricultural University Nanjing China; ^7^ School of Biochemistry University of Bristol Bristol UK; ^8^ Department of Biochemistry and Molecular Biology, Biomedicine Discovery Institute Monash University Melbourne VIC Australia; ^9^ Walter and Eliza Hall Institute of Medical Research Parkville VIC Australia; ^10^ Present address: Amphista Therapeutics Cambridge UK; ^11^ Present address: Department of Medical Sciences, Faculty of Medicine University of Castilla‐la Mancha Ciudad Real Spain; ^12^ Present address: Università Degli Studi della Campania “Luigi Vanvitelli” Caserta Italy

**Keywords:** autophagy, mitophagy, NDP52, p62, redox, Autophagy & Cell Death

## Abstract

Mitophagy, the elimination of mitochondria via the autophagy‐lysosome pathway, is essential for the maintenance of cellular homeostasis. The best characterised mitophagy pathway is mediated by stabilisation of the protein kinase PINK1 and recruitment of the ubiquitin ligase Parkin to damaged mitochondria. Ubiquitinated mitochondrial surface proteins are recognised by autophagy receptors including NDP52 which initiate the formation of an autophagic vesicle around the mitochondria. Damaged mitochondria also generate reactive oxygen species (ROS) which have been proposed to act as a signal for mitophagy, however the mechanism of ROS sensing is unknown. Here we found that oxidation of NDP52 is essential for the efficient PINK1/Parkin‐dependent mitophagy. We identified redox‐sensitive cysteine residues involved in disulphide bond formation and oligomerisation of NDP52 on damaged mitochondria. Oligomerisation of NDP52 facilitates the recruitment of autophagy machinery for rapid mitochondrial degradation. We propose that redox sensing by NDP52 allows mitophagy to function as a mechanism of oxidative stress response.

## Graphical abstract

Human autophagy receptor NDP52 senses reactive oxygen species to mark damaged mitochondria for removal.

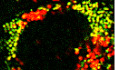

## Introduction

Macroautophagy (hereafter referred to as autophagy) is a bulk cellular recycling process induced upon starvation to degrade cytosolic macromolecules, whilst also being involved in the selective degradation of damaged and potentially damaging components such as protein aggregates and dysfunctional organelles (Dikic & Elazar, 2018). Targeting of autophagy cargo to the nascent autophagosome (called phagophore) is dependent on a family of selective autophagy receptors (SARs; Conway *et al*, 2020). These proteins are typically characterised by their ability to bind both the ubiquitinated cargo and the autophagy proteins. This tethering and scaffolding function of SARs allows phagophore expansion and closure around the cargo to form an autophagosome. Autophagosomes subsequently fuse with lysosomes where the cargo is degraded, and the contents are recycled. The prototypic member of the SAR family p62/SQSTM1 has the ability to assemble into oligomers which are maintained by non‐covalent interactions of its N‐terminal PB1 domain (Johansen & Sachse, 2015). This oligomerisation step is essential for the formation of intracellular protein aggregates and their selective autophagic degradation (aggrephagy; Itakura & Mizushima, 2011). The mechanistic explanation for the requirement of higher order receptor oligomers is that this process increases the avidity of protein–protein interactions and enhances the recruitment of autophagy initiation proteins (Wurzer *et al*, 2015). In addition to the non‐covalent mode of oligomerisation, we and others have demonstrated that the self‐assembly of p62 can also be triggered in response to ROS. In this process, oxidation of specific cysteine residues in p62 promotes the formation of disulphide‐linked conjugates (DLC). This redox sensing was found to be essential for the ability of p62 to drive autophagy, and particularly aggrephagy, resulting in the increased stress resistance in mammalian cells and *Drosophila* (Cha‐Molstad *et al*, 2017; Carroll *et al*, 2018).

Whilst the origin of ROS triggering p62 oxidation and mediated by it aggrephagy remains poorly understood, the major source of cellular ROS is mitochondria (Sedlackova *et al*, 2020). In particular, a damage to the components of the mitochondrial electron transport chain results in an elevated generation of ROS during respiration. The oxidative stress triggered by mitochondrial damage can cause cellular dysfunction and has been associated with a range of human pathologies. Most notably, these include age‐related neurodegenerative disorders such as Parkinson's disease characterised by the loss of dopaminergic neurons (Hou *et al*, 2020). Therefore, removal of damaged mitochondria is essential for the maintenance of normal cellular physiology, and the selective autophagic clearance of mitochondria (mitophagy) is increasingly recognised as the key quality control mechanism. PTEN‐induced Kinase (PINK1)/Parkin‐mediated mitophagy is arguably the most well‐studied selective autophagy pathway to date (Pickles *et al*, 2018). PINK1 is a protein kinase which accumulates at the outer membrane of damaged mitochondria with reduced membrane potential. Stabilisation of PINK1 allows it to phosphorylate several downstream targets including the E3 ubiquitin ligase Parkin and ubiquitin itself. Ubiquitination of mitochondrial surface proteins by Parkin in turn initiates a cascade of molecular events leading to the engulfment and sequestration of the damaged mitochondrion by a newly formed autophagosome (also termed mitophagosome). Similar to the evidence of mitochondrial dysfunction as the driver of neurodegeneration, mutations in PINK1 and Parkin have been associated with familial forms of Parkinson's disease indicating that mitochondrial damage‐induced mitophagy is important for the function and long‐term survival of neurons (Hou *et al*, 2020).

Damage‐induced mitophagy involves binding of SARs to the ubiquitinated mitochondria. OPTN, NDP52, TAX1BP1, NBR1, and p62 have all been shown to be recruited to damaged mitochondria; however, only OPTN, NDP52, and, to a lesser extent, TAX1BP1 were found to be required for mitophagy initiation (Lazarou *et al*, 2015). Mitophagy receptors trigger a chain reaction of protein–protein interactions primarily aiming to attract, in a rapid and efficient manner, the autophagy initiation machinery including the microtubule‐associated protein 1 light chain 3 (LC3), FIP200, and autophagy‐related 13 (ATG13) and ATG16 (Padman *et al*, 2019; Vargas *et al*, 2019). Whilst the ROS produced by damaged mitochondria may provide a mechanism for disulphide‐mediated oligomerisation of mitophagy receptors, whether oligomerisation of receptors occurs during mitophagy and plays a functional role in this process remains unknown (Sedlackova *et al*, 2020).

Here, we show that one of the key mitophagy receptor proteins NDP52 is redox‐regulated and its ability to sense ROS generated by damaged mitochondria is required for the efficiency of PINK1/Parkin‐mediated mitophagy. Upon mitochondrial damage, NDP52 becomes recruited to the surface of mitochondria where ROS triggers its oligomerisation mediated by disulphide bonds. The oligomeric species of NDP52 facilitate the mitophagy kinetics by recruitment of the autophagy initiation machinery components and thereby promote the formation of an autophagosome engulfing the damaged mitochondrion and subsequent lysosomal degradation.

## Results

### NDP52 forms DLC in response to oxidative stress or mitochondrial damage

**Figure 1:**
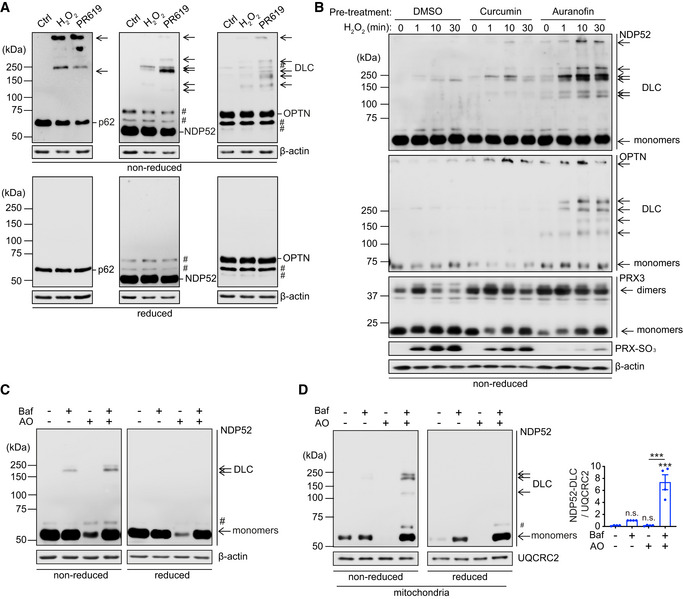
NDP52 forms DLC in response to oxidative stress or mitochondrial damage AHeLa cells were treated with H_2_O_2_ (5 mM, 1 min) or PR‐619 (10 μM, 10 min) and analysed by immunoblotting for endogenous p62, NDP52 and OPTN in either reducing (2.5% β‐mE) or non‐reducing conditions.BNDP52 and OPTN are reducible by the thioredoxin antioxidant system. HeLa cells were pre‐treated with TrxR inhibitors, curcumin (50 μM) or auranofin (5 μM) for 30 min, then treated with H_2_O_2_ (500 μM) at different time points as indicated. Immunoblotting was performed to detect NDP52, OPTN and PRX‐3 (dimer formation reflecting oxidation of the protein) in non‐reduced conditions and PRX‐SO3 (marker of oxidative stress (Carroll *et al*, 2018)) and actin in reduced conditions.C, DImmunoblotting for NDP52 in either reducing or non‐reducing conditions of whole cell samples (C) and mitochondrial fractions (D) from HeLa cells treated with the combination of 4 μM antimycin A1 and 10 μM oligomycin (AO) in the presence or absence of 400 nM bafilomycin A1 (Baf) for 2 h. NDP52 DLC in mitochondrial fraction was quantified (D). Data are mean ± s.e.m (D). *P* values were calculated by one‐way ANOVA followed by Sidak test on four independent experiments (D). ***, *P* < 0.001; ns (non‐significant). Data information: #, non‐specific band or a post‐translational modification. Source data are available online for this figure.

To test if the mitophagy receptors OPTN and NDP52 can respond to oxidative stress by forming disulphide‐linked oligomers analogous to p62, we exposed HeLa cells to hydrogen peroxide and PR619, a strong redox cycler (Carroll *et al*, 2018). Mitophagy receptors readily formed DLC characterised by their sensitivity to a reducing agent (Fig 1A). Similar to p62, the OPTN and NDP52 DLC were accumulating in the presence of thioredoxin reductase inhibitors curcumin and auranofin indicating that saturation of thioredoxin antioxidant buffering capacity (reflected by the oxidation of and PRX‐3 and formation of PRX‐SO_3_) leads to their stabilisation (Fig 1B; Carroll *et al*, 2018). Redox sensitivity was found to be specific to SARs as well as two other autophagy proteins ATG5 and ATG7 (Frudd *et al*, 2018), whilst several other proteins tested in the same conditions (TFEB, UQCRC2, S6 and GAPDH) did not form DLC (Appendix Fig S1).

**Figure EV1:**
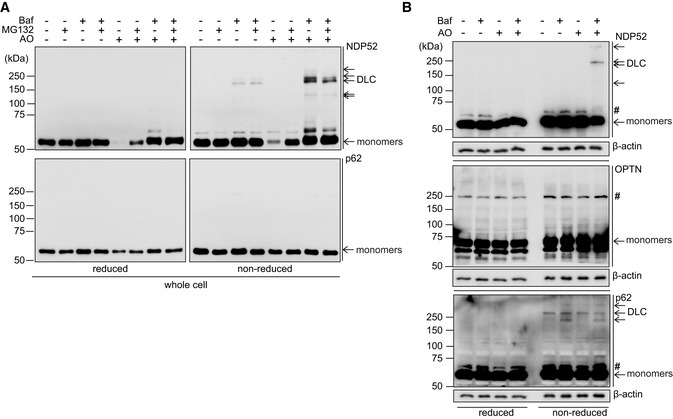
NDP52 but not p62 nor OPTN forms DLC upon mitochondrial damage A, BHeLa cells were treated with 4 μM/10 μM AO in the presence or absence of 400 nM Baf (A, B) and 10 μM MG132 (A) for 3 h and analysed by immunoblotting for endogenous NDP52, p62 and OPTN in either reducing (2.5% β‐mE) or non‐reducing conditions. #, non‐specific band or a post‐translational modification. Source data are available online for this figure.

Next, we tested whether blocking mitochondrial Complex III and V with antimycin and oligomycin (AO), a combination commonly used to induce depolarisation of mitochondria and induce PINK1/Parkin‐dependent mitophagy (Lazarou *et al*, 2015), is associated with receptor DLC formation. Consistent with previous reports, AO treatment alone resulted in the depletion of NDP52 suggesting its rapid turnover upon mitochondrial damage (Fig 1C and D; Lazarou *et al*, 2015). Interestingly, NDP52 DLC were detectable in whole cell and mitochondria‐enriched fractions when autophagy was blocked by a lysosomotropic agent bafilomycin A1 and further increased when cells were also treated with AO. Bafilomycin A1 also stabilised monomeric species of NDP52 in AO‐treated cells (Fig 1C and D). In contrast, blocking the ubiquitin proteasome system, which was previously shown to contribute to degradation of SARs upon mitochondrial damage, with MG132 partially stabilised monomeric NDP52 but not DLC (Fig EV1A; Lazarou *et al*, 2015). Therefore, in response to mitochondrial damage NDP52 forms DLC which are degraded predominantly via the autophagy‐lysosome pathway. In contrast to NDP52, no DLC was detected for OPTN in conditions of our AO/bafilomycin A1 treatment protocol, whilst p62 DLC stabilised by bafilomycin A1 was not increased upon AO treatment (Fig EV1B). We conclude that NDP52 is a specific redox sensor in response to mitochondrial damage that triggers the formation and autophagic degradation of NDP52 DLC.

**Figure 2:**
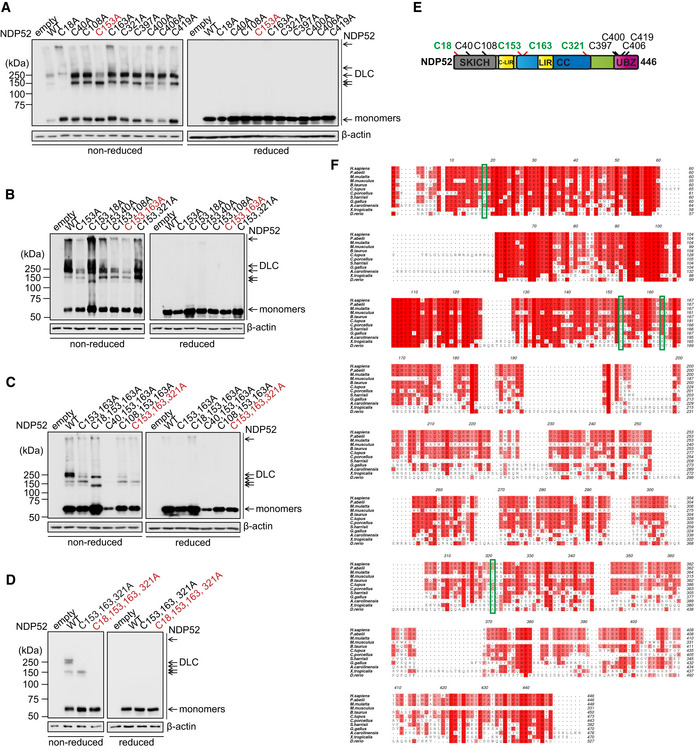
Cysteine residues 18, 153, 163 and 321 of NDP52 are required to form DLC A–DSingle (A), double (B), triple (C), and quadruple (D) cysteine mutant NDP52 plasmids were generated and transfected into HeLa PentaKO cells. Cells were treated with 20 μM PR‐619 for 10 min and analysed by immunoblotting for NDP52 in either reducing or non‐reducing conditions. The constructs highlighted in red were subjected to the next round of Cys‐Ala screen or further analyses.ESchematic diagram of the NDP52 domain organisation showing the location of the redox sensitive cysteines.FMultiple protein sequence alignment of NDP52 sequences in different species. Increasing conservation is shown by light–dark red; cysteine residues 18, 153, 163 and 321 are indicated by green boxes, and these are conserved in primates. Source data are available online for this figure.

### The model of NDP52 oligomerisation mediated by Cys residues 18, 153, 163 and 321

Recombinant NDP52 was found to readily form DLC *in vitro* in response to hydrogen peroxide but not AO (Appendix Fig S2A). This indicated that (i) the protein is capable to form homo‐oligomers via disulphide bonds in the presence of ROS and (ii) AO does not have a direct pro‐oxidant effect implicating its effect in cells via mitochondrial damage which potentially triggers oxidative stress. To identify the cysteine residues involved in the DLC formation we performed four rounds of Cys‐Ala scanning where stepwise mutations of C153, C163, C321 and C18 resulted in the complete loss of DLC formation by NDP52 (Fig 2A–F). Three of the Cys residues (C153, C163, C321) are located in the coiled coil (CC) domain of NDP52 previously shown to mediate dimerisation of the protein (Kim *et al*, 2013; Fig 2E). Interestingly, those Cys residues were not present in lower mammals such as mouse (Fig 2F).

**Figure 3:**
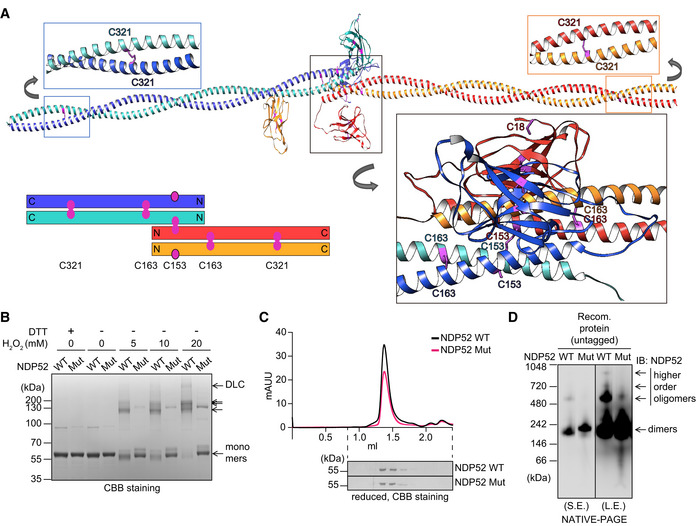
Modelling of the NDP52 tetramer and the properties of NDP52 C18, 153, 163, 321S ACalculated conformation of the antiparallel NDP52 tetramer and residue interactions after 100 ns of MD simulation. The schematic summarises the predicted orientation of Cys residues within CC domains of the NDP52 tetramer.BRecombinant proteins of NDP52 WT and C18, 153, 163, 321S mutant (Mut) were exposed to the indicated concentrations of H_2_O_2_ for 5 min and subjected to SDS‐PAGE and CBB gel staining.CAnalytical size‐exclusion chromatography (SEC) of the NDP52 WT and Mut proteins.DRecombinant NDP52 WT and Mut proteins (5 ng) were subjected to NATIVE‐PAGE analysis. S.E., short exposure; L.E., long exposure. Source data are available online for this figure.

**Figure EV2:**
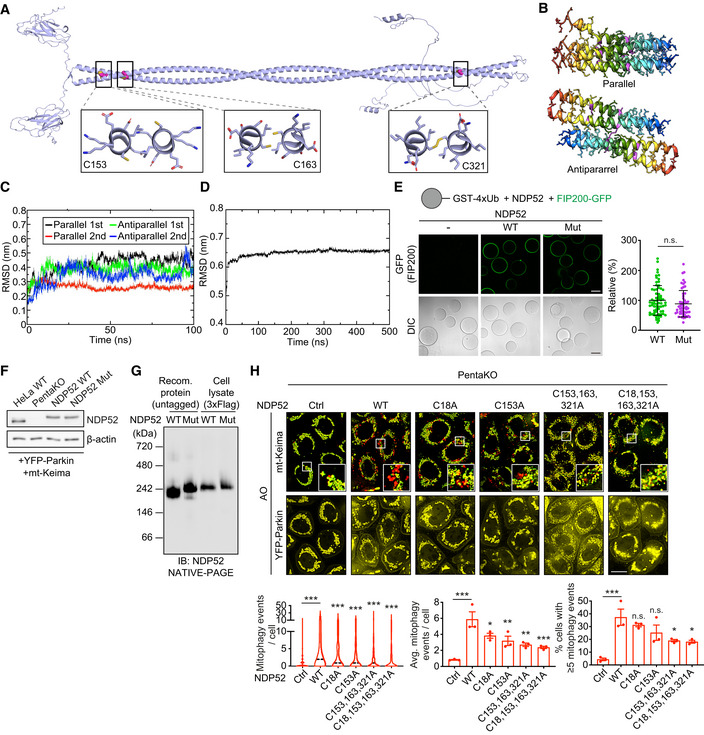
Modelling of NDP52 tetramers and the property of NDP52 cysteine mutants AStructural model of NDP52 dimer generated by AlphaFold2. Cys163 and Cys321 residues are both part of the coiled‐coil interface and are orientated towards each other (within a distance of ~2.8 Å and ~1.3 Å, respectively). Cys153 is not part of the hydrophobic core and is orientated towards opposing sides of the homodimer.BStructural models of a parallel or an antiparallel tetramer (dimer of dimers) of the NDP52 protein.CCalculated root‐mean‐square deviation (RMSD) from atomistic molecular dynamics (MD) simulations for 100 ns of the NDP52 parallel and antiparallel tetramers fitted between the SKICH and CC domains.DCalculated RMSD from coarse‐grain MD simulations of the NDP52 antiparallel tetramer interacted between SKICH domains for 500 ns.EConfocal images showing the recruitment of FIP200‐GFP to glutathione Sepharose beads coated with GST‐4xUbiquitin (Ub) and incubated with untagged wild type or mutant NDP52.FImmunoblotting to analyse the expression level of NDP52 in HeLa WT, PentaKO, PentaKO + NDP52 WT and PentaKO + NDP52 Mut cells stably expressing YFP‐Parkin and mt‐mKeima.GRecombinant NDP52 WT and NDP52 Mut (2 ng) proteins, and cell lysates (30 μg) of HeLa PentaKO cells stably expressing NDP52 WT or NDP52 Mut, were subjected to NATIVE‐PAGE followed by immunoblotting analyses.HFluorescence images and quantification of mitophagy in HeLa PentaKO cells stably expressing YFP‐Parkin and mt‐mKeima, transiently transfected with the indicated NDP52 constructs and treated with AO for 2 h. Data information: Data are mean ± s.e.m. or displayed as cell popular violin plots (D, G). *P* values were calculated by unpaired two‐tailed Student's *t*‐test (D) or one‐way ANOVA followed by Sidak test (G) on three independent experiments. *, *P* < 0.05; **, *P* < 0.01; ***, *P* < 0.001; ns (non‐significant). Scale bars: 50 μm (D); 20 μm (G). Source data are available online for this figure.

Atomistic molecular modelling of CC dimer indicated that C163 and C321 form C163–C163 and C321–C321 disulphide bonds, potentially stabilising the dimer (Figs 3A and EV2A). On the other hand, C153 is predicted to be positioned away from the dimer interface and can be available for further crosslinking (Figs 3A and EV2A). Modelling studies with multiscale molecular dynamics simulations indicated that C153–C153 disulphide bonds between the two dimers can generate a stable anti‐parallel tetramer rather than all‐parallel tetramer in terms of its interaction energies (Figs 3A and EV2B–D). The presence of two outward facing C153 residues per CC tetramer implies that C153 can also mediate the formation of higher order NDP52 oligomers via disulphide linking (Fig 3A). Disulphide bonds involving C18 within the SKIP carboxyl homology (SKICH) domain can further contribute to NDP52 oligomerisation (Figs 2E and 3A).

We next investigated the impact of the Cys mutations on the properties of NDP52. In order to minimise alterations to the protein structure, we generated NDP52 constructs where four Cys residues required for DLC formation were replaced with Ser. Analysis of the recombinant C18, 153, 163, 321S NDP52 mutant confirmed the loss of its ability to form DLC in response to hydrogen peroxide *in vitro* (Fig 3B and Appendix Fig S2B). At the same time, in reduced conditions, Cys mutant showed similar to wild‐type protein stability and behaviour on analytical size exclusion chromatography as well as the affinity towards FIP200 and ubiquitin‐coated beads, indicating that the Cys mutant retains the structure and function at basal state (Turco *et al*, 2019; Figs 3C and EV2E). Both NDP52 proteins were detected at approximately 200 kDa by blue native PAGE (BN‐PAGE) consistent with the rod‐like structure of the CC dimer (Fig 3A and D). On the other hand, the formation of higher order oligomeric species was strongly reduced by Cys mutations, which supports our model that Cys residues mediate the oligomerisation of NDP52 (Fig 3A and D).

### ROS‐dependent oxidation of NDP52 is required for the efficient activation of mitophagy

To investigate the impact of NDP52 DLC on mitophagy, we introduced wild type and C18, 153, 163, 321S mutant NDP52 into HeLa cell line with CRISPR/Cas9‐engineered knockout of 5 SARs (PentaKO) which was previously shown to be deficient in PINK1/Parkin‐dependent mitophagy (Lazarou *et al*, 2015). All four cell lines were additionally transduced with YFP‐Parkin and mt‐mKeima to allow mitophagy studies, and the expression of both NDP52 transgenes was comparable with the endogenous protein levels in CRISPR control HeLa cell line (Fig EV2F). Both proteins also showed similar to recombinant NDP52 behaviour on BN‐PAGE, confirming that the mutant retains the dimer structure in the cells (Fig EV2G).

**Figure 4:**
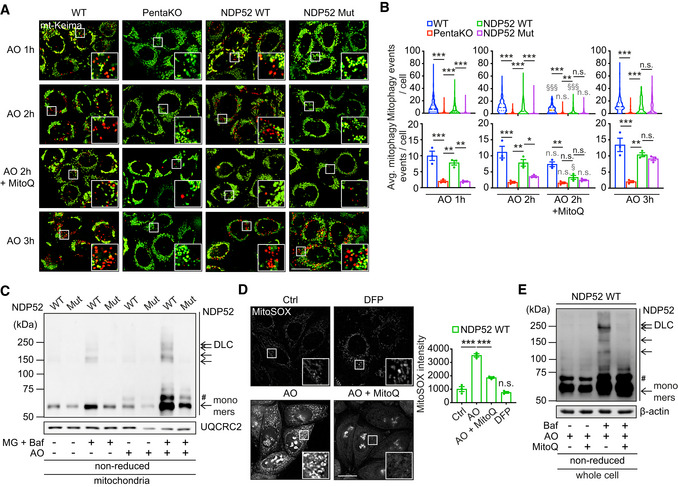
Mitochondrial ROS‐dependent oxidation of NDP52 is required for the efficient activation of mitophagy A, BFluorescence microscopy images (A) and quantification (B) of mitophagy of HeLa wild‐type (WT), PentaKO + empty vector, PentaKO + NDP52 WT or NDP52 C18, 153, 163, 321S (Mut) cells, stably expressing YFP‐Parkin and mt‐mKeima were pre‐treated with or without MitoQ for 22 h and treated with 4 μM/10 μM AO for the indicated times.CHeLa PentaKO + NDP52 WT or NDP52 Mut cells stably expressing YFP‐Parkin and mt‐mKeima were treated with 4 μM/10 μM AO for 2 h in the absence or presence of 10 μM MG132 (MG) and 400 nM Baf, followed by a mitochondrial fractionation and immunoblotting for NDP52 in non‐reducing conditions. The mitochondrial protein UQCRC2 was used as a loading control.D, EHeLa PentaKO + NDP52 WT cells stably expressing YFP‐Parkin and mt‐mKeima were treated with 1 mM DFP for 24 h (D), or pre‐treated with 500 nM MitoQ for 22 h and treated with 4 μM/10 μM AO for 2 h (D, E) in the presence or absence of 400 nM Baf (E). Cells were then analysed by MitoSOX staining (D) and immunoblotting for NDP52 in reducing conditions (E). Data information: #, non‐specific band or a post‐translational modification. Data are mean ± s.e.m. (B, D) or displayed as cell popular violin plots (B). *P* values were calculated by one‐way ANOVA followed by Sidak test on three independent experiments (B, D). *, *P* < 0.05; **, *P* < 0.01; ***, *P* < 0.001; §, *P* < 0.05, §§§, *P* < 0.001 (relative to MitoQ‐untreated condition); ns (non‐significant). Scale bars: 20 μm (A, D). Source data are available online for this figure.

In basal state, the levels of mitophagy in control HeLa are low and similar to those in PentaKO (Lazarou *et al*, 2015; Padman *et al*, 2019). Complete depolarisation of mitochondria and induction of mitophagy can be achieved by a combination of 4 μM antimycin and 10 μM oligomycin (AO) in this experimental model (Lazarou *et al*, 2015). Consistent with previous data, extensive mitophagy was observed in CRISPR control, but not in PentaKO, cells 1 h after the treatment and further increased in a time‐dependent manner (Fig 4A and B). The expression of wild‐type NDP52 was sufficient to rescue the mitophagy defect in PentaKO cell line as previously reported (Fig 4A and B). However, cells expressing the oxidation‐insensitive NDP52 mutant showed a significant delay in the induction mitophagy, which was only elevated 3 h after the treatment (Fig 4A and B). A similar retardation of the mitophagy kinetics was observed in response to a lower concentration of AO (1 μM antimycin and 1 μM oligomycin), confirming the inability of the NDP52 mutant to drive rapid clearance of damaged mitochondria (Appendix Fig S3). AO treatment also induced the formation of wild type, but not mutant, NDP52 DLC which were stabilised in mitochondrial fractions of cells treated with bafilomycin A1 supporting the conclusion that mitophagy is driven by oxidation and oligomerisation of NDP52 (Fig 4C). Analysis of the individual redox‐sensitive residues indicated an additive effect of Cys mutations on the ability of NDP52 to promote mitophagy consistent with the role of all four residues in this process (Fig EV2H).

**Figure EV3:**
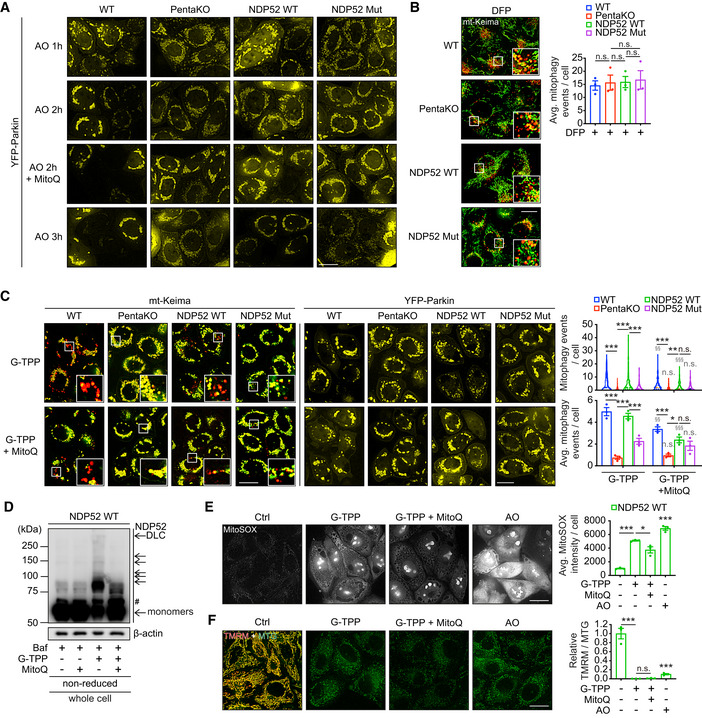
Oxidation of NDP52 facilitates PINK1/Parkin mitophagy with no role in mitophagy triggered by iron chelation AFluorescence images of YFP‐Parkin in HeLa WT, PentaKO, PentaKO + NDP52 WT and PentaKO + NDP52 Mut cells stably expressing YFP‐Parkin and mt‐mKeima, in the same fields and conditions as Fig 4A.BFluorescence microscopy images and quantification of mitophagy of HeLa WT, PentaKO, PentaKO + NDP52 WT and PentaKO + NDP52 Mut cells stably expressing YFP‐Parkin and mt‐mKeima were treated with 1 mM DFP for 24 h.CFluorescence microscopy images and quantification of mitophagy of HeLa WT, PentaKO, PentaKO + NDP52 WT and PentaKO + NDP52 Mut cells stably expressing YFP‐Parkin and mt‐mKeima, pre‐treated with or without 500 nM MitoQ for 16 h and treated with 10 μM G‐TPP for 8 h.D–FHeLa PentaKO + NDP52 WT cells were pre‐treated with or without MitoQ for 16 h and treated with G‐TPP for 5 h or AO for 2 h, followed by immunoblotting for NDP52 in non‐reducing conditions (D), MitoSOX staining (E) and mitochondrial membrane potential assay by using TMRM and Mitotracker Green (MTG) staining (F). Data information: Data are mean ± s.e.m. (B, C, E, F) or displayed as cell popular violin plots (C). *P* values were calculated by one‐way ANOVA followed by Sidak test on three independent experiments (B, C, E, F). *, *P* < 0.05; **, *P* < 0.01; ***, *P* < 0.001; §§, *P* < 0.01; §§§, *P* < 0.001 (relative to MitoQ‐untreated condition); ns (non‐significant). Scale bars: 20 μm (A, B, C, E, F). Source data are available online for this figure.

The levels of ROS as measured by MitoSOX were significantly elevated in response to AO treatment suggesting that oxidation of NDP52 can be triggered by ROS produced by damaged mitochondria (Fig 4D). To test this, we used a mitochondria‐targeted antioxidant MitoQ that potently suppressed the ROS levels in AO‐treated cells (Fig 4D; Smith & Murphy, 2010). Treatment with MitoQ also prevented NDP52 DLC formation in these conditions indicating that oxidation of NDP52 is mediated by ROS produced by mitochondria (Fig 4E). Importantly, MitoQ also completely blocked AO‐induced mitophagy mediated by NDP52 and cancelled the differences between NDP52 wild type, NDP52 mutant and PentaKO cells without affecting Parkin recruitment to mitochondria (Figs 4A and B, and EV3A). Interestingly, mitophagy was still induced by AO in CRISPR control HeLa cells in the presence of MitoQ, albeit to a lower level (Fig 4A and B), suggesting that other mitophagy SARs such as OPTN, which did not display redox sensitivity in our experiments (Fig EV1B), may act in ROS‐independent but depolarisation‐dependent manner. Additionally, indirect evidence for the requirement of ROS in driving damage‐induced SAR‐dependent mitophagy came from using an alternative method of mitophagy stimulation, an iron chelator deferiprone (DFP; Allen *et al*, 2013). Treating cells with DFP for 24 h did not stimulate ROS production but strongly activated mitophagy (Figs 4D and EV3B). However, the DFP‐induced mitophagy was not affected by the loss of NDP52 redox sensing or indeed by the loss of NDP52 and other SARs (Fig EV3B). Finally, treatment with G‐TPP triggering accumulation of misfolded proteins in mitochondria was used as an alternative stimulus for mitophagy (Fiesel *et al*, 2017). Similar to AO, G‐TPP induced ROS production, loss of membrane potential, recruitment of Parkin, NDP52 DLC formation and NDP52 oxidation‐dependent mitophagy (Fig EV3C–F). Together, our data indicate that elevated ROS due to mitochondrial damage resulting from the block in mitochondrial electron transport chain or accumulation of mitochondrial misfolded proteins triggers oxidation and oligomerisation of NDP52 which are required for the efficient clearance of damaged mitochondria.

### NDP52 is oxidised by ROS produced by mitochondrial Complex I and III

**Figure 5:**
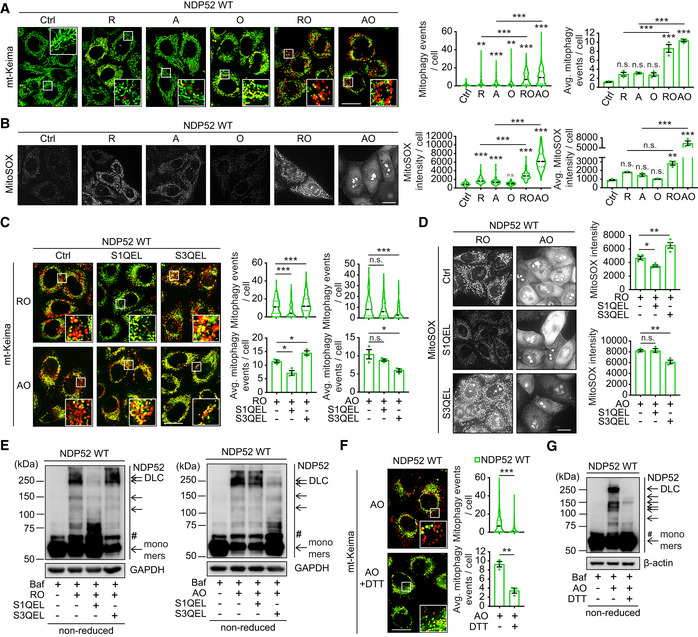
NDP52 oxidation is mediated by ROS produced by mitochondrial CI and CIII AFluorescence microscopy images and quantification of mitophagy in HeLa PentaKO + NDP52 WT cells stably expressing YFP‐Parkin and mt‐mKeima were treated with 4 μM rotenone (R), 4 μM A, 10 μM O, or the combination of RO or AO for 3 h.BFluorescence microscopy images and quantification of MitoSOX staining in HeLa PentaKO + NDP52 WT cells in the same conditions as (A).C–EHeLa PentaKO + NDP52 WT cells (D) or cells stably expressing YFP‐Parkin and mt‐mKeima (C, E) were pre‐treated with or without 10 μM S1QEL or 20 μM S3QEL for 30 min and treated with AO for 2 h or with RO for 3 h, followed by mitophagy measurement (C), MitoSOX staining (D) and immunoblotting for NDP52 in non‐reducing conditions (E).F, GHeLa PentaKO + NDP52 WT stably expressing YFP‐Parkin and mt‐mKeima were pre‐treated with or without 10 mM DTT for 30 min and treated with AO for 2 h followed by mitophagy measurements (F) and immunoblotting for NDP52 in non‐reducing conditions (G). Data information: #, non‐specific band or a post‐translational modification. Data are mean ± s.e.m. (A, B, C, D, F) or displayed as cell popular violin plots (A, B, C, F). *P* values were calculated by one‐way ANOVA followed by Sidak test (for multiple groups) or unpaired two‐tailed Student's *t*‐test (for two groups) on at least three independent experiments as indicated (A, B, C, D, F). *, *P* < 0.05; **, *P* < 0.01; ***, *P* < 0.001; ns (non‐significant). Scale bars: 20 μm (A, B, C, D, F). Source data are available online for this figure.

**Figure EV4:**
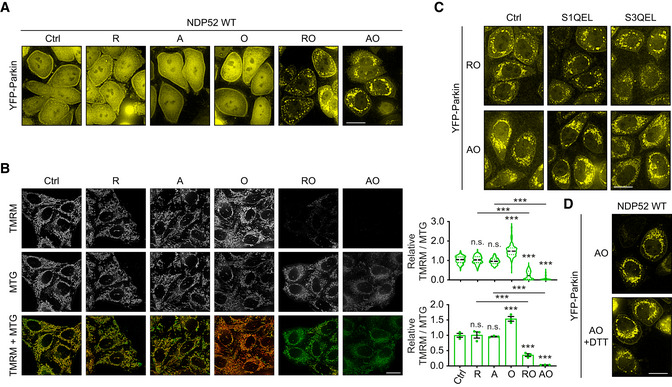
Recruitment of Parkin and mitochondrial membrane potential upon mitochondrial damage induced by mitochondrial inhibitors AFluorescence images of YFP‐Parkin in HeLa PentaKO + NDP52 WT cells stably expressing YFP‐Parkin and mt‐mKeima, in the same fields and conditions as Fig 5A.BFluorescence images of HeLa PentaKO + NDP52 WT cells treated with 4 μM R, 4 μM A, 10 μM O, or the combination of RO or AO for 3 h, followed by mitochondrial membrane potential assay by using TMRM and MTG staining.C, DFluorescence images of YFP‐Parkin in HeLa PentaKO + NDP52 WT cells stably expressing YFP‐Parkin and mt‐mKeima, in the same fields and conditions as Fig 5C and F, respectively. Data information: Data are mean ± s.e.m. or displayed as cell popular violin plots (B). *P* values were calculated by one‐way ANOVA followed by Sidak test on three independent experiments (B). ***, *P* < 0.001; ns (non‐significant). Scale bars: 20 μm (A–D).

Mitochondrial Complex I and III are major sources of ROS production (Murphy, 2009). To interrogate the origin of ROS triggering NDP52 oxidation and mitophagy, we used Complex I inhibitor rotenone and compared it to Complex III inhibitor antimycin. In the absence of oligomycin, which blocks mitochondrial respiratory chain at the level of Complex V, neither rotenone nor antimycin were able to cause the loss of membrane potential and strongly increase ROS, Parkin recruitment and mitophagy (Figs 5A and B, and EV4A and B). In contrast, addition of oligomycin to rotenone or antimycin resulted in the significant membrane depolarisation, production of ROS, Parkin recruitment and mitophagy induction suggesting that both Complex I and III can act as sources of redox signal for mitophagy (Fig 5A and B). To test this, we used S1QEL and S3QEL to suppress ROS production by Complex I and III, respectively (Brand *et al*, 2016). As a result, S1QEL selectively suppressed the mitophagy, ROS production and DLC formation of NDP52 induced by rotenone, whilst S3QEL abrogated these phenotypes triggered by antimycin (Fig 5C–E). These data indicated that NDP52 is able to form DLC and promote mitophagy in response to ROS produced by either Complex I or III. Treatment of cells with reducing agent DTT confirmed that the formation of disulphide bonds was essential for mitophagy induction as it suppressed both NDP52 DLC and mitophagy (Fig 5F and G). At the same time, Parkin recruitment was unaffected by the suppression of ROS signalling indicating that redox regulation of mitophagy takes place downstream of membrane depolarisation and PINK1/Parkin activation (Fig EV4C and D).

### NDP52 oxidation facilitates recruitment of mitophagy machinery upon mitochondrial damage

**Figure 6:**
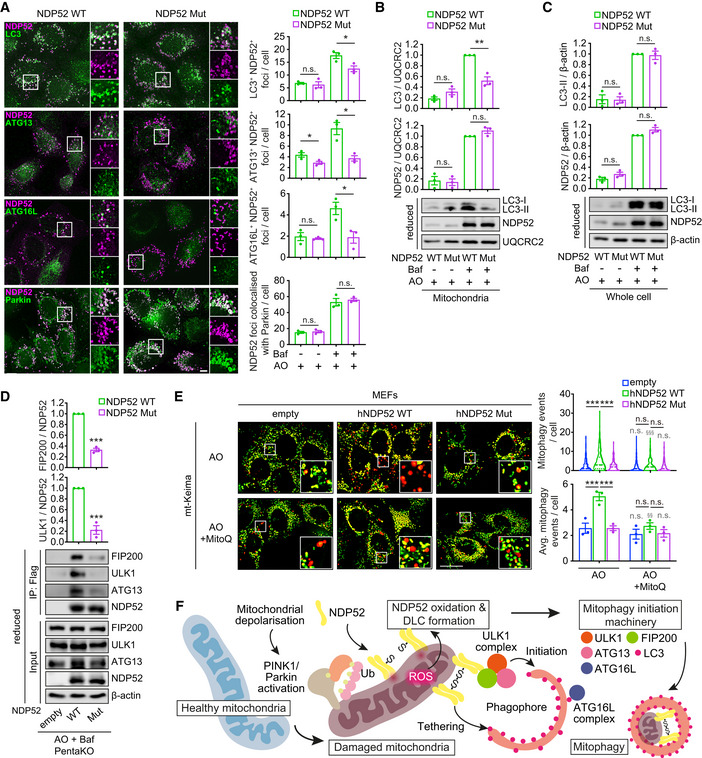
Oxidation of NDP52 facilitates the recruitment of autophagy proteins to damaged mitochondria A–DHeLa PentaKO + NDP52 WT or NDP52 Mut cells stably expressing YFP‐Parkin and mt‐mKeima were treated with 4 μM/10 μM AO for 2 h in the presence or absence of 400 nM Baf, followed by immunofluorescence analyses (A), immunoblotting for LC3 and NDP52 in mitochondrial fraction (B) or whole cell lysate (C) in reducing conditions, and co‐immunoprecipitation assay to analyse the interaction of NDP52 with FIP200, ULK1 and ATG13 (D). The number of foci of the indicated proteins colocalised with NDP52, or foci of NDP52 colocalised with Parkin, was quantified.EMEFs stably expressing YFP‐Parkin, mt‐mKeima and empty, human NDP52 (hNDP52) WT or hNDP52 Mut were pre‐treated with or without 500 nM MitoQ for 21 h and treated with AO for 3 h followed by mitophagy measurement.FSchematic representation of the mechanism of the damage‐induced mitophagy mediated by NDP52 DLC. Data information: Data are mean ± s.e.m. (A–E) or displayed as cell popular violin plots (E). *P* values were calculated by one‐way ANOVA followed by Sidak test (for multiple groups) or unpaired two‐tailed Student's *t*‐test (for two groups) on three independent experiments (A–E). *, *P* < 0.05; **, *P* < 0.01; ***, *P* < 0.001; §§, *P* < 0.01; §§§, *P* < 0.001 (relative to MitoQ‐untreated condition); ns (non‐significant). Scale bars: 20 μm (A, E). Source data are available online for this figure.

**Figure EV5:**
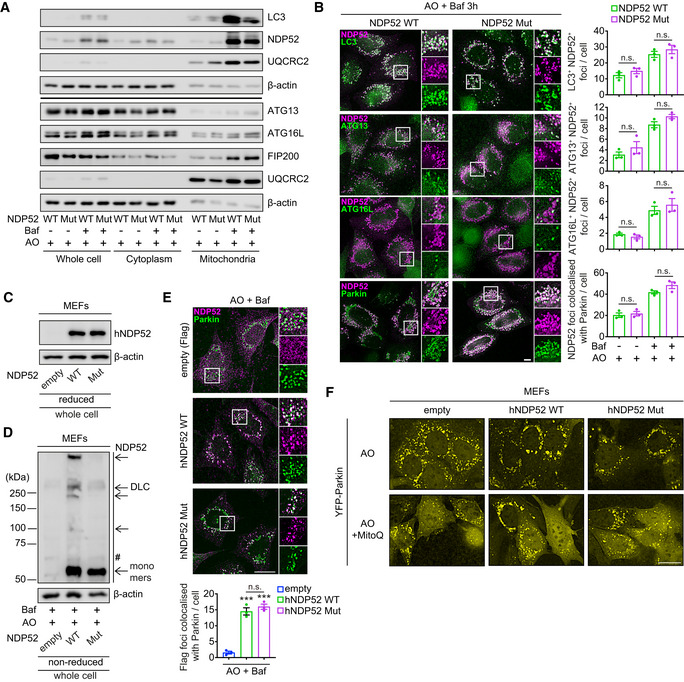
Recruitment of autophagy/mitophagy proteins to mitochondria in HeLa cells and MEFs AHeLa PentaKO + NDP52 WT and PentaKO + NDP52 Mut cells stably expressing YFP‐Parkin and mt‐mKeima, in the same conditions as Fig 6B and C, followed by immunoblotting for the indicated proteins in whole cell lysate, cytoplasmic and mitochondrial fractions in reducing conditions. Note that the LC3, NDP52 and actin blots of whole cell lysate are from the same membranes as those shown in Fig 6C.BHeLa PentaKO + NDP52 WT or NDP52 Mut cells stably expressing YFP‐Parkin and mt‐mKeima were treated with AO for 3 h in the presence or absence of 400 nM Baf, followed by immunofluorescence analyses.C, DMEFs stably expressing YFP‐Parkin, mt‐mKeima and empty (Flag), Flag‐human NDP52 WT (hNDP52) or Flag‐hNDP52 Mut were analysed by immunoblotting for the expression level of hNDP52 (C) and DLC formation upon AO/Baf treatment for 3 h (D) in reducing (C, 2.5% β‐mE) or non‐reducing (D) conditions.EMEFs stably expressing YFP‐Parkin, mt‐mKeima and empty (Flag), Flag‐hNDP52 WT or Flag‐hNDP52 Mut were treated with AO and Baf for 3 h followed by immunofluorescence analyses.FFluorescence images of YFP‐Parkin in MEFs stably expressing YFP‐Parkin, mt‐mKeima and empty (Flag), Flag‐hNDP52 WT or Flag‐hNDP52 Mut, in the same fields and conditions as Fig 6E. Data information: The number of foci of the indicated proteins colocalised with NDP52, or foci of NDP52 colocalised with Parkin, was quantified (B, E). Data are mean ± s.e.m. (B, E). *P* values were calculated by unpaired two‐tailed Student's *t*‐test (B) or one‐way ANOVA followed by Sidak test (E) on three independent experiments. ***, *P* < 0.001 (relative to MEFs transduced with an empty vector); ns (non‐significant). Scale bars: 20 μm (B, E, F). Source data are available online for this figure.

Therefore, we sought to identify the step in the molecular cascade leading to mitophagy induction affected by the loss of NDP52 redox sensing. Immunofluorescence and mitochondrial fractionation analyses indicated that both wild type and mutant NDP52 were equally translocated to damaged mitochondria as assessed by the colocalisation with Parkin 2 h after AO treatment in the presence of bafilomycin A1 (Figs 6A and B, and EV5A). These data indicate that the recruitment of Parkin or NDP52 to damaged mitochondria is not regulated by oxidation of NDP52. However, mutant NDP52 failed to efficiently recruit autophagy initiation components ATG13 and ATG16L, which was evident from the immunofluorescence analyses after 2 h after AO/bafilomycin A1 treatment (Fig 6A). Similarly, a significant reduction of LC3 localisation to NDP52‐positive mitochondria was observed in cells expressing mutant NDP52 (Fig 6A). This lower recruitment of LC3 to mitochondria was also evident in mitochondria‐enriched fractions from cells expressing mutant NDP52, whereas the levels of lipidated LC3 and NDP52 in whole cell lysate were unaffected by NDP52 mutation (Figs 6B and C, and EV5A). In a previous report, NDP52 was shown to bind ULK1 complex through the interaction with FIP200 (Vargas *et al*, 2019). However, we did not observe an accumulation of autophagy initiation factors FIP200, ATG13 and ATG16L in mitochondrial fractions by immunoblotting after AO/bafilomycin A1 treatment suggesting low levels or transient association of these proteins with mitochondria (Fig EV5A). Therefore, we instead investigated their interaction with NDP52 by immunoprecipitation assays which showed that Cys mutant was able to bind less ULK1 complex components, FIP200, ULK1 and ATG13 compared to wild type NDP52 during damage‐induced mitophagy (Vargas *et al*, 2019; Fig 6D). Together, these data suggest that NDP52 oxidation and oligomerisation facilitate recruitment of autophagy proteins, potentially by increasing binding avidity (Turco *et al*, 2019). Consistent with mitophagy levels becoming indistinguishable in cells expressing wild type and mutant NDP52 3 h after AO treatment, recruitment of mitophagy initiation machinery at this timepoint was also normalised (Figs 4A and B, and EV5B). We conclude that oxidation and oligomerisation of NDP52 on damaged mitochondria promote the rate and efficiency of mitophagy initiation.

Finally, we were interested in the observation that redox sensitive Cys residues in NDP52 became acquired late during human evolution with all four residues present only in apes (with the exception of wolf (*Canis lupus*)) whilst being absent for example in mice (Fig 2F). We therefore undertook a reverse engineering approach where we introduced human NDP52 into mouse embryonic fibroblasts (MEFs) and investigated redox regulation of mitophagy. Both wild type and mutant human NDP52 were expressed at similar levels and were recruited to mitochondria in mouse cells upon AO treatment, whilst only wild‐type protein was able to form DLC in these conditions (Fig EV5C–E). Expression of wild type but not mutant human NDP52 increased mitophagy events in MEFs, which was completely suppressed by MitoQ, indicating that introduction of human protein was sufficient to initiate ROS‐dependent mitophagy in mouse cells (Fig 6E). As in human cells, neither expression of NDP52 nor treatment with mitochondria‐targeted antioxidant affected recruitment of Parkin to mitochondria (Fig EV5F). In conclusion, redox sensing during mitophagy is mediated by NDP52 and has evolved in humans as a mechanism of oxidative stress response.

## Discussion

Several mechanisms linking increased ROS production by dysfunctional mitochondria to its elimination by mitophagy have been proposed, although the topic remains controversial (Sedlackova *et al*, 2020). This is particularly true in case of PINK1/Parkin‐dependent mitophagy triggered by the loss of mitochondrial membrane potential. Whilst the ROS produced following mitochondrial damage has long been discussed as a potential contributor to mitophagy induction, the molecular mechanism of ROS sensing in this pathway remains unknown (Sedlackova & Korolchuk, 2019). Here we identified a receptor protein NDP52 as a specific mediator of the ROS signal produced by dysfunctional mitochondria and an executor of their sequestration into autophagic vesicles for elimination. Oxidation of NDP52 on mitochondria results in its oligomerisation mediated by disulphide bonds which enhances the recruitment of autophagy initiation machinery (Fig 6F). As such, similar to the mechanism previously proposed by us for p62, NDP52 DLC formation can be viewed as a post‐translational modification activating its function as a SAR (Carroll *et al*, 2018; Otten *et al*, 2018). Importantly, unlike in case of p62 where the endogenous source of ROS signal remains to be identified, NDP52 is clearly activated by the ROS produced by the aberrant activity of the electron transport chain, namely CI and CIII, within depolarised mitochondria. Notably, CI and CIII produce superoxide that is highly membrane‐impermeable (Ungvari *et al*, 2007), which may limit the activity of NDP52 oxidation‐mediated mitophagy initiation at basal state. Indeed, NDP52‐mediated mitophagy required mitochondrial depolarisation which is associated with the opening of mitochondrial permeability transition pore leading to the release of ROS (superoxide and H_2_O_2_) from the mitochondria (Zorov *et al*, 2014). Given that MitoSOX is a selective superoxide sensor, we speculate that NDP52 is oxidised primarily by superoxide released from depolarised mitochondria, in cytosol and on the surface of the organelle. Interestingly, p62 is also recruited to depolarised mitochondria however, unlike NDP52, does not form DLC that are degraded via mitophagy, despite overall higher sensitivity to other ROS triggers, such as exogenously added hydrogen peroxide or a redox cycler PR‐619 (Fig EV1B; Lazarou *et al*, 2015). This indicates a remarkable selectivity in the ability of different SARs to sense specific ROS sources or species. The reason for this highly specific response remains unknown and is an exciting avenue for future investigations. Furthermore, a recent study reported the regulation of PINK1 activity by oxidation (Gan *et al*, 2022). Whereas we could not observe the impact on PINK1 activity assessed by Parkin recruitment in our experimental conditions, further investigations into the coordination of response to ROS by PINK1/Parkin and SARs will provide more precise understanding of mitophagy mechanisms.

Additionally, our studies provide further support to the concept of selective autophagy as a mechanism of oxidative stress response (Sedlackova *et al*, 2020). DLC of SARs such as NDP52 and p62 are stabilised in the presence of thioredoxin reductase inhibitors or autophagy blockers (Fig 1B; Carroll *et al*, 2018). The implications of these findings are twofold. First, DLC are continuously formed in response to localised ROS sources but are efficiently and rapidly reduced by the thioredoxin reductase antioxidant system. Only in the conditions where the capacity of the antioxidant system is saturated, defined as an oxidative stress (Tomalin *et al*, 2016), DLC remain stable and instead are directed, together with the cargo they associate with, for autophagic elimination. This last line of oxidative stress response allows removal of the ROS source, in the case of NDP52, damaged mitochondria, and re‐establish the redox balance. As such, selective autophagy should be added to the repertoire of defensive mechanisms where the buffering capacity of other antioxidant systems is breached. One of the open questions that remains to be addressed in this regard is the role of peroxiredoxins and the previously proposed redox‐relay mechanism mediated by these highly abundant proteins (Sobotta *et al*, 2015). Based on the current model and similar to the majority of low‐abundance proteins in the cell, SARs would be oxidised indirectly where ROS initially reacts with a cysteine residue in peroxiredoxin family proteins, followed by the formation of a heterotypic (SAR‐peroxiredoxin) disulphide bond with subsequent formation of homotypic SAR DLC (Sobotta *et al*, 2015). Whether this mechanism indeed applies to SARs or, alternatively, whether they can act as direct sensors of ROS is currently unclear, particularly as DLC are evident in conditions of oxidative stress where reactive residues in peroxiredoxins are oxidised to cysteine‐sulfenic acid that cannot be involved in disulphide bond formation (Tomalin *et al*, 2016).

Finally, another important implication of these studies is for our understanding of selective autophagy as a mechanism promoting human health and longevity. We have previously found that redox‐sensitive cysteine residues in p62 are only conserved in vertebrates and hypothesised that the ability to sense ROS acquired by this SAR during evolution may contribute to their increased stress‐tolerance and ultimately longevity. Indeed, introduction of redox‐sensing cysteines into the *Drosophila* orthologue of p62 lacking these residues allowed for an acquisition of the ability of the fruit fly to activate autophagy in response to stress and increased survival (Carroll *et al*, 2018; Otten *et al*, 2018). Interestingly, in contrast to PINK1/Parkin that are highly conserved in vertebrate and invertebrate species, the four cysteine residues involved in NDP52 DLC formation are acquired very late in the evolution of vertebrate species, where some are present in longer living but not in short‐lived species such as mouse and indeed mouse cells do not display redox regulation during mitophagy (Fig 6E). Furthermore, the co‐occurrence of all four cysteines is only seen in high primate species (except wolf, which may represent a case of convergent evolution; Fig 2F). It could be speculated that the ability to sense oxidative stress and improved defence mechanisms mediated by selective autophagy co‐evolved with, and contributed to, longevity of our ancestors. Importantly, p62 has previously been identified as a potential target for the activation by small molecules that induce DLC formation and promote p62‐dependent selective autophagy (Cha‐Molstad *et al*, 2017). NDP52 may potentially serve as a new therapeutic target where identification of drugs that can facilitate the mechanism identified in our study could provide the means to improve mitochondrial quality control by selective autophagy, help to combat diseases associated with the loss of cellular homeostasis and increase human healthspan.

## Materials and Methods

### Cell culture

HeLa (from European Collection of Cell Cultures), wild type (WT) and penta‐knockout (PentaKO) HeLa (Lazarou *et al*, 2015), WT and PentaKO HeLa stably expressing YFP‐Parkin and mt‐mKeima (Lazarou *et al*, 2015) and HEK293FT (Thermo Fisher Scientific) cells were grown in DMEM (Sigma‐Aldrich, D6546) supplemented with 10% foetal bovine serum (FBS; Sigma‐Aldrich), 100 U/ml penicillin/streptomycin (Sigma‐Aldrich) and 2 mM L‐glutamine (Sigma‐Aldrich) in a humidified atmosphere containing 5% CO_2_ at 37°C. Cells were treated with the following compounds and drugs at different concentrations and time‐points as indicated; hydrogen peroxide (H_2_O_2_; Sigma‐Aldrich, H1009), PR‐619 (LifeSensors, S19619), curcumin (Sigma‐Aldrich, C1386), auranofin (Sigma‐Aldrich, A6733), antimycin A (Sigma‐Aldrich, A8674), oligomycin (Merck Millipore, 495455), bafilomycin A1 (Enzo Life Sciences, BML‐CM110‐0100), MG132 (Sigma‐Aldrich, C2211), deferiprone (DFP; Sigma‐Aldrich, 379409), Gamitrinib TPP hexafluorophosphate (G‐TPP, Insight Biotechnology Ltd, HY‐102007A), MitoQ (gift from Michael Murphy), dithiothreitol (DTT, Thermo Scientific, R0861), S1QEL2.2 (Life Chemicals, F2068‐0013) and S3QEL 2 (Sigma‐Aldrich, SML1554). Medium was switched to serum‐free DMEM for the duration of H_2_O_2_ or PR‐619 treatments.

### Alignment of protein sequences

Alignment of NDP52 protein sequences was carried out as described previously (Carroll *et al*, 2018). In brief, NDP52 protein sequences in 12 organisms were identified by searching UniProt. Multiple Sequence alignment was carried out using the Muscle server at EBI (http://www.ebi.ac.uk/Tools/msa/muscle/) with default parameters. The resulting alignment was visualised using ALINE (Bond & Schuttelkopf, 2009). Conservation is indicated by depth of 10 colour from light to dark red, with conservation below 30% indicated in white.

### Cloning and mutagenesis

pDEST26/His‐Flag‐NDP52 was generated by Gibson assembly. Using Flag‐NDP52 M5P (gift from Dr. Felix Randow, Ravenhill *et al*, 2019) and pDEST26/OPTN (Addgene #23050, Zhu *et al*, 2007) as a template, Flag‐NDP52 and pDEST26 were amplified by PCR with PfuUltra II Fusion HS DNA Polymerase (Agilent) and specific primers (Appendix Table S1). PCR products were separated by agarose gel electrophoreses, and excised fragments of interest were assembled using the NEBuilder HiFi DNA Assembly kit (New England Biolabs) according to the manufacturer's instructions. After the assembly, the reaction mix was transformed into α‐select GOLD Efficiency chemically competent cells (Bioline). The plasmid was extracted and purified using QIAprep Spin Miniprep Kit (Qiagen).

Point mutagenesis of the NDP52 gene was carried out using Q5 Site‐Directed Mutagenesis Kit (NEB) according to manufacturer's instructions. Mutagenesis primers were designed using the NEB primer design program (http://nebasechanger.neb.com; Appendix Table S2). The pDEST26/His‐Flag‐NDP52 plasmid was used as a template. PCR reactions were placed on a Veriti 96‐Well Thermal Cycler (Applied Biosystems) using the provided program: 1 cycle at 95°C for 1 min, 18 cycles (denaturation 95°C for 50 s, annealing 60°C for 50 s, extension 68°C for 7 min) and 1 cycle at 68°C for 6 min. Bacterial transformation was then carried out using XL10‐Gold® Ultracompetent Cells (Agilent Technologies). The NDP52 wild‐type construct was then subcloned into the pLENTI6/V5‐DEST (His‐Flag) vector for lentiviral expression by Gibson assembly as described above with specific primers (Appendix Table S3). To minimise the structural conformation change of NDP52 protein by point mutations, we further generated NDP52 constructs with mutations of Cys to Ser described as above using the pLENTI6/V5‐DEST/NDP52 and pETM30/NDP52 (Gift from Felix Randow, Ravenhill *et al*, 2019) for bacterial expression with specific primers (Appendix Table S4).

### Protein expression and purification

Expression and purification of GST‐NDP52 proteins were performed as described previously (Carroll *et al*, 2016). In brief, GST‐NDP52 wild‐type and mutants were expressed in Rosetta2 (DE3) *Escherichia coli* (Novagen), grown in 2xYT media and induced with 0.1 mM IPTG at 37°C for 2 h. Cells were then collected and lysed by sonication in 50 mM Tris, 150 mM NaCl buffer (pH 7.4). GST‐NDP52 proteins were purified by glutathione Sepharose 4B (GE Healthcare) affinity chromatography. Purified proteins were dialysed and concentrated by using Amicon Ultra‐4 (Millipore) and flash‐frozen in liquid nitrogen and stored at −80°C. Protein concentrations were measured using a Nanodrop 1000 spectrophotometer (Thermo Scientific) with extinction coefficients. Proteins were diluted to a concentration at 50 ng/μl in the buffer, and equal amounts of protein (5 μg) were exposed to H_2_O_2_ or AO and subjected to immunoblotting.

For the purification of untagged‐NDP52, the NDP52 wild‐type and NDP52 C18, 153, 163, 321S mutant constructs were subcloned into pET‐Duet1 vectors containing a C‐terminal TEV‐GST tag using Gibson Cloning kit (New England Biolabs). The proteins were expressed in *E. coli* Rosetta pLySS cells. Transformed cells were grown in 2xYT medium at 37°C until OD_600_ ~ 0.4 and then brought to 18°C. Protein expression was induced at OD_600_ ~ 0.8 with 0.05 mM IPTG and grown further for 16 h at 18°C. Cells were collected by centrifugation and resuspended in 50 mM Tris (pH 7.4), 300 mM NaCl, 1 mM DTT, 5% glycerol, 2 mM MgCl_2_, 2 mM β‐mercaptoethanol, DNase, 1× cOmplete EDTA‐free protease inhibitor cocktail (Roche). Cells were lysed by freeze thawing and 2 × 30 s sonication. Lysates were cleared by centrifugation (72,000 *g* for 45 min at 4°C), and the supernatant was incubated with 2 ml Glutathione Sepharose 4B beads slurry (Cytiva) for 2 h at 4°C. Beads were washed twice with low salt buffer (50 mM Tris pH 7.4, 300 mM NaCl, 1 mM DTT) followed by one wash with high salt buffer (50 mM Tris pH 7.4, 700 mM NaCl, 1 mM DTT), and finally two low salt buffer washes. Beads were then incubated overnight with TEV protease at 4°C to remove the GST‐tag. The supernatant, containing the cleaved protein, was collected and filtered through a 0.45 μm syringe filter, concentrated using 30 kDa cut‐off Amicon filters and then applied onto a Superdex 200 Increase 10/300 GL column (Cytiva) pre‐equilibrated with SEC‐buffer (25 mM Tris pH 7.4, 150 mM NaCl, 1 mM DTT). Fractions containing pure proteins were pooled, concentrated and snap frozen in liquid nitrogen and then stored at −80°C.

Expression and purification of FIP200‐GFP were performed as described previously (Turco *et al*, 2021). In brief, to generate the FIP200‐GFP construct, we purchased the codon‐optimised FIP200 from Genscript and cloned it with the respective tags into pGB‐02‐03 (pGB‐GST‐3C‐FIP200‐GFP). The FIP200‐GFP construct was then used for expression in *Spodoptera frugiperda* cells (Sf9) using the Bac‐to‐Bac system. The bacmid DNA was obtained by amplification in DH10BacY cells and 2.5 μg of bacmid DNA was transfected into 1 × 10^6^ Sf9 insect cells using FuGene transfection reagent (Promega). About 7 days after transfection, the V0 virus was collected by taking the supernatant and used to produce to infect 30 ml of Sf9 cells. The V1 virus stock was collected 4–5 days after infection and, after filtering, stored at 4 degrees. Expression of FIP200‐GFP was achieved by infecting 1 l of Sf9 cells with 1 ml of V1 virus. Cells were harvested by centrifugation when they reached a viability of 90–95%. Cell pellets were washed with PBS, flash frozen in liquid nitrogen, and stored at −80°C until purification. For purification of FIP200‐GFP, cell pellets from 1 l culture were thawed and resuspended in 40 ml lysis buffer (50 mM HEPES pH 7.5, 300 mM NaCl, 1 mM MgCl_2_, 10% glycerol, 0.5% CHAPS, 5 U/ml Benzonase (Sigma), 1 mM DTT, CIP protease inhibitor (Sigma), and cOmplete EDTA‐free protease inhibitor cocktail (Roche)). Cells were disrupted with a Dounce homogeniser followed by 1 min sonication. Lysates were cleared by centrifugation (72,000 *g* for 45 min at 4°C in Beckman Ti45 rotor). The supernatant was collected and incubated with 5 ml pre‐equilibrated Glutathione Sepharose 4B beads (GE Healthcare) overnight at 4°C on a tube roller. Beads were then washed seven times in wash buffer (50 mM HEPES pH 7.5, 200 mM NaCl, 1 mM MgCl_2_, 1 mM DTT), and the protein was eluted by overnight incubation of the beads bound protein with preScision 3C protease in 10 ml wash buffer. The eluate was filtered through a 0.45 μm syringe filter and concentrated to a final volume of 500 μl using 100 kDa MWCO concentrators (Millipore). The concentrated sample was further purified by size‐exclusion chromatography on a Superose 6 Increase 10/300 column (GE Healthcare) in elution buffer (25 mM HEPES pH 7.5, 200 mM NaCl, 1 mM DTT).

### Analytical size‐exclusion chromatography (SEC)

Analytical gel filtration was performed on a Superose 6 Increase 3.2/300 column (GE Healthcare) pre‐equilibrated with SEC buffer (25 mM Tris–HCl pH 7.4, 150 mM NaCl, 1 mM DTT). Purified untagged NDP52 WT and mutant were injected at 40 μM, and samples were collected in 100 μl fractions. Fractions were analysed by SDS‐PAGE followed by Coomassie staining.

### FIP200 – NDP52 interaction assay

Microscopy based protein–protein interaction assays were performed using Glutathione Sepharose 4B beads (GE Healthcare) and mixing them with GST or GST‐4xUb to a final concentration of 5 μM. Beads were incubated with bait proteins at 4°C for 1 h on a tube roller and then washed three times with washing buffer (25 mM Tris–HCl pH 7.4, 150 mM NaCl, 1 mM DTT). Of the bait‐bound beads, 1 μl was transferred into the well of 384‐well glass‐bottom microplate (Greiner Bio‐One) prefilled with 20 μl of bead assay buffer (25 mM Tris–HCl pH 7.4, 150 mM NaCl, 1 mM DTT) and prey proteins (final concentrations of 500 nM NDP52 and 200 nM FIP200‐GFP). The samples were incubated for 4 h and imaged by Zeiss LSM 700 confocal microscope equipped with Plan Apochromat 20×/0.8 WD 0.55 mm objective.

For quantification of the microscopy bead assay, an in‐house developed Artificial Intelligence (AI) tool was used for detecting and quantifying GFP‐signal on the beads. In brief, signal intensities in images of beads were quantified by line profiles across beads and the difference between the minimum and maximum grey values along the lines was determined. To this end, the AI‐plugin was trained to detect beads using Cellpose (Stringer *et al*, 2021). Multichannel input images were split into individual TIFF images and passed to Cellpose (running in a Python environment). The labelled images were then reassembled into multichannel images, and circular regions of interest (ROI) are fitted to the segmented particles. A pre‐defined number of line profiles is then drawn automatically starting at the ROI centre and extending beyond the border of the circular ROI (representing the edge of a bead). A combined ROI containing all detected beads is used to exclude line profiles protruding into adjacent beads. Line profiles were then inspected manually to add missing beads or exclude misassigned profiles. The obtained profiles were then used to calculate the average fluorescence signal intensity per bead. Plots show the mean values for each bead and significance was calculated using a Student's *t*‐test.

### Transfection

Cells were seeded in 6‐, 12‐well plates or a 35 mm glass bottom dish, cultured for 24 h and transfected with Lipofectamine 2000 (Invitrogen) according to the manufacturer's instructions for 24 h prior to lysis or 48 h prior to fluorescence microscopy.

### Generating stable cell lines by lentiviral transduction

Stable expression of His‐Flag‐NDP52 and His‐Flag‐NDP52 C18, 153, 163, 321S was achieved though lentiviral transduction as previously described (Carroll *et al*, 2018). In brief, lentiviruses were generated by using HEK293FT packaging cells, the NDP52 lentiviral expression vectors and 3^rd^ generation packaging system plasmids (Thermo Fisher Scientific). HeLa PentaKO cells and MEFs were transduced with the viruses and selected with 8 μg/ml of blasticidin (Thermo Fisher Scientific). Stable cell lines were maintained in lower levels of blasticidin (4 μg/ml) until seeding for experimental purposes.

### Immunoblot analysis

Immunoblotting was performed as described previously (Carroll *et al*, 2018). In brief, cells were lysed in RIPA buffer (Sigma‐Aldrich) supplemented with 1× Halt™ protease and phosphatase inhibitor cocktail (Thermo Fisher Scientific) and 50 mM N‐ethylmaleimide. Protein concentration was measured using DC Protein Assay (Bio‐Rad), and samples were prepared by boiling in Laemmli sample buffer (Bio‐rad) in the presence (reduced) or absence (non‐reduced) of 2.5% β‐mercaptoethanol. Equal amounts of protein (20–40 μg) were subjected to SDS‐PAGE and transferred to PVDF membranes. Membranes were first blocked in 5% milk (Merck Millipore) in PBS with 1× Tween® 20 (Sigma‐Aldrich) for 1 h at room temperature and incubated with primary antibodies overnight at 4°C. Secondary antibodies conjugated to horseradish peroxidase (HRP) for rabbit (Sigma‐Aldrich, A0545), mouse (Sigma‐Aldrich, A2554) or guinea pig (Dako, P0141) were used at 1:5,000 dilution for 1 h at room temperature. Chemiluminescence detection was achieved using Clarity Western ECL Substrate (Bio‐Rad) and a LAS‐4000 CCD camera system (Fujifilm). The following primary antibodies were used: guinea pig α‐p62 (Progen Biotechnik, GP62‐C, 1:2,000), rabbit α‐NDP52 (CST, 60732, 1:1,000), rabbit α‐OPTN (Abcam, ab23666, 1:1,000), rabbit α‐PRDX3 (Olahova *et al*, 2008, 1:1,000), rabbit α‐PRDX‐SO_3_ (Abcam, ab16830, 1:2,000), rabbit α‐ATG5 (Sigma‐Aldrich, A0856, 1:1,000), α‐ATG7 (CST, 8558S, 1:1,000), α‐TFEB (CST, 4240S, 1:1,000), α‐S6 (CST, 2217S, 1:1,000), rabbit α‐LC3 (CST, 3868S, 1:1,000), rabbit α‐Atg13 (CST, 13468S, 1:1,000), rabbit α‐Atg16L (CST, 8089S, 1:1,000), rabbit α‐FIP200 (CST, 12436S, 1:1,000), rabbit α‐ULK1 (CST, 8054T, 1:1,000), mouse α‐β‐actin (St John's Laboratory, STJ96930, 1:5,000), mouse α‐GAPDH (St John's Laboratory, STJ96931, 1:5,000) and mouse α‐UQCRC2 (Abcam, ab14745, 1:1,000). Densitometry analyses of immunoblots were done using ImageJ (version 1.48; NIH).

### BN‐page

Cells seeded in 6‐well plates were washed with ice‐cold PBS, lysed with BN‐lysis buffer (20 mM Bis‐Tris, pH 7.2, 40 mM NaCl, 10% glycerol, 0.5% NP‐40 and 0.5 mM PMSF) for 10 min on ice. Lysates were cleared by centrifugation for 10 min at 16,100 *g* at 4°C. Protein concentration was measured using DC Protein Assay (Bio‐Rad), and 30 μg (cell lysate) or 100 ng (recombinant) protein were mixed with equal volume of Coomassie brilliant blue G‐250 solution (0.17% G‐250, 20 mM BisTris pH7.2, 10% glycerol), and separated in a 4–16% NativePAGE gel (Invitrogen, BN1002) followed by immunoblotting as described above.

### Immunoprecipitation

Cells seeded in 10 cm dishes were washed with ice‐cold PBS and lysed with CoIP‐lysis buffer (150 mM Tris, pH 7.5, 50 mM NaCl, 0.5% Triton X‐100 and 1× Halt™ protease and phosphatase inhibitor cocktail (Thermo Fisher Scientific)). Lysates were cleared and protein concentration was measured as above, and equal amount of protein (2.5 mg) were incubated with Flag M2 Magnetic Beads (Sigma‐Aldrich, M8823) for 4 h at 4°C on a rotating wheel. The beads were separated from the solution using magnet and washed three times with wash buffer (150 mM Tris, pH 7.5, 50 mM NaCl). The beads were then boiled for 5 min at 100°C in 2× Laemmli sample buffer (Bio‐rad) and the samples were subjected to immunoblot analysis. For the detection of immunoprecipitated NDP52, Clean‐Blot IP detection Reagent (Thermo Scientific, 21230) was used to reduce denatured IgG background bands.

### Mitochondrial fractionation

Cells were seeded in 10 cm dishes (two dishes per condition) and collected with ice‐cold PBS by centrifugation for 5 min at 800 *g* at 4°C. Cells were then resuspended in fractionation buffer (20 mM HEPES‐KOH pH 7.6 (Sigma‐Aldrich), 220 mM mannitol (Sigma‐Aldrich), 70 mM sucrose (Sigma‐Aldrich), 1 mM EDTA (Sigma‐Aldrich), 2 mM DTT (Thermo Fisher Scientific) and 0.5 mM PMSF (Sigma‐Aldrich)) and homogenised with 50 stroking using a dounce homogeniser (Thermo Fisher Scientific). Cell homogenates were centrifuged for 5 min at 800 *g* at 4°C to pellet cellular nuclei and membrane debris. Mitochondrial fraction was separated by centrifugation for 10 min at 16,100 *g* at 4°C. The pellet was resuspended in 100 μl fractionation buffer and subjected to immunoblot analysis.

### Fluorescence microscopy

Fluorescence images were obtained using an inverted DMi8 microscope (Leica) with a Plan‐Apochromat 63×/1.40 oil immersion lens, equipped with an ORCA‐Flash4v2.0 camera (Hamamatsu). Images were deconvolved using Huygens Essential software (version 20.10, Scientific Volume Imaging). Images were analysed in ImageJ (version 1.48; NIH), and quantification was performed on at least 50 cells per condition.

### MitoSOX staining

Cells seeded in a 35 mm glass bottom dish (MatTek) were stained with 2.5 μM mitoSOX (Invitrogen) for 10 min and washed three times with cell culture medium. Fluorescence images were obtained described as above. Fluorescence intensity was analysed as outlining single cells as regions of interest and calculation of the raw integrated density value per cell.

### Mitochondrial membrane potential assay

Cells grown in a 96‐well glass bottom plate (Greiner Bio‐One) were co‐stained with 16.7 nM tetramethylrhodamine methyl ester (TMRM, Invitrogen, T668) and 100 nM Mitotracker Green (MTG, Invitrogen, M7514) for 30 min at 37°C, Cells were washed with medium and imaged in a maintained atmosphere of 37°C and 5% CO_2_. TMRM and MTG raw integrated density values per cell were quantified using ImageJ (version 1.53c; NIH) by outlining single cells as regions of interest. Mitochondrial membrane potential was expressed as a ratio of TMRM to MTG.

### Mitophagy assay

Cells stably expressing YFP‐Parkin and mt‐mKeima were seeded in a 35 mm glass bottom dish. The live‐cell mt‐mKeima signal was obtained described as above. Mitophagy events were determined as following steps using ImageJ (version 1.53c; NIH). Images were masked by applying MaxEntropy threshold algorithm to the images obtained with 561 nm excitation to remove low red signal and background. Within the masks, signals of mt‐mKeima were adjusted by applying Enhanced Contrast plugin with saturated = 0.1, normalise, equalise options. Then, images were generated by subtracting the signal at a 480 nm excitation (reporting neutral pH‐environment) from the signal at a 561 nm excitation (reporting an acidic pH‐environment). Resulting images were binarised with the MaxEntropy threshold algorithm to extract mitolysosomes. The number of puncta per cell in images was quantified by outlining single cells (using YFP‐Parkin channels) as regions of interest and counted using Analyse Particles plugin without considering the size and circularity of objects.

### Immunofluorescence

Immunofluorescence analysis was performed as described previously (Carroll *et al*, 2018). In brief, cells seeded on coverslips in 24‐well plates were fixed in 3.7% formaldehyde in PBS for 7 min at room temperature and permeabilised in methanol for 4 min at −20°C. Cells were then blocked for 1 h in 5% normal goat serum (Sigma‐Aldrich) in PBS at room temperature and incubated with primary antibodies overnight at 4°C. Cells were washed three times and incubated with the appropriate secondary antibodies for 1 h at room temperature (Thermo Fisher Scientific, A31556 and A21235, 1:1,000). Cells were washed, and coverslips were mounted on slides with fluoroshield mounting medium (Abcam). Fluorescence images were obtained described as above. The number of puncta colocalised with NDP52 or with YFP‐Parkin per cell was quantified. The following primary antibodies were used: mouse α‐Flag (Sigma‐Aldrich, F3165, 1:1,000, for NDP52 staining), rabbit α‐LC3 (CST, 3868S, 1:250), rabbit α‐Atg13 (CST, 13468S, 1:100) and rabbit α‐Atg16L (CST, 8089S, 1:100).

### Molecular modelling of NDP52 oligomers

The structure of full‐length human NDP52 monomer has been predicted with AlphaFold 2.0 (Jumper & Hassabis, 2022). The SKICH domains were found in a very good agreement (RMSD < 2 Å) with crystal structures available (PDB codes: 3VVV, 3VVW, 5Z7A, 5Z7L and 7EAA). C‐terminally truncated oligomers (residues 1–354) were modelled using several orthogonal approaches: AlphaFold‐Multimer (preprint: Evans *et al*, 2022), CCFold (Guzenko & Strelkov, 2018), ClusPro‐Multimer (Kozakov *et al*, 2017) and CCBuilder 2.0 (Wood & Woolfson, 2018). Models were evaluated by Multicoil2 (Trigg *et al*, 2011) and Logicoil (Vincent *et al*, 2013). Interaction energies were estimated by INTAA 2.0 (Vymetal *et al*, 2021). Cysteine reactivity was estimated using PROPKA (Olsson *et al*, 2011). Visualisation of the models, calculations of interatomic distances and macromolecular surface electrostatic potentials were carried out using UCSF Chimera (Pettersen *et al*, 2004).

#### Molecular dynamics simulations

All simulations of oligomers (coiled‐coil domains) were carried out using GROMACS 2020 (Abraham *et al*, 2015). For the antiparallel and parallel coiled‐coiled tetramers, the AMBER99SB‐ILDN force field were chosen with TIP3P water model. The protein was placed in the centre of a cubic box with 1 nm distance to the edge, water and counter‐ions (Na^+^ and Cl^−^) were added to neutralise the total charge inside the box. Then, molecular mechanical energy minimisation was performed using steepest descent algorithm with a 0.01 nm step size, the energy minimisation stopped when the when the maximum force is below 1,000.0 kJ/mol/nm using Verlet cutoff. Next, two phases of equilibration were followed: first, 60 ps NVT with a 2 fs time step, velocity‐rescaling thermostat was used to heat and maintain the system at 300 K, all‐bonds were constrained using LINear Constraint Solver (LINCS); second, 60 ps NPT equilibration with a 2 fs time step, Parrinello‐Rahman isotropic coupling was used to control the 1 bar pressure, all‐bonds were constrained using LINCS. Finally, 100 ns MD production simulations were run with a 2 fs time step. The coordinates were saved every 10 ps. For the antiparallel C‐terminally truncated tetramers (residues 1–354), MARTINI coarse‐grained simulation (Marrink *et al*, 2007) was carried out. The energy‐minimised all‐atom model was converted into a coarse‐grained model using MARTINI version 2.2 (Monticelli *et al*, 2008; de Jong *et al*, 2013) and then placed in the centre of a cubic box. Each model was energy minimised in vacuum first using steepest descent algorithm with a 0.01 nm step size, the minimization stopped when the new step size was too small or reach a maximum force lower than 10 kJ/mol/nm using Verlet cutoff, then water and counter‐ions were added. The whole system was then energy minimised again using same parameters and equilibrated for 2 ns at 300 K temperature and 1 bar pressure. Finally, 500 ns simulation was carried out with a 20 fs time step.

### Statistical analysis

Data from at least three independent biological replicates are expressed as mean ± s.e.m (depicted by column graph scatter dot plot) or cell popular violin plots and analysed by two‐tailed, unpaired Student's *t*‐test (for two groups) or one‐way ANOVA followed by Sidak test using GraphPad Prism 8 software. A *P* value < 0.05 was considered significant. * or ^§^, *P* < 0.05; ** or ^§§^, *P* < 0.01; *** or ^§§§^, *P* < 0.001; ns (non‐significant). No sample size calculations were performed. Blinding was not applied to experiments.

## Author contributions

**Tetsushi Kataura:** Conceptualization; data curation; formal analysis; funding acquisition; investigation; methodology; writing – original draft; writing – review and editing. **Elsje G Otten:** Conceptualization; formal analysis; investigation; methodology; writing – review and editing. **Yoana Rabanal‐Ruiz:** Conceptualization; formal analysis; investigation; methodology; writing – review and editing. **Elias Adriaenssens:** Formal analysis; funding acquisition; validation; investigation; methodology; writing – review and editing. **Francesca Urselli:** Investigation. **Filippo Scialo:** Investigation. **Lanyu Fan:** Investigation; visualization; writing – review and editing. **Graham R Smith:** Investigation. **William M Dawson:** Investigation; visualization; methodology; writing – review and editing. **Xingxiang Chen:** Investigation. **Wyatt W Yue:** Resources; supervision. **Agnieszka K Bronowska:** Supervision; investigation; visualization; writing – review and editing. **Bernadette Carroll:** Resources; investigation; visualization; project administration; writing – review and editing. **Sascha Martens:** Resources; supervision; funding acquisition; investigation; writing – review and editing. **Michael Lazarou:** Resources; supervision; funding acquisition; investigation; writing – review and editing. **Viktor I Korolchuk:** Conceptualization; supervision; funding acquisition; writing – original draft; project administration; writing – review and editing.

## Disclosure and competing interests statement

VIK is a Scientific Advisor for Longaevus Technologies. SM is a member of the Scientific Advisory Board of Casma Therapeutics.

## Supporting information



Appendix

Expanded View Figures PDF

Source Data for Expanded View and Appendix

PDF+

Source Data for Figure 1

Source Data for Figure 2

Source Data for Figure 3

Source Data for Figure 4

Source Data for Figure 5

Source Data for Figure 6

## Data Availability

This study includes no data deposited in external repositories.
