# Supplementary material for: NDP52 acts as a redox sensor in PINK1/Parkin‐mediated mitophagy
Source: EMBO J. 2022 Dec 14;42(5):e111372. doi: 10.15252/embj.2022111372 (PMC9975939; doi:10.15252/embj.2022111372)
Supplement: Supplementary file 1 — Appendix [file EMBJ-42-e111372-s008.pdf]

NDP52 acts as a redox sensor in PINK1/Parkin-mediated mitophagy

Tetsushi Kataura, Elsje G. Otten, Yoana Rabanal-Ruiz, Elias Adriaenssens, Francesca Urselli, Filippo Scialo, Lanyu Fan, Graham R. Smith, Xingxiang Chen, Wyatt W. Yue, Agnieszka K. Bronowska, William M. Dawson, Bernadette Carroll, Sascha Martens, Michael Lazarou, Viktor I. Korolchuk

Appendix Contents

Appendix Figure S1 page 1

Appendix Figure S2 page 2

Appendix Figure S3 page 3

Appendix Table S1 page 4

Appendix Table S2 page 5

Appendix Table S3 page 6

Appendix Table S4 page 7

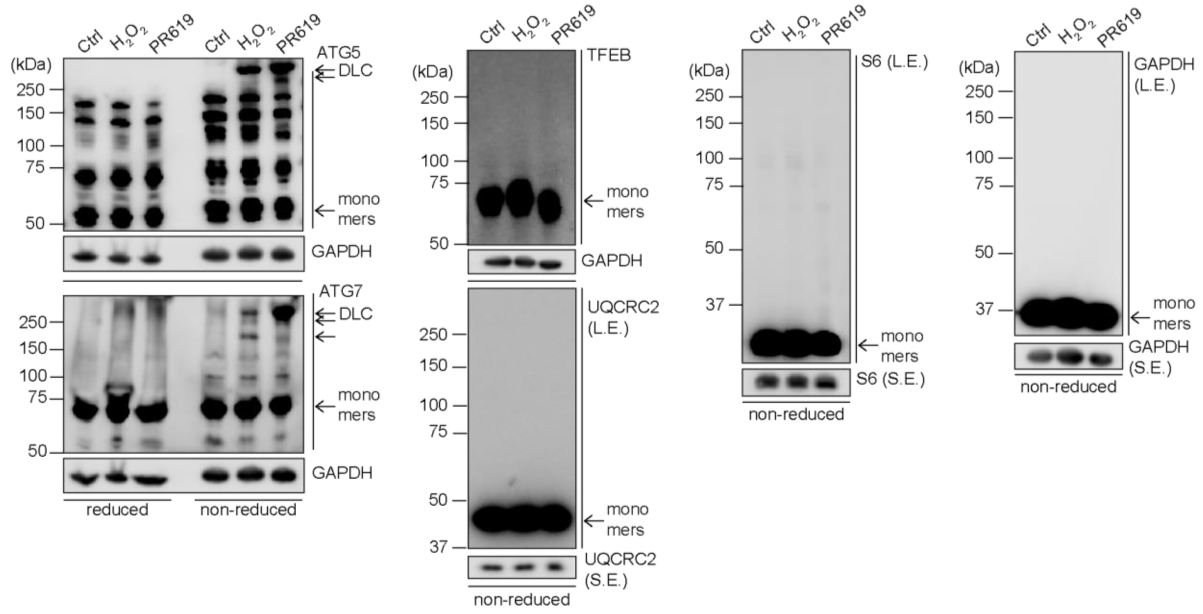

Appendix Figure S1. DLC formation of autophagy and non-autophagy proteins

HeLa cells were treated with H_2O_2 (5 mM, 1 min) or PR-619 (10 µM, 10 min) and analysed by immunoblotting for the indicated endogenous proteins in either reducing (2.5% β-mE) or non-reducing conditions. L.E., long exposure; S.E., short exposure.

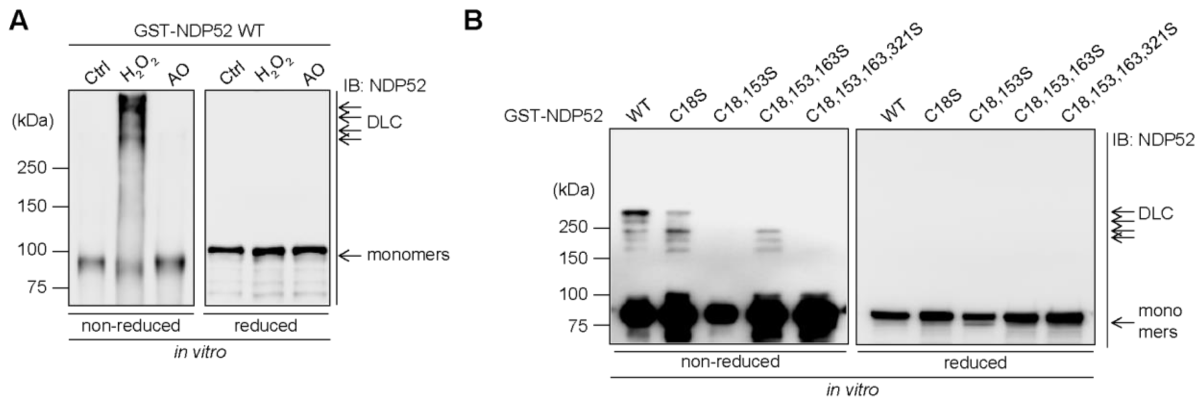

Appendix Figure S2. Recombinant NDP52 protein is oxidised by H_2O_2 but not by antimycin and oligomycin

A, B Recombinant GST-NDP52 WT (A, B) and GST-NDP52 mutants as indicated (B) were exposed with 4 µM / 10 µM AO for 3 h (A) or 5 mM H_2O_2 for 5 min (A, B) and analysed by immunoblotting in either reducing (2.5% β-mE) or non-reducing conditions.

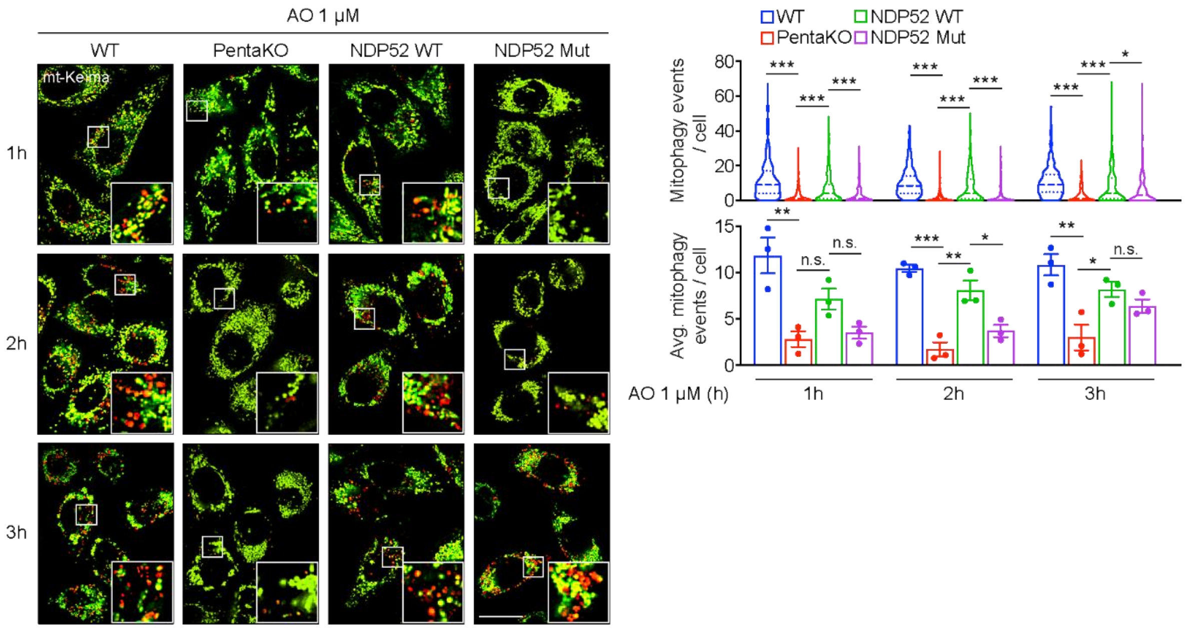

Appendix Figure S3. Activation of mitophagy by low dose of AO

Fluorescence microscopy images and quantification of mitophagy of HeLa WT, PentaKO + empty vector, PentaKO + NDP52 WT or NDP52 Mut cells stably expressing YFP-Parkin and mt-mKeima were treated with 1 µM / 1 µM AO for the indicated times. Data are mean ± s.e.m. or displayed as cell popular violin plots. *P* values were calculated by one-way ANOVA followed by Sidak test on three independent experiments. *, *p*<0.05; **, *p*<0.01; ***, *p*<0.001; ns (non-significant). Scale bars: 20 µm.

Appendix Table S1. Primers used to clone FLAG-NDP52 into pDEST26 vector

Primer	Sequence (5’-3’)
His-pDEST26-FLAG-NDP52 FWD	TGTTCTGCCACTCTCTCTGATTGATCGCGTGCATGCGACGTCA
His-pDEST26-FLAG-NDP52 REV	TCGTCGTCCTTGTAGTCGCCGTGATGGTGATGGTGATGGTAGTACGCCATGGTC
FLAG-NDP52-pDEST26 FWD	ACCATCACCATCACCATCACGGCGACTACAAGGACGACGACGACA
FLAG-NDP52-pDEST26 REV	CGTCGCATGCACGCGATCAATCAGAGAGAGTGGCAGAACACGTGGTCTTC

Appendix Table S2. Primers used for NDP52 mutagenesis (Cys to Ala)

Primer	Sequence (5'-3')
NDP52 C18A FWD	AGCTGTCTTGCTGGATCACGCTCATTTCTCTCAGGTCATC
NDP52 C18A REV	GATGACCTGAGAGAAATGAGCGTGATCCAGCAAGACAGCT
NDP52 C40A FWD	GTGAAGGTATAATGAGCTGTGACGTCCCCTCCAGGGATG
NDP52 C40A REV	CATCCCTGGAGGGGACGTCACAGCTCATTATACCTTCAC
NDP52 C108A FWD	CACCATCCTCATCCACATAGGCGAACTGGTAATACTCATCAT
NDP52 C108A REV	ATGATGAGTATTACCAGTTCGCCTATGTGGATGAGGATGGTG
NDP52 C153A FWD	CAGCTCCTGGTTTTCTTTGGCAAGCTCCTTGTTGTGCTGC
NDP52 C153A REV	GCAGCACAACAAGGAGCTTGCCAAAGAAAACCAGGAGCTG
NDP52 C163A FWD	CTTCTGGAGGCTGATAGCGCTGTCCTTCAGCTCC
NDP52 C163A REV	GGAGCTGAAGGACAGCGCTATCAGCCTCCAGAAG
NDP52 C321A FWD	GTCTCTGCAGAGCATTAGCTATAATTTCGTTCTCACTCAGTCTTTTTGA
NDP52 C321A REV	TCAAAAAGACTGAGTGAGAACGAAATTATAGCTAATGCTCTGCAGAGAC
NDP52 C397A FWD	CTTTGCAGATAGGGGCTTTCTTGATGGAGAGCGGGCT
NDP52 C397A REV	AGCCCGCTCTCCATCAAGAAAGCCCCTATCTGCAAAG
NDP52 C400A FWD	ATCACAAATATCATCTGCTTTGGCGATAGGGCATTTCTTGATGGAG
NDP52 C400A REV	CTCCATCAAGAAATGCCCTATCGCCAAAGCAGATGATATTTGTGAT
NDP52 C406A FWD	CCAAGGTGTGATCAGCAATATCATCTGCTTTGCAGATAGGGCA
NDP52 C406A REV	TGCCCTATCTGCAAAGCAGATGATATTGCTGATCACACCTTGG
NDP52 C419A FWD	CACAAATTGGACAATTGAAAGCAAGGGGCTGCATCTGCTGT
NDP52 C419A REV	ACAGCAGATGCAGCCCCTTGCTTTCAATTGTCCAATTTGTG

Appendix Table S3. Primers used to clone NDP52 and OPTN into pLENTI6/V5-DEST vector

Primer	Sequence (5'-3')
His-FLAG-pLENTI6 FWD	TCGAGTCTAGAGGGCCCGCGGTT
His-FLAG-pLENTI6 REV	T GCGGCCGCTGCCCATCTTGT
NDP52pLENTI6 FWD	ACAAGATGGGCAGCGGCCGCATGGAGGAGACCATCAAAGATCC
NDP52pLENTI6 REV	CGCGGGCCCTCTAGACTCGATCAGAGAGAGTGGCAGAACACG

Appendix Table S4. Primers used for NDP52 mutagenesis (Cys to Ser)

Primer	Sequence (5'-3')
NDP52 C18S FWD	CTGTCTTGCTGGATCACAGTCATTTCTCTCAGGTC
NDP52 C18S REV	GACCTGAGAGAAATGACTGTGATCCAGCAAGACAG
NDP52 C153S FWD	CCTGGTTTTCTTTGCTAAGCTCCTTGTTGTGCTGC
NDP52 C153S REV	GCAGCACAACAAGGAGCTTAGCAAAGAAAACCAGG
NDP52 C163S FWD	TCTGGAGGCTGATACTGCTGTCCTTCAGCTC
NDP52 C163S REV	GAGCTGAAGGACAGCAGTATCAGCCTCCAGA
NDP52 C321S FWD	CTCTGCAGAGCATTACTTATAATTTCGTTCTCACTCAGTCTTT
NDP52 C321S REV	AAAGACTGAGTGAGAACGAAATTATAAGTAATGCTCTGCAGAG
