# Supplementary material for: NDP52 acts as a redox sensor in PINK1/Parkin‐mediated mitophagy
Source: EMBO J. 2022 Dec 14;42(5):e111372. doi: 10.15252/embj.2022111372 (PMC9975939; doi:10.15252/embj.2022111372)
Supplement: Supplementary file 2 — Expanded View Figures PDF [file EMBJ-42-e111372-s005.pdf]

# Expanded View Figures

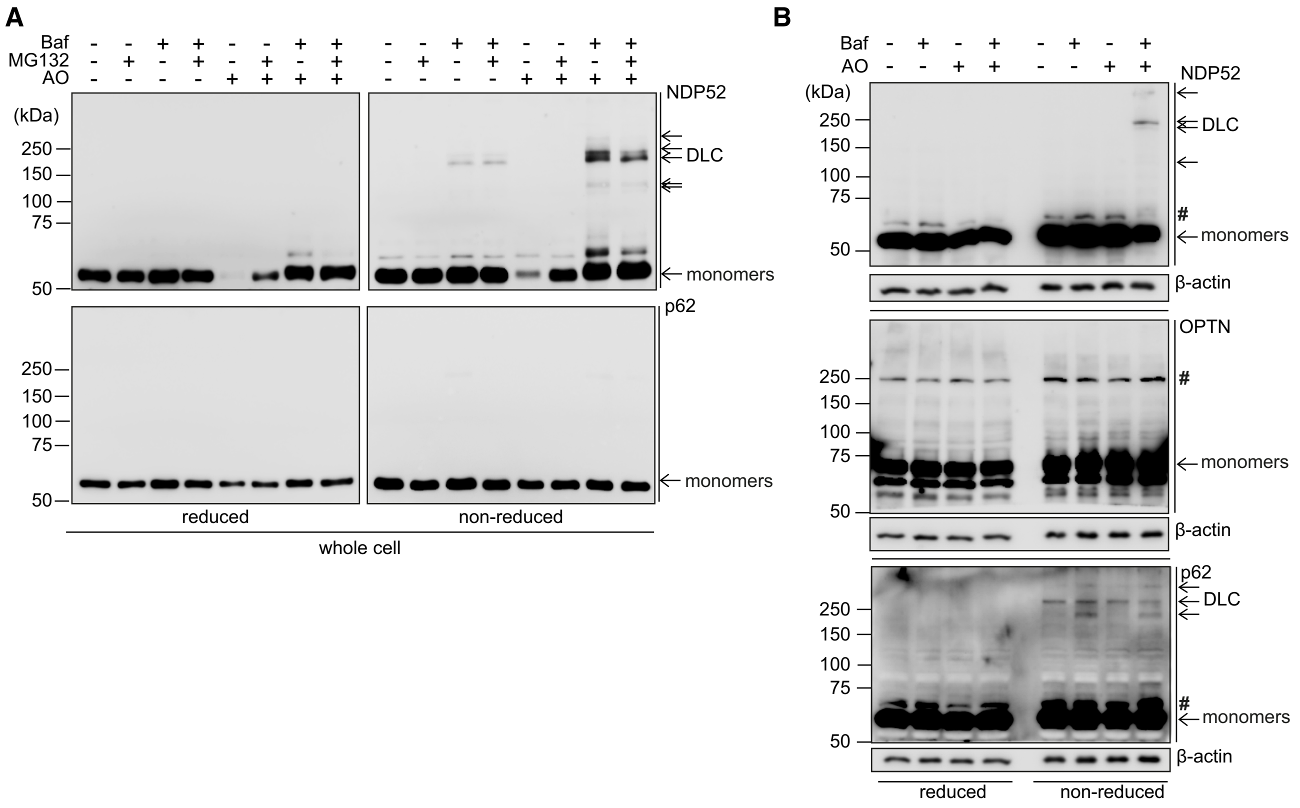


**Figure EV1. NDP52 but not p62 nor OPTN forms DLC upon mitochondrial damage.**

A, B HeLa cells were treated with 4 μM/10 μM AO in the presence or absence of 400 nM Baf (A, B) and 10 μM MG132 (A) for 3 h and analysed by immunoblotting for endogenous NDP52, p62 and OPTN in either reducing (2.5% β-mE) or non-reducing conditions. #, non-specific band or a post-translational modification.

Source data are available online for this figure.

**Figure EV2. Modelling of NDP52 tetramers and the property of NDP52 cysteine mutants.**

A Structural model of NDP52 dimer generated by AlphaFold2. Cys163 and Cys321 residues are both part of the coiled-coil interface and are orientated towards each other (within a distance of ~2.8 Å and ~1.3 Å, respectively). Cys153 is not part of the hydrophobic core and is orientated towards opposing sides of the homodimer.

B Structural models of a parallel or an antiparallel tetramer (dimer of dimers) of the NDP52 protein.

C Calculated root-mean-square deviation (RMSD) from atomistic molecular dynamics (MD) simulations for 100 ns of the NDP52 parallel and antiparallel tetramers fitted between the SKICH and CC domains.

D Calculated RMSD from coarse-grain MD simulations of the NDP52 antiparallel tetramer interacted between SKICH domains for 500 ns.

E Confocal images showing the recruitment of FIP200-GFP to glutathione Sepharose beads coated with GST-4xUbiquitin (Ub) and incubated with untagged wild type or mutant NDP52.

F Immunoblotting to analyse the expression level of NDP52 in HeLa WT, PentaKO, PentaKO + NDP52 WT and PentaKO + NDP52 Mut cells stably expressing YFP-Parkin and mt-mKeima.

G Recombinant NDP52 WT and NDP52 Mut (2 ng) proteins, and cell lysates (30 μg) of HeLa PentaKO cells stably expressing NDP52 WT or NDP52 Mut, were subjected to NATIVE-PAGE followed by immunoblotting analyses.

H Fluorescence images and quantification of mitophagy in HeLa PentaKO cells stably expressing YFP-Parkin and mt-mKeima, transiently transfected with the indicated NDP52 constructs and treated with AO for 2 h.

Data information: Data are mean ± s.e.m. or displayed as cell popular violin plots (D, G). *P* values were calculated by unpaired two-tailed Student's *t*-test (D) or one-way ANOVA followed by Sidak test (G) on three independent experiments. *, $P < 0.05$; **, $P < 0.01$; ***, $P < 0.001$; ns (non-significant). Scale bars: 50 μm (D); 20 μm (G). Source data are available online for this figure.

© 2022 The Authors

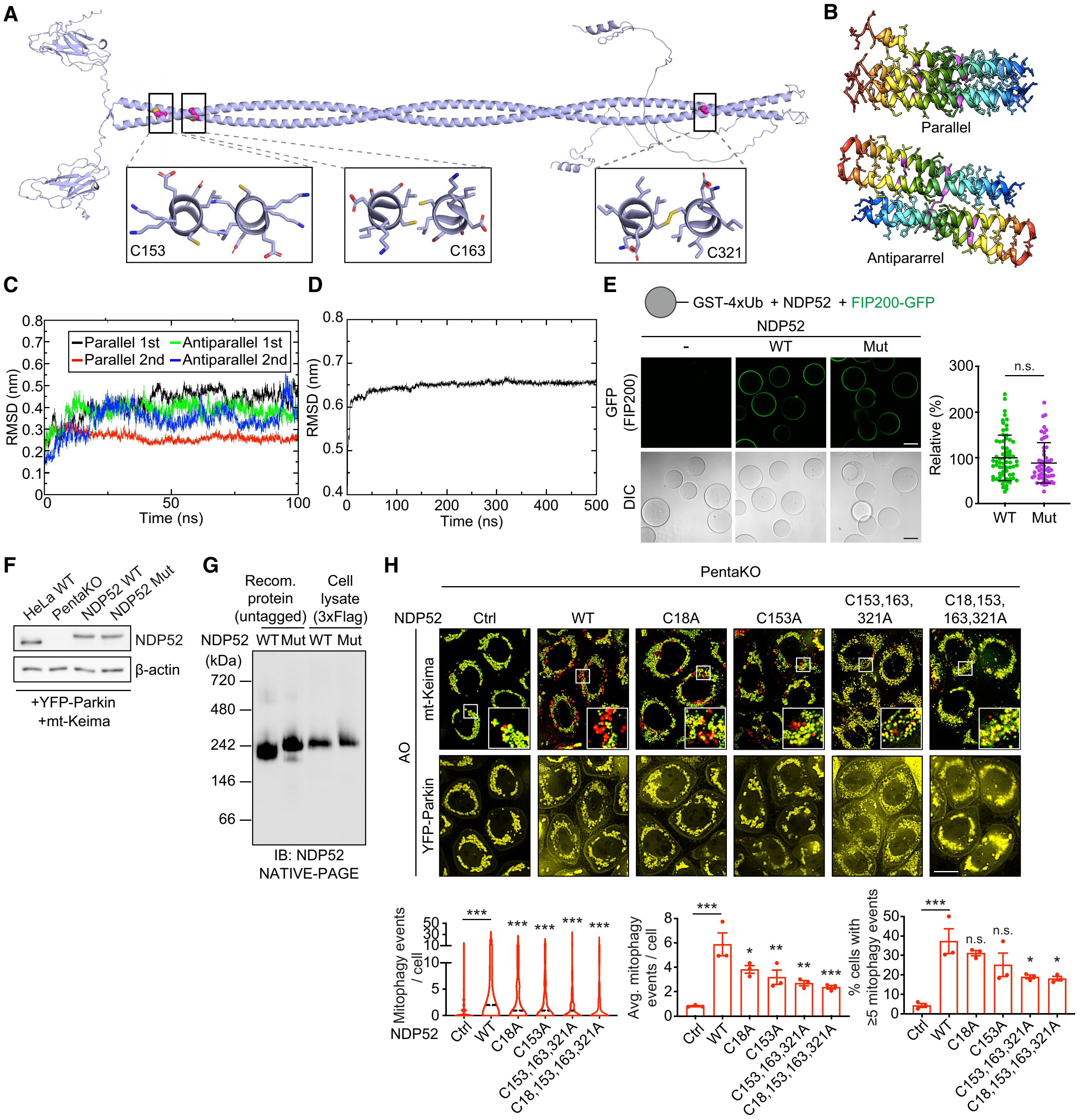


Figure EV2.

 © 2022 The Authors

**Figure EV3. Oxidation of NDP52 facilitates PINK1/Parkin mitophagy with no role in mitophagy triggered by iron chelation.**

A Fluorescence images of YFP-Parkin in HeLa WT, PentaKO, PentaKO + NDP52 WT and PentaKO + NDP52 Mut cells stably expressing YFP-Parkin and mt-mKeima, in the same fields and conditions as Fig 4A.

B Fluorescence microscopy images and quantification of mitophagy of HeLa WT, PentaKO, PentaKO + NDP52 WT and PentaKO + NDP52 Mut cells stably expressing YFP-Parkin and mt-mKeima were treated with 1 mM DFP for 24 h.

C Fluorescence microscopy images and quantification of mitophagy of HeLa WT, PentaKO, PentaKO + NDP52 WT and PentaKO + NDP52 Mut cells stably expressing YFP-Parkin and mt-mKeima, pre-treated with or without 500 nM MitoQ for 16 h and treated with 10 μM G-TPP for 8 h.

D–F HeLa PentaKO + NDP52 WT cells were pre-treated with or without MitoQ for 16 h and treated with G-TPP for 5 h or AO for 2 h, followed by immunoblotting for NDP52 in non-reducing conditions (D), MitoSOX staining (E) and mitochondrial membrane potential assay by using TMRM and Mitotracker Green (MTG) staining (F).

Data information: Data are mean ± s.e.m. (B, C, E, F) or displayed as cell popular violin plots (C). *P* values were calculated by one-way ANOVA followed by Sidak test on three independent experiments (B, C, E, F). *, $P < 0.05$; **, $P < 0.01$; ***, $P < 0.001$; §§, $P < 0.01$; §§§, $P < 0.001$ (relative to MitoQ-untreated condition); ns (non-significant). Scale bars: 20 μm (A, B, C, E, F).
Source data are available online for this figure.

© 2022 The Authors 

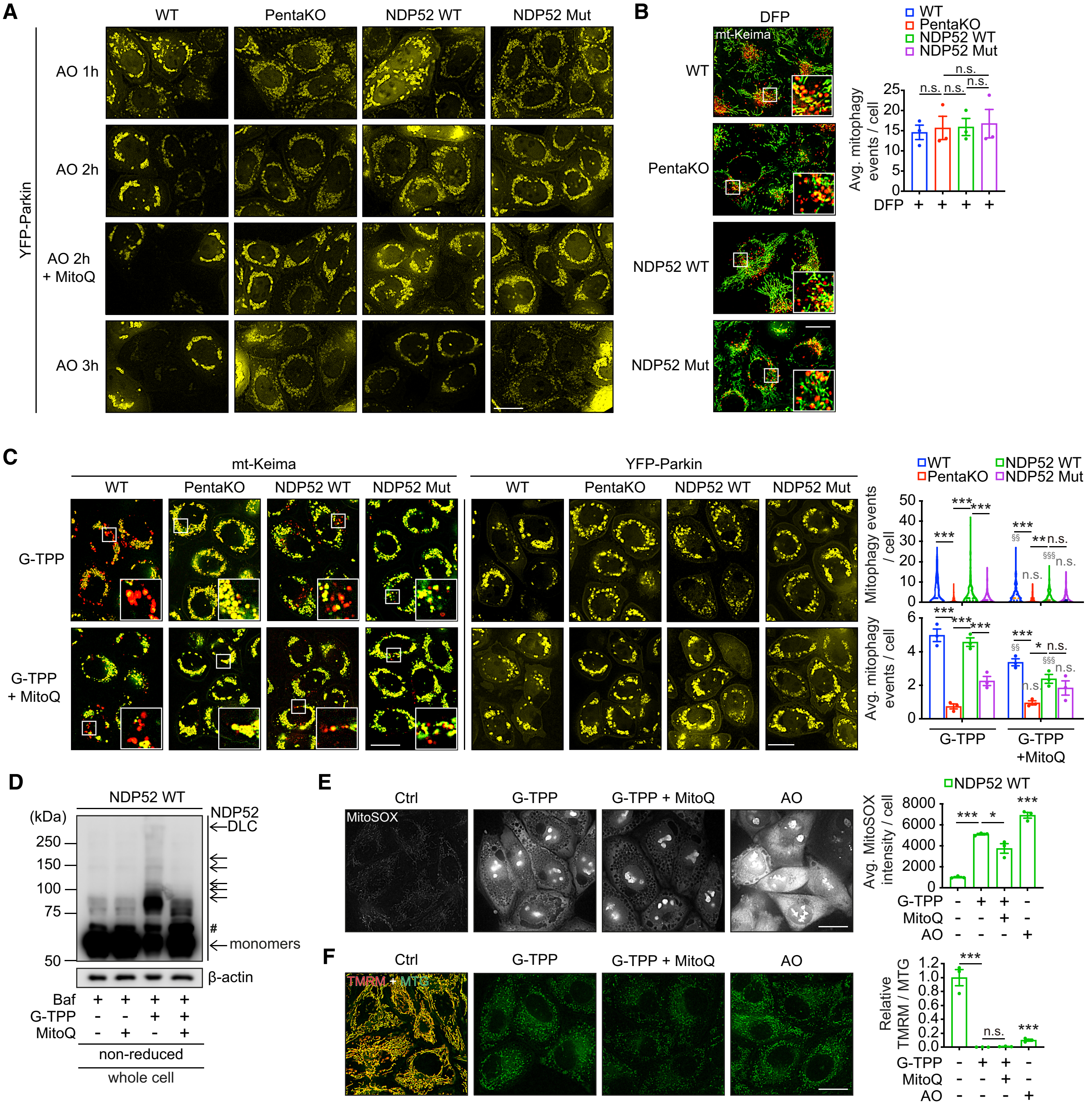


Figure EV3.

 © 2022 The Authors

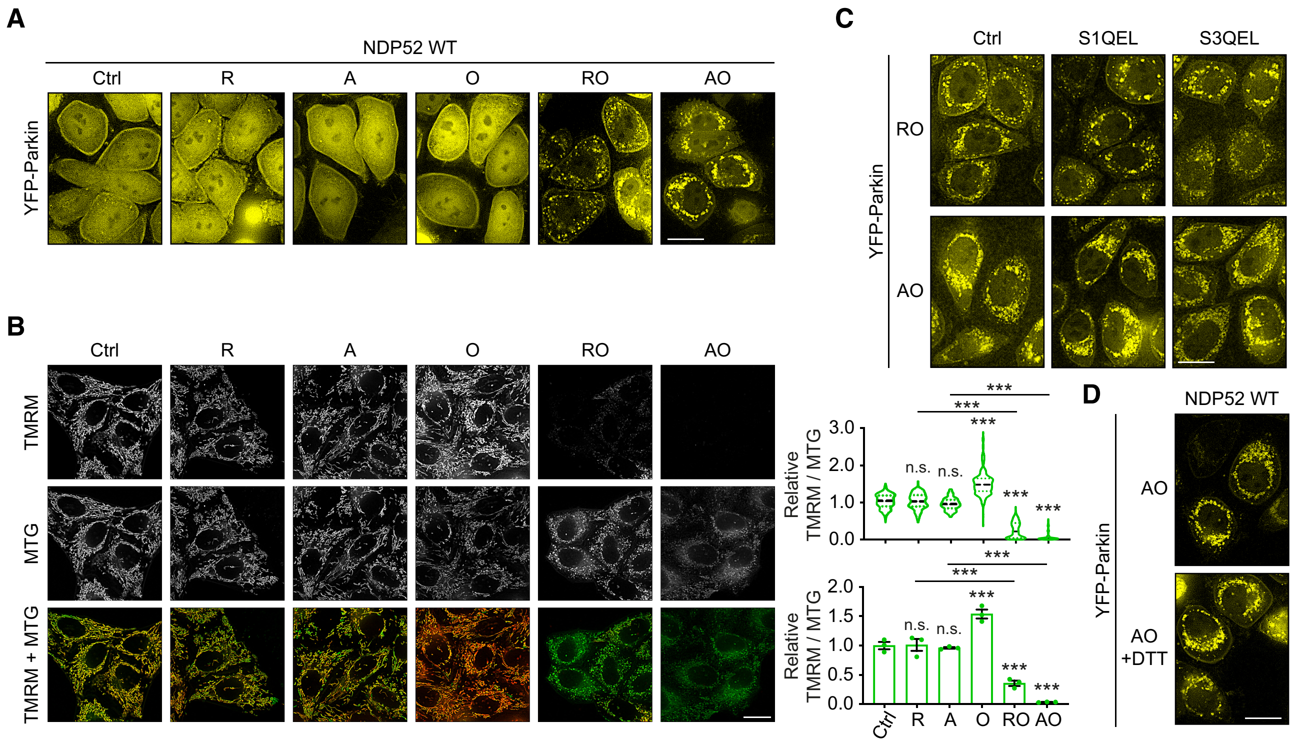


**Figure EV4. Recruitment of Parkin and mitochondrial membrane potential upon mitochondrial damage induced by mitochondrial inhibitors.**

A Fluorescence images of YFP-Parkin in HeLa PentaKO + NDP52 WT cells stably expressing YFP-Parkin and mt-mKeima, in the same fields and conditions as Fig 5A.

B Fluorescence images of HeLa PentaKO + NDP52 WT cells treated with 4 μM R, 4 μM A, 10 μM O, or the combination of RO or AO for 3 h, followed by mitochondrial membrane potential assay by using TMRM and MTG staining.

C, D Fluorescence images of YFP-Parkin in HeLa PentaKO + NDP52 WT cells stably expressing YFP-Parkin and mt-mKeima, in the same fields and conditions as Fig 5C and F, respectively.

Data information: Data are mean ± s.e.m. or displayed as cell popular violin plots (B). *P* values were calculated by one-way ANOVA followed by Sidak test on three independent experiments (B). ***, $P < 0.001$; ns (non-significant). Scale bars: 20 μm (A–D).

**Figure EV5. Recruitment of autophagy/mitophagy proteins to mitochondria in HeLa cells and MEFs.**

A HeLa PentaKO + NDP52 WT and PentaKO + NDP52 Mut cells stably expressing YFP-Parkin and mt-mKeima, in the same conditions as Fig 6B and C, followed by immunoblotting for the indicated proteins in whole cell lysate, cytoplasmic and mitochondrial fractions in reducing conditions. Note that the LC3, NDP52 and actin blots of whole cell lysate are from the same membranes as those shown in Fig 6C.

B HeLa PentaKO + NDP52 WT or NDP52 Mut cells stably expressing YFP-Parkin and mt-mKeima were treated with AO for 3 h in the presence or absence of 400 nM Baf, followed by immunofluorescence analyses.

C, D MEFs stably expressing YFP-Parkin, mt-mKeima and empty (Flag), Flag-human NDP52 WT (hNDP52) or Flag-hNDP52 Mut were analysed by immunoblotting for the expression level of hNDP52 (C) and DLC formation upon AO/Baf treatment for 3 h (D) in reducing (C, 2.5% β-mE) or non-reducing (D) conditions.

E MEFs stably expressing YFP-Parkin, mt-mKeima and empty (Flag), Flag-hNDP52 WT or Flag-hNDP52 Mut were treated with AO and Baf for 3 h followed by immunofluorescence analyses.

F Fluorescence images of YFP-Parkin in MEFs stably expressing YFP-Parkin, mt-mKeima and empty (Flag), Flag-hNDP52 WT or Flag-hNDP52 Mut, in the same fields and conditions as Fig 6E.

Data information: The number of foci of the indicated proteins colocalised with NDP52, or foci of NDP52 colocalised with Parkin, was quantified (B, E). Data are mean ± s.e.m. (B, E). *P* values were calculated by unpaired two-tailed Student's *t*-test (B) or one-way ANOVA followed by Sidak test (E) on three independent experiments. ***, $P < 0.001$ (relative to MEFs transduced with an empty vector); ns (non-significant). Scale bars: 20 μm (B, E, F).
Source data are available online for this figure.

© 2022 The Authors

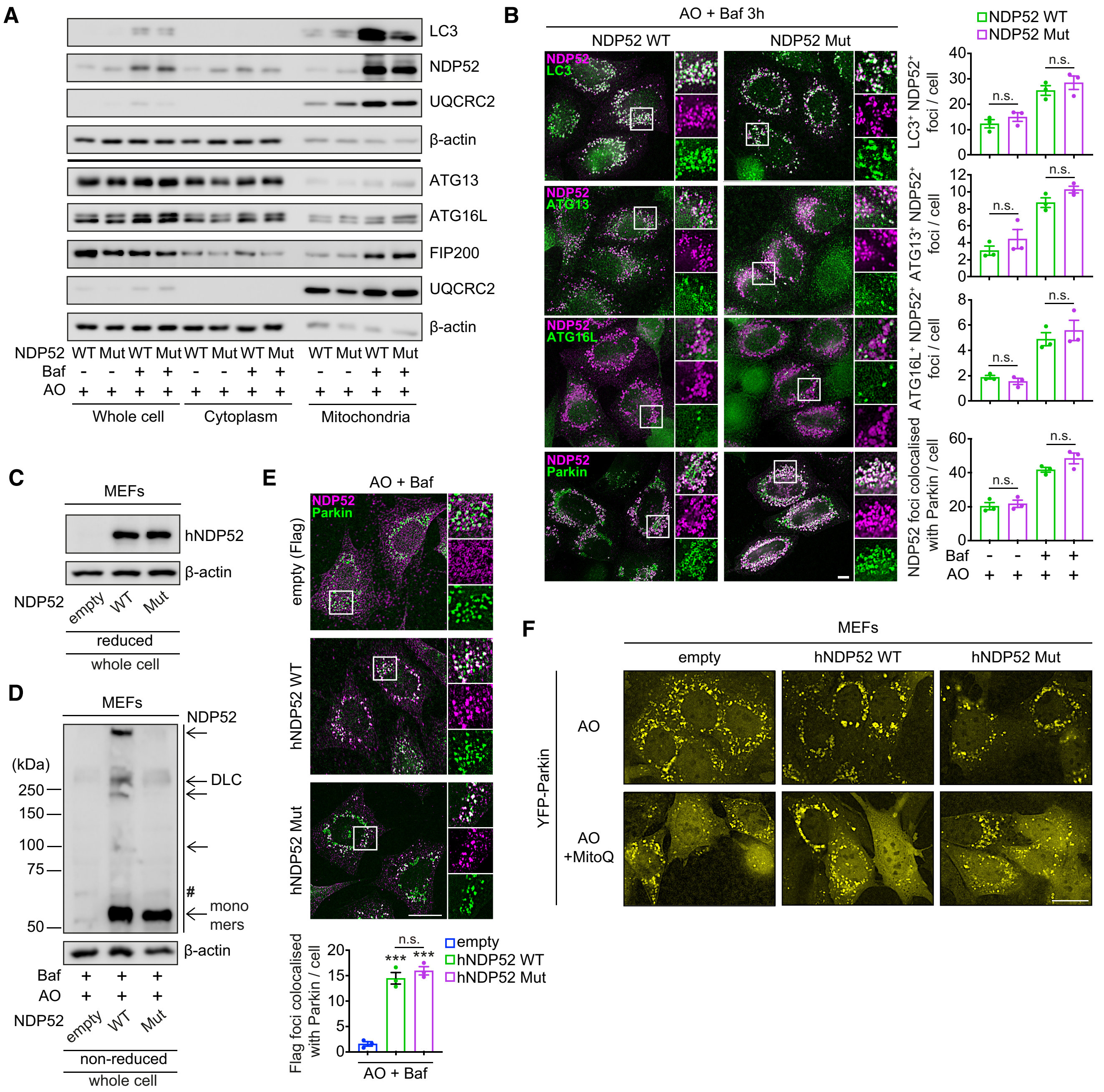


**Figure EV5.**

 © 2022 The Authors
