# Supplementary figures and images for: NDP52 acts as a redox sensor in PINK1/Parkin‐mediated mitophagy
Source: EMBO J. 2022 Dec 14;42(5):e111372. doi: 10.15252/embj.2022111372 (PMC9975939; doi:10.15252/embj.2022111372)

**Fig 1A**

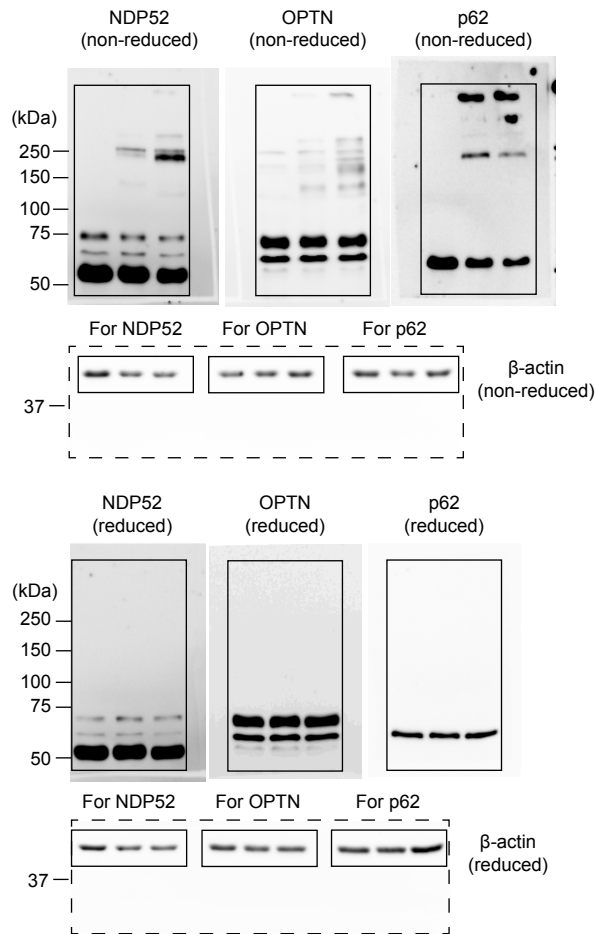

**Fig 1B**

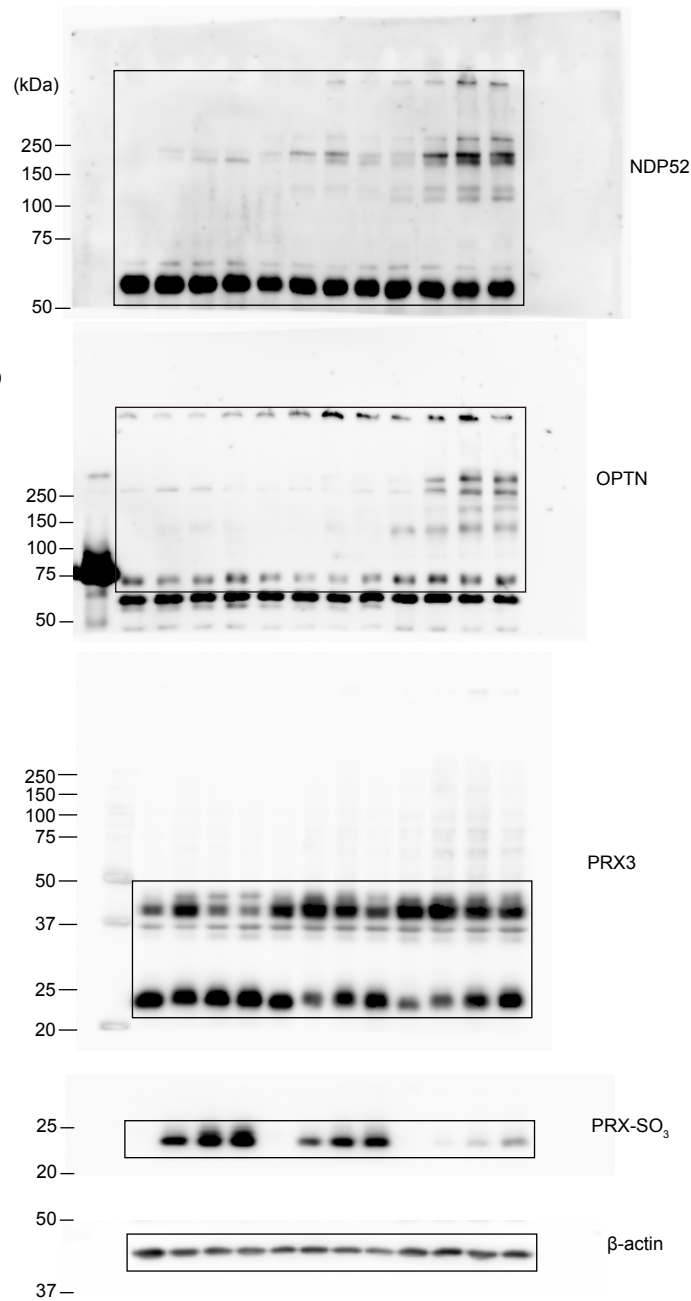

**Fig 1C**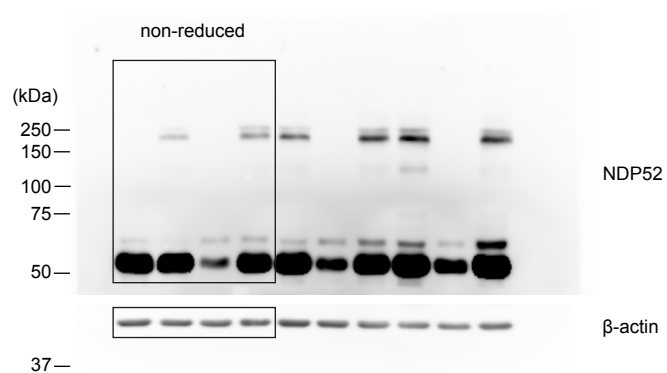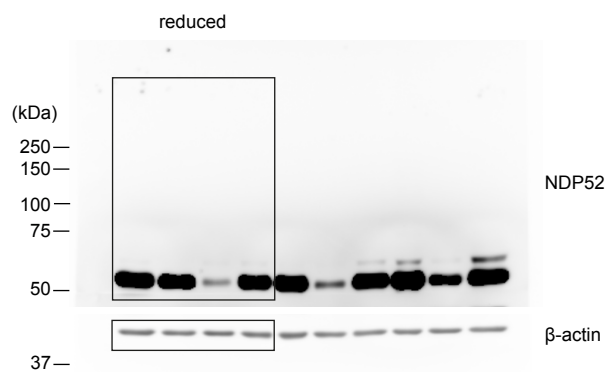**Fig 1D**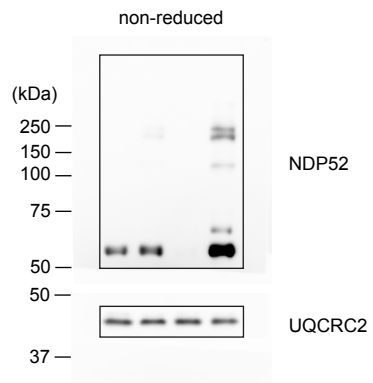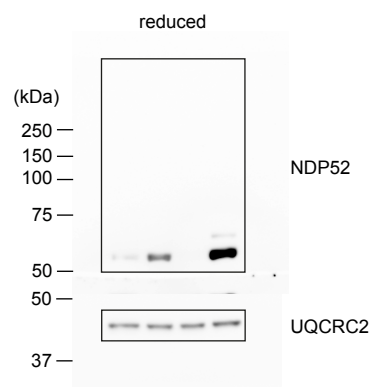

Supplement: Supplementary file 5 — Source Data for Figure 1 [file EMBJ-42-e111372-s003.zip › EMBOJ-2022-111372_SourceDataForFigure1.pdf]

**Fig 2A**

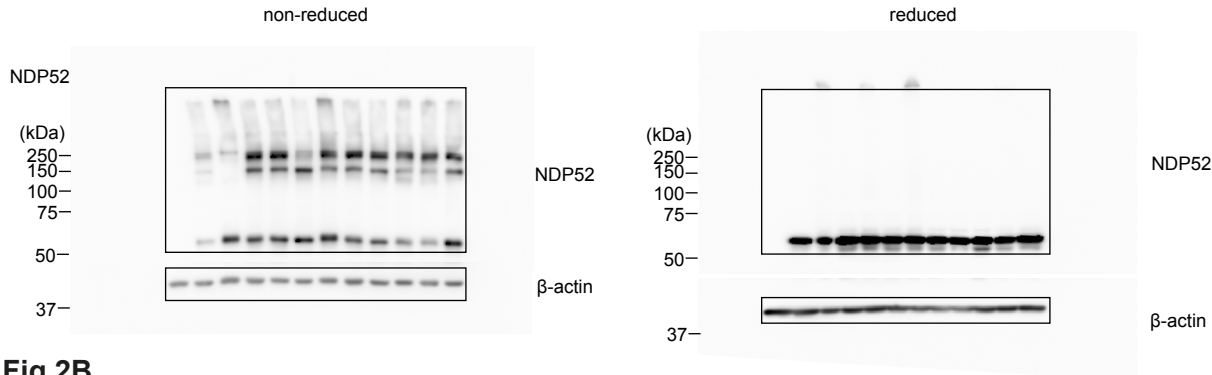

**Fig 2B**

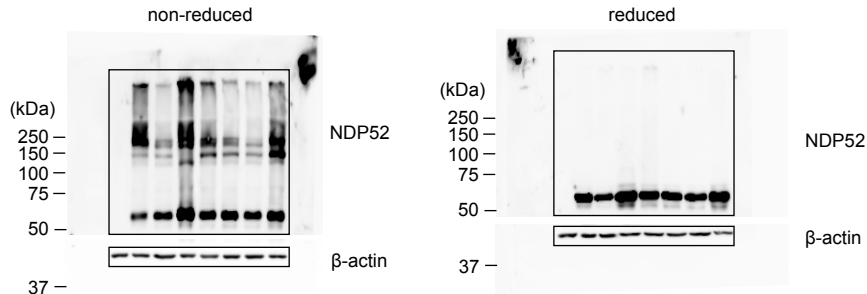

**Fig 2C**

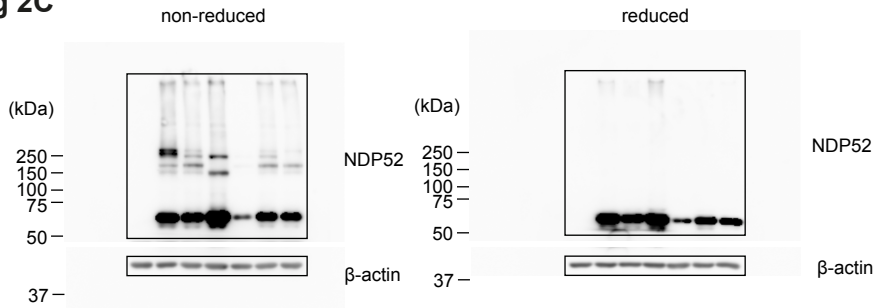

**Fig 2D**

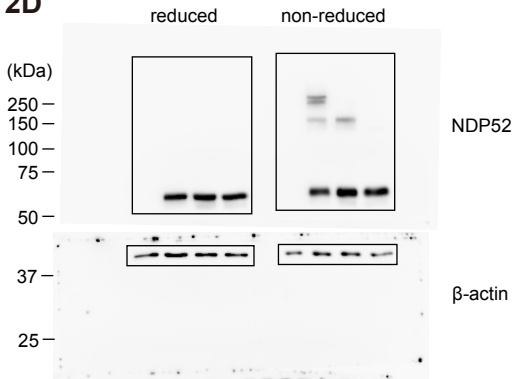

Supplement: Supplementary file 6 — Source Data for Figure 2 [file EMBJ-42-e111372-s006.zip › EMBOJ-2022-111372_SourceDataForFigure2.pdf]

**Fig 3B**

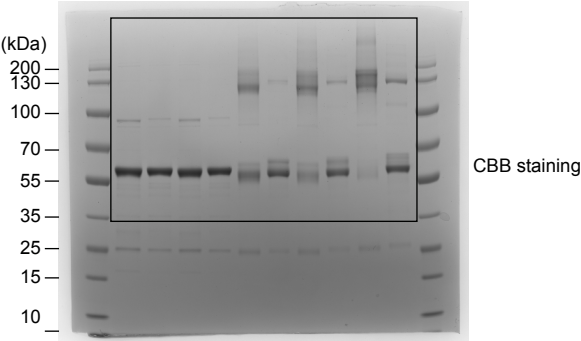

**Fig 3C**

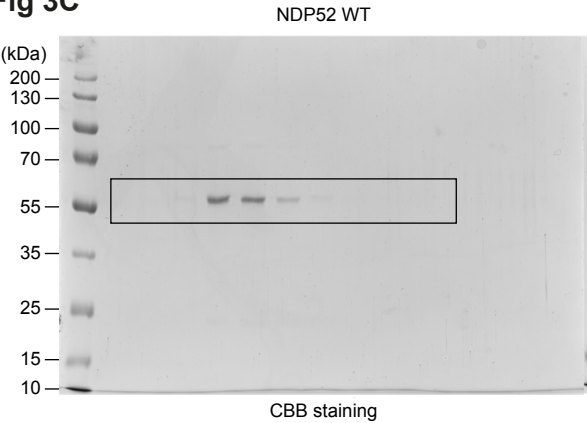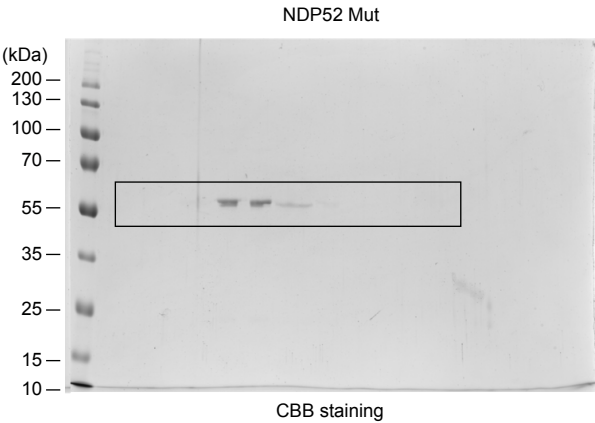

**Fig 3D**

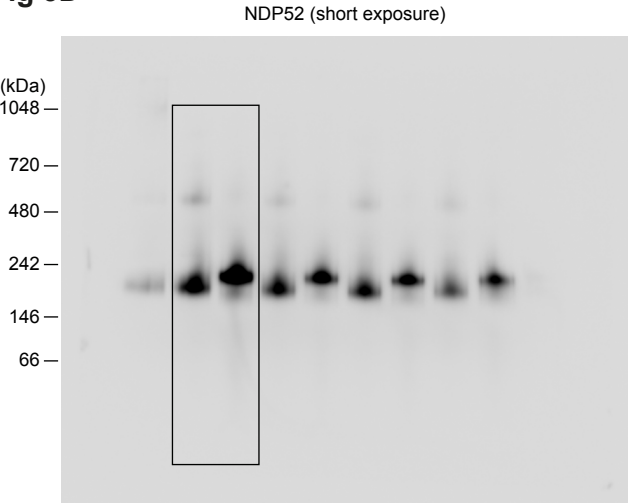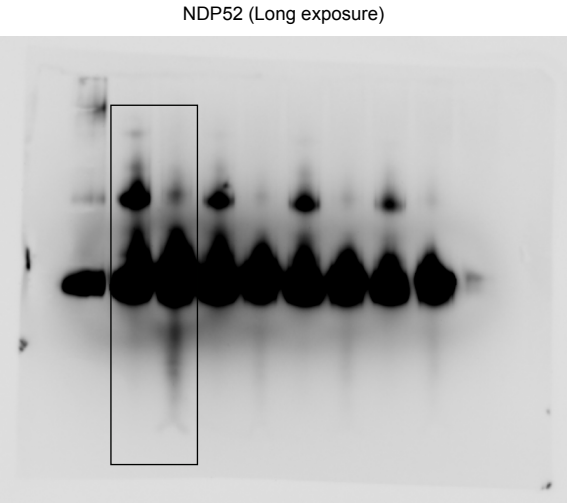

Supplement: Supplementary file 7 — Source Data for Figure 3 [file EMBJ-42-e111372-s011.zip › EMBOJ-2022-111372_SourceDataForFigure3.pdf]

Fig 4C

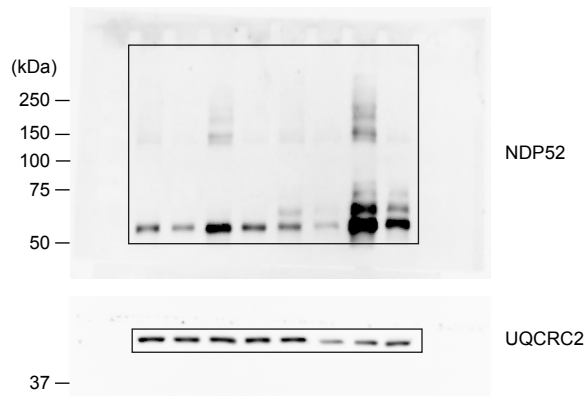

Fig 4E

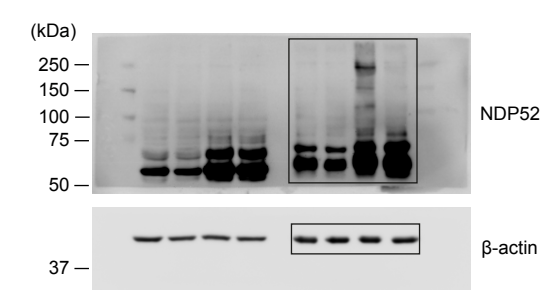

Supplement: Supplementary file 8 — Source Data for Figure 4 [file EMBJ-42-e111372-s004.zip › EMBOJ-2022-111372_SourceDataForFigure4.pdf]

**Fig 5E**

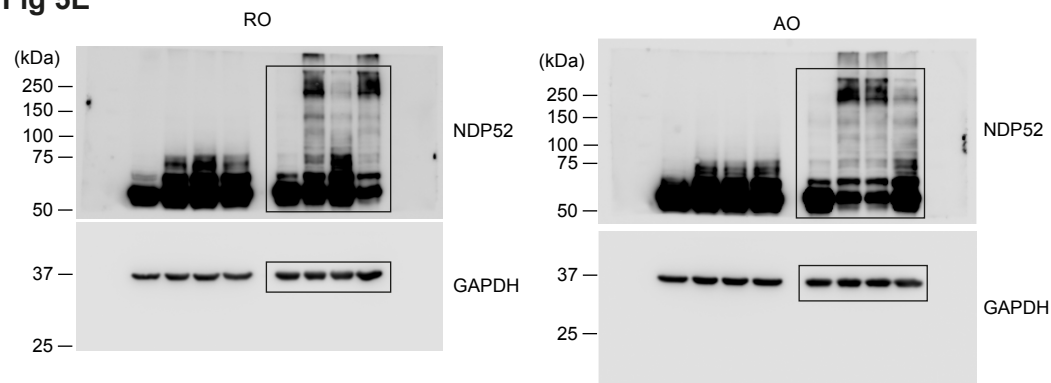

**Fig 5G**

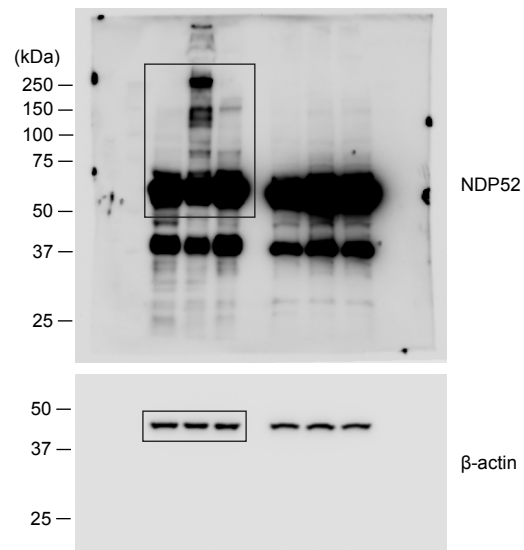

Supplement: Supplementary file 9 — Source Data for Figure 5 [file EMBJ-42-e111372-s009.zip › EMBOJ-2022-111372_SourceDataForFigure5.pdf]

**Fig 6B**

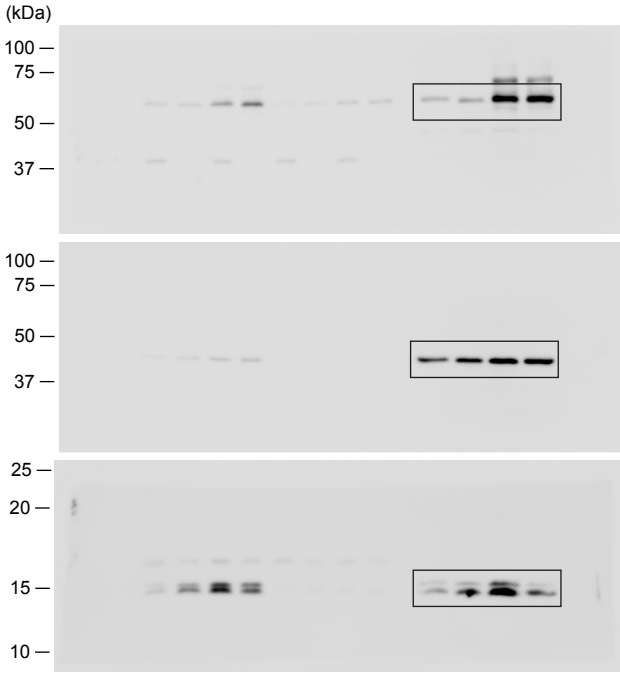

**Fig 6D**

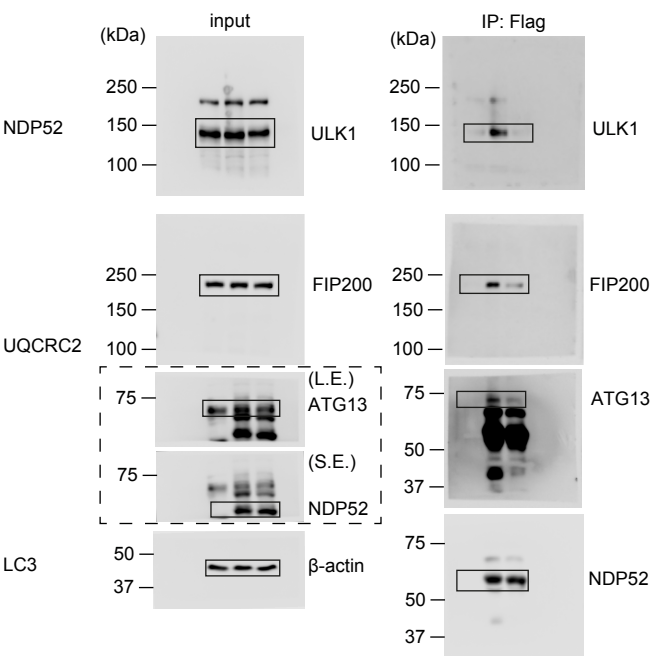

**Fig 6C**

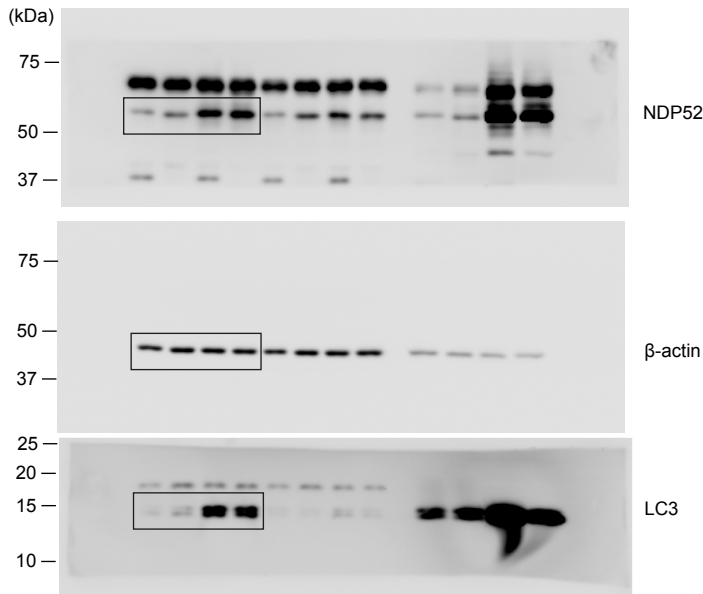

Supplement: Supplementary file 10 — Source Data for Figure 6 [file EMBJ-42-e111372-s010.zip › EMBOJ-2022-111372_SourceDataForFigure6.pdf]
