# Supplementary material for: NDP52 acts as a redox sensor in PINK1/Parkin‐mediated mitophagy
Source: EMBO J. 2022 Dec 14;42(5):e111372. doi: 10.15252/embj.2022111372 (PMC9975939; doi:10.15252/embj.2022111372)
Supplement: Supplementary file 11 [file EMBJ-42-e111372-s007.pdf]

THE EMBO JOURNAL

NDP52 acts as a redox sensor in PINK1/Parkin-mediated mitophagy

Tetsushi Kataura, Elsje Otten, Yoana Rabanal Ruiz, Elias Adriaenssens, Francesca Urselli, Filippo Scialo, Lanyu Fan, Graham Smith, William Dawson, Xingxiang Chen, Wyatt Yue, Agnieszka Bronowska, Bernadette Carroll, Sascha Martens, Michael Lazarou, and Viktor Korolchuk
DOI: 10.15252/embj.2022111372

Corresponding author(s): Viktor Korolchuk (viktor.korolchuk@ncl.ac.uk)

Review Timeline:

Submission Date:	6th Apr 22
Editorial Decision:	6th May 22
Revision Received:	21st Sep 22
Editorial Decision:	2nd Nov 22
Revision Received:	9th Nov 22
Accepted:	11th Nov 22

Editor: William Teale

Transaction Report:

(Note: With the exception of the correction of typographical or spelling errors that could be a source of ambiguity, letters and reports are not edited. Depending on transfer agreements, referee reports obtained elsewhere may or may not be included in this compilation. Referee reports are anonymous unless the Referee chooses to sign their reports.)

1st Editorial Decision **6th May 2022**

Dear Dr Korolchuk,

Thank you again for the submission of your manuscript entitled "NDP52 acts as a redox sensor in PINK1/Parkin-mediated mitophagy" to EMBO Journal. I have now received the referees' reports, which are copied to the bottom of this message.

I would like to invite you to address the referees' comments in a revised version of the manuscript. However, I should make clear that I fully agree with the referees that two aspects of the manuscript need to be addressed. Firstly, the data which test your hypothesis that NDP52 oligomerization drives mitophagy in oxidizing environments need to be consolidated with a series of supporting experiments. Here, reviewer 2 provides a list of points to be addressed. Secondly, and as reviewers 1 states, your hypothesis needs to be extended to address how the oligomerization of NDP52 drives the induction of mitophagy. I agree with reviewer 3 here that this needs to be addressed experimentally and not just discussed (as reviewer 1 requests). If this extension into a more mechanistic study proves unfeasible, please let me know; we might then discuss the possibility of transferring the manuscript to EMBO Reports (though I have not yet broached the matter with the editorial team there). In any case, I think it would be a good idea to schedule a Zoom meeting for next week to talk this over. Please let me know when would be a good time.

I should add that it is The EMBO Journal policy to allow only a single major round of revision and that it is therefore important to resolve these concerns at this stage. I believe the concerns of the referees are reasonable and addressable, but please contact me if you have any questions, need further input on the referee comments or if you anticipate any problems in addressing any of their points. Please, follow the instructions below when preparing your manuscript for resubmission.

I would also like to point out that as a matter of policy, competing manuscripts published during this period will not be taken into consideration in our assessment of the novelty presented by your study ("scooping" protection). We have extended this 'scooping protection policy' beyond the usual 3 month revision timeline to cover the period required for a full revision to address the essential experimental issues. Please contact me if you see a paper with related content published elsewhere to discuss the appropriate course of action.

When preparing your letter of response to the referees' comments, please bear in mind that this will form part of the Review Process File, and will therefore be available online to the community. For more details on our Transparent Editorial Process, please visit our website: https://www.embopress.org/page/journal/14602075/authorguide#transparentprocess

Again, please contact me at any time during revision if you need any help or have further questions.

Thank you very much again for the opportunity to consider your work for publication. I look forward to your revision.

Best regards,

William

William Teale, Ph.D.
Editor
The EMBO Journal

When submitting your revised manuscript, please carefully review the instructions below and include the following items:

1) a .docx formatted version of the manuscript text (including legends for main figures, EV figures and tables). Please make sure that the changes are highlighted to be clearly visible.

2) individual production quality figure files as .eps, .tif, .jpg (one file per figure).

3) a .docx formatted letter INCLUDING the reviewers' reports and your detailed point-by-point response to their comments. As part of the EMBO Press transparent editorial process, the point-by-point response is part of the Review Process File (RPF), which will be published alongside your paper.

4) a complete author checklist, which you can download from our author guidelines (https://wol-prod-cdn.literatumonline.com/pb-assets/embo-site/Author Checklist%20-%20EMBO%20J-1561436015657.xlsx). Please insert information in the checklist that is also reflected in the manuscript. The completed author checklist will also be part of the RPF.

5) Please note that all corresponding authors are required to supply an ORCID ID for their name upon submission of a revised manuscript.

6) We require a 'Data Availability' section after the Materials and Methods. Before submitting your revision, primary datasets produced in this study need to be deposited in an appropriate public database, and the accession numbers and database listed under 'Data Availability'. Please remember to provide a reviewer password if the datasets are not yet public (see https://www.embopress.org/page/journal/14602075/authorguide#datadeposition). If no data deposition in external databases is needed for this paper, please then state in this section: This study includes no data deposited in external repositories. Note that the Data Availability Section is restricted to new primary data that are part of this study.

Note - All links should resolve to a page where the data can be accessed.

7) When assembling figures, please refer to our figure preparation guideline in order to ensure proper formatting and readability in print as well as on screen:
http://bit.ly/EMBOPressFigurePreparationGuideline

Please remember: Digital image enhancement is acceptable practice, as long as it accurately represents the original data and conforms to community standards. If a figure has been subjected to significant electronic manipulation, this must be noted in the figure legend or in the 'Materials and Methods' section. The editors reserve the right to request original versions of figures and the original images that were used to assemble the figure.

8) For data quantification: please specify the name of the statistical test used to generate error bars and P values, the number (n) of independent experiments (specify technical or biological replicates) underlying each data point and the test used to calculate p-values in each figure legend. The figure legends should contain a basic description of n, P and the test applied. Graphs must include a description of the bars and the error bars (s.d., s.e.m.).

9) We would also encourage you to include the source data for figure panels that show essential data. Numerical data can be provided as individual .xls or .csv files (including a tab describing the data). For 'blots' or microscopy, uncropped images should be submitted (using a zip archive or a single pdf per main figure if multiple images need to be supplied for one panel). Additional information on source data and instruction on how to label the files are available at .

10) We replaced Supplementary Information with Expanded View (EV) Figures and Tables that are collapsible/expandable online (see examples in https://www.embopress.org/doi/10.15252/embj.201695874). A maximum of 5 EV Figures can be typeset. EV Figures should be cited as 'Figure EV1, Figure EV2" etc. in the text and their respective legends should be included in the main text after the legends of regular figures.

- For the figures that you do NOT wish to display as Expanded View figures, they should be bundled together with their legends in a single PDF file called *Appendix*, which should start with a short Table of Content. Appendix figures should be referred to in the main text as: "Appendix Figure S1, Appendix Figure S2" etc. See detailed instructions regarding expanded view here: .

- Additional Tables/Datasets should be labelled and referred to as Table EV1, Dataset EV1, etc. Legends have to be provided in a separate tab in case of .xls files. Alternatively, the legend can be supplied as a separate text file (README) and zipped together with the Table/Dataset file.

11) Our journal encourages inclusion of *data citations in the reference list* to directly cite datasets that were re-used and obtained from public databases. Data citations in the article text are distinct from normal bibliographical citations and should directly link to the database records from which the data can be accessed. In the main text, data citations are formatted as follows: "Data ref: Smith et al, 2001" or "Data ref: NCBI Sequence Read Archive PRJNA342805, 2017". In the Reference list, data citations must be labeled with "[DATASET]". A data reference must provide the database name, accession number/identifiers and a resolvable link to the landing page from which the data can be accessed at the end of the reference. Further instructions are available at .

Additional instructions for preparing your revised manuscript:

Please make sure you upload a letter of response to the referees' comments together with the revised manuscript.

Please also check that the title and abstract of the manuscript are brief, yet explicit, even to non-specialists.

When assembling figures, please refer to our figure preparation guideline in order to ensure proper formatting and readability in print as well as on screen:
https://bit.ly/EMBOPressFigurePreparationGuideline
See also guidelines for figure legends: https://www.embopress.org/page/journal/14602075/authorguide#figureformat

IMPORTANT: When you send the revision we will require
- a point-by-point response to the referees' comments, with a detailed description of the changes made (as a word file).

- a word file of the manuscript text.
- individual production quality figure files (one file per figure)
- a complete author checklist, which you can download from our author guidelines (https://www.embopress.org/page/journal/14602075/authorguide).
- Expanded View files (replacing Supplementary Information)
Please see out instructions to authors
https://www.embopress.org/page/journal/14602075/authorguide#expandedview

Please remember: Digital image enhancement is acceptable practice, as long as it accurately represents the original data and conforms to community standards. If a figure has been subjected to significant electronic manipulation, this must be noted in the figure legend or in the 'Materials and Methods' section. The editors reserve the right to request original versions of figures and the original images that were used to assemble the figure.

Further information is available in our Guide For Authors: https://www.embopress.org/page/journal/14602075/authorguide

We realize that it is difficult to revise to a specific deadline. In the interest of protecting the conceptual advance provided by the work, we recommend a revision within 3 months (4th Aug 2022). Please discuss the revision progress ahead of this time with the editor if you require more time to complete the revisions. Use the link below to submit your revision:

https://emboj.msubmit.net/cgi-bin/main.plex

Referee #1:

Mitophagy is an important process to remove damaged mitochondria. It has been proposed that mitochondrial dysfunction is also associated with an increased generation of reactive oxygen species and it is thus tempting to speculate that increased ROS generation can modulate mitophagy. In the present manuscript, Kataura and colleagues propose that one such mechanism is ROS-dependent cysteine oxidation of NDP52 and the formation of oligomers that facilitates recruitment of the autophagy machinery.
This is a nice technically well executed study that presents the formation of ROS-mediated formation of NDP52 oligomers upon oxidative insult and harsh conditions used to induce mitophagy, identifies the involved cysteines and shows that mutation of these cysteines hampers mitophagy.
I have a few major points that should be addressed to improve the manuscript.

1/ the treatments applied to induce NDP52 oligomerisation are quite harsh (I am aware of the fact that these conditions are often used for mechanistic studies of mitophagy). I miss experiments employing physiologically more meaningful (i.e. milder) conditions to demonstrate a physiological significance of the disulphide-dependent oligomerisation and the link to mitophagy

2/ Is the induction of disulphide bond-dependent oligomerization reversible, i.e. can addition of e.g. DTT to cells reverse it and stop mitophagy induction

3/ Mutation of multiple cysteines in NDP52 might impair its folding. How can the authors exclude that it is not misfolding that hampers initiation of mitophagy instead of the proposed redox transition?

Minor Points
1/ I think it is a bit unfortunate that reducing and non-reducing conditions are always presented on separate gels/blots. It is hard to estimate amounts and shifts between different species of NDP52

2/ why is oligomerization important for induction of mitophagy? The underlying mechanism could be more extensively discussed.

Referee #2:

In this report format paper the Korolchuk group follows up on their original discovery on redox regulation of oligomerization of p62 (Nat Commun. 2018 Jan 17;9(1):256. doi: 10.1038/s41467-017-02746-z. PMID: 29343728), but this time turning their attention to another soluble autophagy receptor, NDP52 (CALCOCO2), and its role in mitophagy. Damaged mitochondria produce ROS (reactive oxygen species) but the mechanism(s) on how this is sensed to induce mitophagy is/are not clarified. Here, Kataura et al. present data to suggest that NDP52 has several redox sensitive cysteines that form disulphide bonds to

oligomerize NDP52 in the presence of ROS. Mitochondria-derived ROS can induce oligomerization of NDP52 on the mitochondria, due to formation of disulphide-linked conjugates (DLC), to facilitate recruitment of the basal autophagy apparatus for effective induction of mitophagy.

The model suggested here is interesting, but there is not enough evidence in the present version of this paper to properly support the conclusions made. Also, the impact the 4Cys mutant of NDP52 has on the induced mitophagy is on the kinetics and it is not a strong effect that reduces strongly the mitophagy observed.

Some specific major points:
1. The evidence for ROS-induced oligomerization rests on only one assay method, gel electrophoresis under non-reduced conditions. It is important to also use gel filtration/SEC-MALS (see below) to test this. The data are presented from experiments done only on HeLa cells. No neuronal cell model or murine (or other animal) models are used to try to test the in vivo relevance of the disulphide-linked conjugates (DLC) formation in PINK1-PARKIN-dependent mitophagy. These are important weaknesses of this study that reduces the impact of the findings presented here.
2. It has been shown previously by size-exclusion chromatography with multi-angle light scattering (SEC-MALS) that NDP52 behaves as a dimer in solution mediated by its central coiled-coil (CC) domain (Nat Commun 2013;4:1613. doi: 10.1038/ncomms2606). Further work by the same group showed the CC domain of NDP52 to form a parallel dimer (Sci. Rep. 7:43318. DOI: 10.1038/srep43318). This means that NDP52 is most likely already a dimer when recruited to mitochondria. In the present paper no attempt has been done to relate the ROS induced DLC formation, and the Cys residues implicated by mutations to be responsible, to the structure of NDP52. The apparent MW from gel electrophoresis and WB detection shown in Fig 1 suggest that a trimer of NDP52 may be formed. This is not discussed or mentioned at all by the authors. How does this relate to NDP52 being a parallel dimer, or is it a dimer we primarily see in the non-reduced gels? The authors need to provide some data to explore this.
3. The four Cys residues being important for DLC formation are located in the SKICH domain (C18) and in the large central CC domain (C153, 163 and 321) and as such spaced widely apart. It is difficult to understand why a Cys residue in the SKICH domain should be required.
Can the authors rationalize this in any way?
4. As pointed out by the authors, all the four Cys residues implicated by mutations in the formation of DLC are not evolutionary conserved outside primates. Even the Rhesus monkey does not have all four of them conserved. This speaks against a major role. The authors argue that this is a mechanism evolved to tackle oxidative stress and is coupled to the increased longevity of primates relative to short-lived species such as the mouse. However, a very long lived mammal like the blue whale do not have the C321 residue, for instance.
5. Are there stress situations where the seemingly dramatic treatment of cells with hydrogen peroxide or PR619 is mimicking a physiologically relevant oxidative stress stimulus?
6. Will not most proteins with multiple cysteines form DLC under the conditions used in Fig 1. The author should show some controls to show if this is a specific effect or not.
7. Is there a quantifiable statistical significant enrichment of NDP52 DLC in the mitochondrial fraction shown in Fig 1D?
8. In Fig S1 where it is argued that treatment with O&A induces DLC of NDP52 and not for p62 and OPTN there is loaded much less p62 on the gels than NDP52. This will disguise formation of DLC of p62 here and the experiment needs to be done with equal input amounts of the proteins to be compared.
9. When making a quadruple Cys mutant the authors should show some control experiments to test that the structure of NDP52 is not compromised by these mutations. Can the CC domain still form a dimer in solution as determined in Nat Commun 2013;4:1613. doi: 10.1038/ncomms2606 ?
10. From the results in Fig. 3a-b it is clear that the 4Cys mutant NDP52 can still effectively induce mitophagy as monitored by formation of red dots with the mt-Keima reporter, although the kinetics is slower than with the WT NDP52. The experiments in Fig 4 are done at 2h of O&A treatment and shows less recruitment of ATG13 and ATG16L1 to mitochondria with the mutant NDP52 relative to the WT. However, based on what we see in Fig. 3A-b, if the experiment was done at 3h most likely there would be significant recruitment of the two basal autophagy components.
11. This manuscript is in a short report format and would as such be more suitable for EMBO Reports.
Minor points:
In the legend to Fig. 2e it is stated "Schematic diagram of the NDP52 structure..." This should be corrected to "Schematic diagram of the NDP52 domain organization..." to be precise.
Line 2 from the bottom of page 4: The authors here fail to mention FIP200 since the Vargas et al. 2019 paper ref shows that NDP52 interacts with the ULK1 complex through FIP200.

Referee #3:

This is an interesting, if brief, study into the mechanism of how selective autophagy receptors may mediate mitophagy. The authors find that p62, NDP52 and OPTN form disulphide-linked condensates (oligomers) upon direct oxidation but only NDP52 does so upon mitochondrial toxification. They identified that the oligomerisation is mediated through multiple Cys residues, and mutation of these blunts the recruitment of autophagy proteins and the execution of mitophagy. The identification of this mechanism addresses an important unknown aspect of how mitochondrial dysfunction that requires degradation is signalled to

the autophagy machinery for appropriate engulfment.

Nevertheless, as is typical, this raises many more questions about the mechanism such as the nature of the oxidative signal (O_2^-, H_2O_2 or others) and how it is transmitted from (presumably) inside the mitochondrion externally to NDP52. Indeed, it would be relevant to know more precisely from where the ROS originates (e.g., complex I or complex III), perhaps using specific complex inhibitors to block production. Or how (or if) this is stimulated by more physiologically-relevant triggers such as mtDNA mutations or unfolded proteins. Given the nature of the mechanism discussed here, it is surprising that the authors don't discuss the recent Gan/Komander paper which describes an oxidative stress/H_2O_2 induced regulation of PINK1.
Also, the authors raise an interesting discussion point around the evolutionary acquisition of redox sensitivity in p62, resulting in additional functionality in vertebrates, but it is equally noteworthy that NDP52 (and OPTN) appears to be a vertebrate-specific acquisition. This is in contrast to PINK1 and Parkin which are conserved in many invertebrate species, and so raises pertinent questions about the conserved versus evolved mechanisms of PINK1-Parkin mitophagy.
It's also unclear why NDP52 oxidation/oligomerisation state affects recruitment of autophagy initiators.

Besides these open questions, I have only minor comments for the authors to consider.
- Fig. 1. The purpose of the PRX3/PRX-SO3 blots is not explained (to the naïve reader), nor are details of the reagents given.
- in Fig. 1, # is used to designate non-specific bands but not in Fig. S1. Consistency would be beneficial.
- Fig. 2. It isn't explained why some constructs are shown in red text. Presumably the authors consider wanted to highlight these are the most affected ones(?), but this doesn't hold for 2a where C18A arguably has the biggest effect.
- It would be informative to show the location of the Cys residues on a 3D model.
- Fig. 3. A # is used as a statistical indication in 3b but not explained in the legend.
- Ensure that all references to PINK1 are not abbreviate as "PINK" (e.g. start of Discussion). Also, the formal HUGO name has dropped the "putative" kinase (e.g. Intro).

We are grateful to the Referees for their constructive criticism. We have now performed extensive revision experiments which further support our original hypothesis that NDP52 mediates redox sensing during mitophagy in human cells. Please see below our point-by-point response to Referees' questions below.

Referee #1:

Mitophagy is an important process to remove damaged mitochondria. It has been proposed that mitochondrial dysfunction is also associated with an increased generation of reactive oxygen species and it is thus tempting to speculate that increased ROS generation can modulate mitophagy. In the present manuscript, Kataura and colleagues propose that one such mechanism is ROS-dependent cysteine oxidation of NDP52 and the formation of oligomers that facilitates recruitment of the autophagy machinery.
This is a nice technically well executed study that presents the formation of ROS-mediated formation of NDP52 oligomers upon oxidative insult and harsh conditions used to induce mitophagy, identifies the involved cysteines and shows that mutation of these cysteines hampers mitophagy.
I have a few major points that should be addressed to improve the manuscript.

1/ the treatments applied to induce NDP52 oligomerisation are quite harsh (I am aware of the fact that these conditions are often used for mechanistic studies of mitophagy). I miss experiments employing physiologically more meaningful (i.e. milder) conditions to demonstrate a physiological significance of the disulphide-dependent oligomerisation and the link to mitophagy
Thank you for this comment, to address it we used several approaches. For example, in another project in the lab we found that mitochondrial turnover via mitophagy is enhanced when cells are forced to respire using galactose media conditions (Kataura, Sedlackova et al. 2022). However, in these milder conditions we do not see activation of mitophagy by NDP52 (Rebuttal Fig R1). This result is consistent with the common view of PINK1/Parkin mitophagy as a mechanism of rapid elimination of damaged (membrane depolarisation and generation of excessive ROS) mitochondria that are potentially dangerous to the cell.

We therefore turned to different types of mitochondrial damage. In our new experiments we were able to demonstrate that the damage to either mitochondrial Complex I or Complex III is sufficient to trigger PINK1/Parkin/NDP52-dependent mitophagy (Fig 5A, C). Another stimulus we used is G-TTP which causes accumulation of misfolded proteins in mitochondria (Fiesel, James et al. 2017) and was found to similarly activate PINK1/Parkin/NDP52-dependent mitophagy in the redox-dependent manner (Fig EV3C-F). We therefore believe that the mechanism described in the manuscript is relevant to several types of mitochondrial damage.

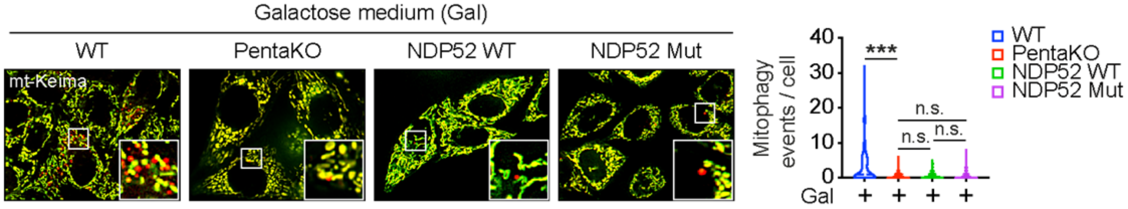

Rebuttal Fig R1. NDP52 is not involved with basal mitophagy in respiring cells
Fluorescence images and quantification of mitophagy in HeLa WT, PentaKO, PentaKO + NDP52 WT and PentaKO + NDP52 C18, 153, 163, 321S (Mut) cells expressing YFP-Parkin

and mt-mKeima cultured in galactose medium (Gal) for 4 days. Data are displayed as cell popular violin plots and *P* values were calculated by one-way ANOVA followed by Sidak test on three technical replicates. ***, $p<0.001$; ns (non-significant).

2/ Is the induction of disulphide bond-dependent oligomerization reversible, i.e. can addition of e.g. DTT to cells reverse it and stop mitophagy induction

As suggested by the Reviewer we tested DTT which indeed was found to reduce NDP52 DLC and suppress mitophagy (Fig 5F, G).

3/ Mutation of multiple cysteines in NDP52 might impair its folding. How can the authors exclude that it is not misfolding that hampers initiation of mitophagy instead of the proposed redox transition?

We analysed recombinant wild type and mutant NDP52 and could demonstrate that these are indistinguishable on analytical SEC and NATIVE-PAGE (Fig 5C, D). This is consistent with our analyses of wild type and mutant proteins by MultiCoil2, indicating the same propensity to form CC dimer (Rebuttal Fig R2). Analysis via Aggrescan3D tool also indicated no effect of Cys mutants on NDP52 structure. To confirm that the protein is well folded and functional, in collaboration with Sascha Martens lab we performed a bead assay (Turco, Witt et al. 2019) in which we found that wild type and mutant NDP52 are able to recruit full length FIP200 to ubiquitin coated beads. The quantification confirms that in reducing conditions there is no difference between the WT and mutant, which implies that the NDP52 mutant still binds

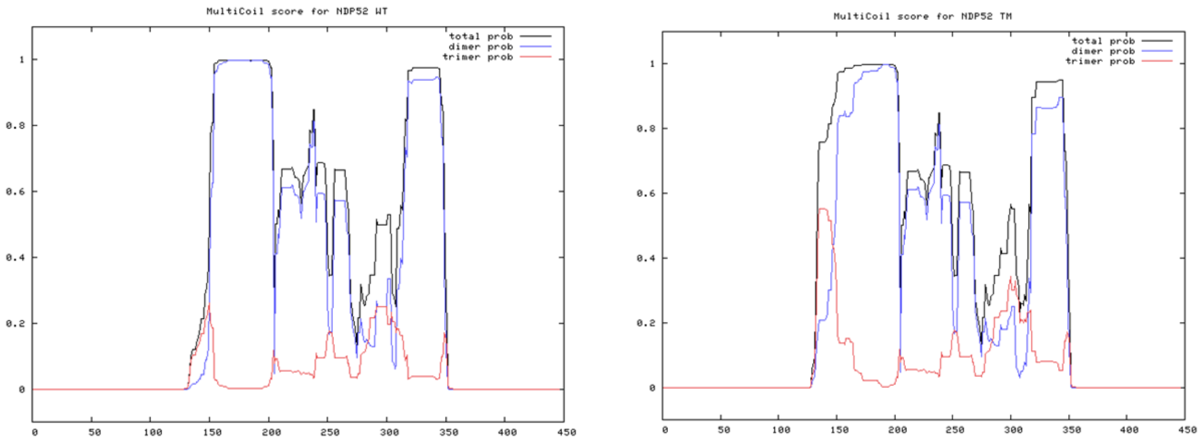

potently to ubiquitin and can still recruit FIP200 (Fig EV2D).

Rebuttal Fig R2. Mutation of cysteine residues C153S, C163S, and C321S within CC domain of NDP52 do not affect propensity to form CC dimer.
Analysis of wild type (left) and Cys mutant (right) NDP52 using MultiCoil2 software. Note similar profiles of predicted CC dimerization between the graphs.

Minor Points

1/ I think it is a bit unfortunate that reducing and non-reducing conditions are always presented on separate gels/blots. It is hard to estimate amounts and shifts between different species of NDP52

As suggested, we repeated some experiments to show both non-reduced and reduced condition in one gel (Fig EV1B).

2/ why is oligomerization important for induction of mitophagy? The underlying mechanism could be more extensively discussed.

Work from Sascha Martens lab showed that oligomerisation of SARs, particularly p62, is the means to increase avidity of binding to ubiquitin and other components of autophagy machinery (Turco, Witt et al. 2019). In collaboration with Martens lab, we attempted to obtain insights into the underlying mechanism by following the recruitment of FIP200 to ubiquitin coated beads by NDP52 in presence and absence of H_2O_2. However, even though various conditions were tested, the FIP200 protein precipitated upon H_2O_2 addition rendering it impossible to draw sound conclusions from the experiment. For this reason, we turned to cells where we investigated binding of ULK1 complex components FIP200, ULK1 and ATG13 to wild type and mutant NDP52 by co-IP (Vargas, Wang et al. 2019). We found that Cys mutant was binding less FIP200, ULK1 and ATG13 in conditions of mitochondrial damage which induce DLC formation, consistent with the reduced avidity of interaction in the absence of NDP52 oligomerisation (Fig 6D). We hope that this experimental confirmation of reduced binding, in addition to reduced recruitment of autophagy machinery to damaged mitochondria (Fig 6A, B), is sufficient to support our model of NDP52 DLC as the scaffolding platform promoting the initiation of mitophagy.

Referee #2:

In this report format paper the Korolchuk group follows up on their original discovery on redox regulation of oligomerization of p62 (Nat Commun. 2018 Jan 17;9(1):256. doi: 10.1038/s41467-017-02746-z. PMID: 29343728), but this time turning their attention to another soluble autophagy receptor, NDP52 (CALCOCO2), and its role in mitophagy. Damaged mitochondria produce ROS (reactive oxygen species) but the mechanism(s) on how this is sensed to induce mitophagy is/are not clarified. Here, Kataura et al. present data to suggest that NDP52 has several redox sensitive cysteines that form disulphide bonds to oligomerize NDP52 in the presence of ROS. Mitochondria-derived ROS can induce oligomerization of NDP52 on the mitochondria, due to formation of disulphide-linked conjugates (DLC), to facilitate recruitment of the basal autophagy apparatus for effective induction of mitophagy.
The model suggested here is interesting, but there is not enough evidence in the present version of this paper to properly support the conclusions made. Also, the impact the 4Cys mutant of NDP52 has on the induced mitophagy is on the kinetics and it is not a strong effect that reduces strongly the mitophagy observed.
Some specific major points:

1. The evidence for ROS-induced oligomerization rests on only one assay method, gel electrophoresis under non-reduced conditions. It is important to also use gel filtration/SEC-MALS (see below) to test this.

We attempted to detect DLC using analytical SEC, however faced a technical issue: NDP52 is isolated in reducing buffer and has a relatively high percentage of DTT in the final sample loaded on the SEC-column in order to have sufficient amounts of protein for its detection (and loading more protein thus also means loading more DTT). The analytical SEC only handles small volumes, and we could thus not dilute our samples further. The outcome of this is that only a small fraction of NDP52 was converted to DLC (not shown), compared to our

analyses by SDS-PAGE (Fig 3B). Unfortunately, we also could not run whole cell lysates on our analytical columns as these are restricted for use with pure recombinant proteins only.

Therefore, instead we employed BN-PAGE which demonstrated the formation of WT NDP52 oligomers, whilst the formation of higher order species was impaired by Cys mutations (Fig 3D).

The data are presented from experiments done only on HeLa cells. No neuronal cell model or murine (or other animal) models are used to try to test the in vivo relevance of the disulphide-linked conjugates (DLC) formation in PINK1-PARKIN-dependent mitophagy. These are important weaknesses of this study that reduces the impact of the findings presented here.

We discussed the possibility of generating triple knockout (NDP52/OPTN/TAX1BP1) iPSC cells with Michael Lazarou who has already attempted this and found that the knockout completely impairs neuronal differentiation. This finding is consistent with the previously reported deficiency of PINK1-mediated mitophagy leading to the compromise in pluripotency in ES cells (Wang, Liu et al. 2021). Therefore, we could not reconstitute our experimental system in stem-cell derived human neurons.

Mouse (or other mammals except apes) also cannot be used as a model to interrogate the loss of this mechanism as NDP52 in lower mammals does not have redox-sensitive Cys residues (Fig 2F). We therefore undertook a reverse approach – to introduce human NDP52 into mouse embryonic fibroblasts (MEFs). Human wild type but not Cys mutant NDP52 was sufficient to initiate ROS-dependent mitophagy in MEFs (Fig 6E, EV5C-F). We believe these data provide support for our conclusion that this mechanism is specific to humans, which has implications for the studies of mitophagy in animal models.

2. It has been shown previously by size-exclusion chromatography with multi-angle light scattering (SEC-MALS) that NDP52 behaves as a dimer in solution mediated by its central coiled-coil (CC) domain (Nat Commun 2013;4:1613. doi: 10.1038/ncomms2606). Further work by the same group showed the CC domain of NDP52 to form a parallel dimer (Sci. Rep. 7:43318. DOI: 10.1038/srep43318). This means that NDP52 is most likely already a dimer when recruited to mitochondria. In the present paper no attempt has been done to relate the ROS induced DLC formation, and the Cys residues implicated by mutations to be responsible, to the structure of NDP52. The apparent MW from gel electrophoresis and WB detection shown in Fig 1 suggest that a trimer of NDP52 may be formed. This is not discussed or mentioned at all by the authors. How does this relate to NDP52 being a parallel dimer, or is it a dimer we primarily see in the non-reduced gels? The authors need to provide some data to explore this.

We now analysed the effect of Cys mutations on the formation of parallel dimer. This is described in Fig 3 and EV2 and associated text.

3. The four Cys residues being important for DLC formation are located in the SKICH domain (C18) and in the large central CC domain (C153, 163 and 321) and as such spaced widely apart. It is difficult to understand why a Cys residue in the SKICH domain should be required. Can the authors rationalize this in any way?

We have now analysed effect of individual Cys mutations on mitophagy, confirming their additive effect and therefore contribution of all 4 residues to the function of NDP52 as a redox sensor (Fig EV2G). Modelling of NDP52 dimer and tetramer indicated that C163 and

C321 are involved in disulphide formation within the dimer, potentially stabilising it. Instead, C153 and C18 are accessible for the formation of disulphides between the dimers thus potentially contributing to the formation of higher order oligomers (Fig 3A, D, EV2A-C).

4. As pointed out by the authors, all the four Cys residues implicated by mutations in the formation of DLC are not evolutionary conserved outside primates. Even the Rhesus monkey does not have all four of them conserved. This speaks against a major role. The authors argue that this is a mechanism evolved to tackle oxidative stress and is coupled to the increased longevity of primates relative to short-lived species such as the mouse. However, a very long lived mammal like the blue whale do not have the C321 residue, for instance.
Mechanisms of longevity have evolved differently in different species, and we completely agree that we can learn a lot from studying other long-lived species. However, with the tools available to us we could only use reverse engineering strategy described above, and we believe that making mouse cells to perform ROS-dependent mitophagy by introducing human NDP52 is a convincing evidence for the difference between the mechanisms of mitophagy in humans and other mammals.

5. Are there stress situations where the seemingly dramatic treatment of cells with hydrogen peroxide or PR619 is mimicking a physiologically relevant oxidative stress stimulus?
In the manuscript we show that DLC stabilisation (and subsequent degradation via autophagy) results from oxidative stress conditions where antioxidant systems such as thioredoxin/thioredoxin reductase are breached (Fig 1B). We agree that hydrogen peroxide and PR619 are blunt tools, however these are only used to show that some (but not all) proteins are redox-sensitive (Appendix Fig S1). In normal cells oxidative stress would happen at the sub-cellular level, for example in the vicinity of ROS-producing mitochondria, and indeed we detect NDP52 DLC formation on damaged mitochondria (Fig 1D, 4C). However, even in unstressed cells, blocking autophagic flux by bafilomycin A1 is sufficient to accumulate DLC, suggesting that NDP52 is oxidised and forms oligomers which are degraded by autophagy under basal conditions (Fig 1C, 1D, 4C). This suggests to us that localised oxidative stress and autophagy as a mechanism of oxidative stress response also occur in cells that have not been exposed to harsh experimental conditions.

6. Will not most proteins with multiple cysteines form DLC under the conditions used in Fig 1. The author should show some controls to show if this is a specific effect or not.
As suggested by the Reviewer, we tested several proteins in conditions of oxidative stress. Interestingly, in addition to SARs two other autophagy proteins (ATG5 and ATG7) appear to form DLC (ATG7 has previously been shown to be redox-sensitive (Frudd, Burgoyne et al. 2018)). Instead, several other proteins, including TFEB which is involved in autophagy regulation at the transcriptional level, as well as tested by us mitochondrial and cytoplasmic proteins, have not shown any signs of oligomerisation (Appendix Fig S1). This suggests that autophagy machinery is either particularly sensitive to or specifically evolved to be responsive to oxidative stress conditions.

7. Is there a quantifiable statistical significant enrichment of NDP52 DLC in the mitochondrial fraction shown in Fig 1D?
We have now added quantifications of DLC in these conditions (Fig 1D).

8. In Fig S1 where it is argued that treatment with O&A induces DLC of NDP52 and not for p62 and OPTN there is loaded much less p62 on the gels than NDP52. This will disguise

formation of DLC of p62 here and the experiment needs to be done with equal input amounts of the proteins to be compared.
We have repeated these experiments and showed all proteins at the same exposure (Fig EV1B). As discussed in the manuscript, compared to p62 and OPTN, only NDP52 shows clear induction of DLC that are degraded by autophagy in these conditions.

9. When making a quadruple Cys mutant the authors should show some control experiments to test that the structure of NDP52 is not compromised by these mutations. Can the CC domain still form a dimer in solution as determined in Nat Commun 2013;4:1613. doi: 10.1038/ncomms2606 ?
As discussed above, we detected no differences in the stability, behaviour on analytical SEC or NATIVE-PAGE, as well as binding to ubiquitin and FIP200 between wild type and mutant protein in reduced conditions. Molecular modelling also indicated that Cys mutations do not affect the formation of CC dimer, however functional studies indicate that the formation of disulphides within the dimer (mediated by Cys 163 and 321) are important for the mitophagy induction (Rebuttal Fig R2, Fig EV2G), and we hypothesise that these could either promote dimerisation or stabilise the dimer.

10. From the results in Fig. 3a-b it is clear that the 4Cys mutant NDP52 can still effectively induce mitophagy as monitored by formation of red dots with the mt-Keima reporter, although the kinetics is slower than with the WT NDP52. The experiments in Fig 4 are done at 2h of O&A treatment and shows less recruitment of ATG13 and ATG16L1 to mitochondria with the mutant NDP52 relative to the WT. However, based on what we see in Fig. 3A-b, if the experiment was done at 3h most likely there would be significant recruitment of the two basal autophagy components.
Indeed, after 3 hours of AO treatment recruitment of autophagy initiation proteins is normalised between wild type and mutant NDP52-expressing cells (Fig EV6B). This again supports our conclusion that oxidation/oligomerisation of NDP52 promotes the speed at which damaged mitochondria are removed.

11. This manuscript is in a short report format and would as such be more suitable for EMBO Reports.
Based on constructive criticism from the Reviewers we significantly expanded the dataset and hope that the revised manuscript would be appropriate for the publication in *EMBO J*.

Minor points:
In the legend to Fig. 2e it is stated "Schematic diagram of the NDP52 structure..." This should be corrected to "Schematic diagram of the NDP52 domain organization..." to be precise.
We have now corrected this in the text.

Line 2 from the bottom of page 4: The authors here fail to mention FIP200 since the Vargas et al. 2019 paper ref shows that NDP52 interacts with the ULK1 complex through FIP200.
We have now added the reference to previous FIP200 paper in the respective text and experimentally investigated the interaction with FIP200 (Fig 6D).

Referee #3:
This is an interesting, if brief, study into the mechanism of how selective autophagy receptors may mediate mitophagy. The authors find that p62, NDP52 and OPTN form disulphide-linked condensates (oligomers) upon direct oxidation but only NDP52 does so upon

mitochondrial toxification. They identified that the oligomerisation is mediated through multiple Cys residues, and mutation of these blunts the recruitment of autophagy proteins and the execution of mitophagy. The identification of this mechanism addresses an important unknown aspect of how mitochondrial dysfunction that requires degradation is signalled to the autophagy machinery for appropriate engulfment.

Nevertheless, as is typical, this raises many more questions about the mechanism such as the nature of the oxidative signal (O2-, H2O2 or others) and how it is transmitted from (presumably) inside the mitochondrion externally to NDP52.
Indeed, it would be relevant to know more precisely from where the ROS originates (e.g., complex I or complex III), perhaps using specific complex inhibitors to block production. Or how (or if) this is stimulated by more physiologically-relevant triggers such as mtDNA mutations or unfolded proteins.
Upon mitochondrial damage induced by inhibitors of mitochondrial complexes or accumulation of unfolded proteins, oxidation-defective Cys mutant was equally translocated to mitochondrial fraction compared to WT (Fig 6A, B, EV5A, B, E), and mitochondrial ROS inhibitors suppressed DLC formation of NDP52 but not Parkin recruitment (Fig 4E, 5E, EV3A, C, D, 4C, D, 5F). These data suggest that NDP52 is initially accumulated onto the surface of damaged mitochondria ubiquitinated by PINK1/Parkin, and in turn oxidised by ROS released from the mitochondria.
As suggested by the Reviewer, we performed experiments by utilising rotenone and antimycin (inhibitors of mitochondrial complex I and III, respectively) and S1QEL and S3QEL (suppressors of mitochondrial ROS produced from complex I and III, respectively) to investigate the exact origin of ROS. We found that ROS produced from either mitochondrial complex I or complex III is sufficient to trigger DLC formation of NDP52 and mitophagy (Fig 5, EV4).
Given that 1) mitoSOX is a selective indicator of mitochondrial superoxide; 2) rotenone and antimycin induce mitochondrial superoxide release via inhibiting complex I and III, respectively; we speculate that O2- (initially) mediates the oxidation of NDP52.

Given the nature of the mechanism discussed here, it is surprising that the authors don't discuss the recent Gan/Komander paper which describes an oxidative stress/H2O2 induced regulation of PINK1.
We have expanded Discussion in the text.

Also, the authors raise an interesting discussion point around the evolutionary acquisition of redox sensitivity in p62, resulting in additional functionality in vertebrates, but it is equally noteworthy that NDP52 (and OPTN) appears to be a vertebrate-specific acquisition. This is in contrast to PINK1 and Parkin which are conserved in many invertebrate species, and so raises pertinent questions about the conserved versus evolved mechanisms of PINK1-Parkin mitophagy.
We have expanded Discussion in the text.

It's also unclear why NDP52 oxidation/oligomerisation state affects recruitment of autophagy initiators.
We have addressed to this concern as above (Please see our response to Reviewer #1 minor point /2). In brief, we found that oxidation-deficient NDP52 binds less to ULK1 complex component proteins that initiate autophagy/mitophagy (Fig 6D). Therefore, we propose that oxidised/oligomerised NDP52 localised onto the mitochondrial surface of damaged mitochondria can be a scaffold for the autophagy initiators.

Besides these open questions, I have only minor comments for the authors to consider.

- Fig. 1. The purpose of the PRX3/PRX-SO3 blots is not explained (to the naïve reader), nor are details of the reagents given.
We have now revised the text and figure legend.

- in Fig. 1, # is used to designate non-specific bands but not in Fig. S1. Consistency would be beneficial.
We have now revised the text and figure legends.

- Fig. 2. It isn't explained why some constructs are shown in red text. Presumably the authors consider wanted to highlight these are the most affected ones(?), but this doesn't hold for 2a where C18A arguably has the biggest effect.
We have now revised the figure legends.

- It would be informative to show the location of the Cys residues on a 3D model.
We have now added a 3D model with labels of the Cys residues (Fig 3A).

- Fig. 3. A # is used as a statistical indication in 3b but not explained in the legend.
We have now revised the figure legends.

- Ensure that all references to PINK1 are not abbreviate as "PINK" (e.g. start of Discussion). Also, the formal HUGO name has dropped the "putative" kinase (e.g. Intro).
We have now revised the text.

References

Fiesel, F. C., E. D. James, R. Hudec and W. Springer (2017). "Mitochondrial targeted HSP90 inhibitor Gamitrinib-TPP (G-TPP) induces PINK1/Parkin-dependent mitophagy." Oncotarget **8**(63): 106233-106248.

Frudd, K., T. Burgoyne and J. R. Burgoyne (2018). "Oxidation of Atg3 and Atg7 mediates inhibition of autophagy." Nat Commun **9**(1): 95.

Kataura, T., L. Sedlackova, E. G. Otten, D. Shapira, F. Scialo, R. Stefanatos, K. Ishikawa, G. Kelly, E. Seranova, C. Sun, D. Maetzel, N. Kenneth, S. Trushin, T. Zhang, E. Trushina, C. C. Bascom, R. Tasseff, R. J. Isfort, J. E. Oblong, S. Miwa, M. Lazarou, R. Jaenisch, M. Imoto, S. Saiki, M. Papamichos-Chronakis, O. D. Maddocks, A. Sanz, S. Sarkar and V. I. Korolchuk (2022). "Autophagy is Required for the Maintenance of NAD." CellPress Sneak Peak (Preprint).

Turco, E., M. Witt, C. Abert, T. Bock-Bierbaum, M. Y. Su, R. Trapannone, M. Sztacho, A. Danieli, X. Shi, G. Zaffagnini, A. Gamper, M. Schuschnig, D. Fracchiolla, D. Bernklau, J. Romanov, M. Hartl, J. H. Hurley, O. Daumke and S. Martens (2019). "FIP200 Claw Domain Binding to p62 Promotes Autophagosome Formation at Ubiquitin Condensates." Mol Cell **74**(2): 330-346.e311.

Vargas, J. N. S., C. Wang, E. Bunker, L. Hao, D. Maric, G. Schiavo, F. Randow and R. J. Youle (2019). "Spatiotemporal Control of ULK1 Activation by NDP52 and TBK1 during Selective Autophagy." Mol Cell **74**(2): 347-362 e346.

Wang, C., K. Liu, J. Cao, L. Wang, Q. Zhao, Z. Li, H. Zhang, Q. Chen and T. Zhao (2021). "PINK1-mediated mitophagy maintains pluripotency through optineurin." Cell Prolif **54**(5): e13034.

Dear Viktor,

We have now received re-review reports from three referees, which I attach below. As you will see, you have addressed the majority of concerns satisfactorily. However, the report of referee three is substantive. It will therefore require addressing with a second point-by-point reply (and possibly some textual changes) from you. There are also some remaining minor editorial points which need to be addressed which I include now to prevent one more round of revision. In this regard would you please:

Make sure that figures, track changes and coloured text are removed from the final .doc file
Rename the conflict of interest statement the "DISCLOSURE AND COMPETING INTERESTS STATEMENT"
Complete the AC/CrediT section on our website
Limit references to ten names. For author lists containing more than ten names, please use "et al."
Enter the relevant grant numbers in the relevant section of our website
Add page numbers to the Appendix table of contents
Reorganise the Expanded View and Appendix Figure Source Data file to one file/folder per figure and ZIP

EMBO Press is an editorially independent publishing platform for the development of EMBO scientific publications.

Best wishes,

William

William Teale, PhD
Editor
The EMBO Journal
w.teale@embojournal.org

https://emboj.msubmit.net/cgi-bin/main.plex

Referee #1:

The authors have addressed my concerns. The provided additional data and controls in my opinion strengthened the manuscript and I would thus support publication in EMBO J.

Referee #2:

I find the the authors´ responses and revision work related to my comments satisfactory.
It is still a question, as I find it, if the story will be more suited for EMBO Reports.

Referee #3:

The authors have responded to my original comments adequately although the 'expanded discussion' points are limited to simple mentions, but they have also added substantial new data that provides further insight into the potential mechanism at play.

Notwithstanding, there are several points mainly on the new data and interpretation but also on Materials and Methods that require further clarification.

p7/Fig. 3A. This figure (and associated data) is a valuable addition as it adds greater depth to the potential molecular mechanism of DLCs but it is confusingly described: "...indicated that C163 and C321 form intra-dimer..." - C321 is not shown on this figure; "...C153 is predicted to be positioned away from the dimer interface ..." - this looks to be close to the interface. Do you mean C18? If the authors consider C153 'away from the interface' it could be beneficial to indicate the interface region.

Several of the experimental procedures require more detail and some clarifications. For instance, for the Mitophagy assay, more detail is needed on the method to identify (and isolate for quantification) the mitolysosomes. I doubt that the authors simply subtract the 480nm signal from the 561nm signal, as stated, as this would not exclude inappropriately large or small objects. Some thresholding is surely used.

Likewise, I'm confused at how the MitoSOX is being used. Indeed, it is a reasonable reporter for mitochondrial superoxide but requires a substantial mitochondrial membrane potential to accumulate there, so it is surprising that in conditions that the authors say depolarises (e.g. RO in Fig. 5, EV4) that MitoSOX is still accumulating in mitochondria. One would expect this distribution to look more like it does in AO. This raises the question of what ROS is being reported in AO conditions (cytosolic?) and indeed how the quantifications are done. It is well known, and evident in the AO images, that MitoSOX can intercalate nuclear DNA. Do the authors take steps to remove this from their quantification as it would artificially inflate the result. It might be beneficial to the authors to use an alternative approach to measure ROS that doesn't rely so much on mitochondrial membrane potential.

Regarding the response to my main original critique concerning the nature of the ROS moiety, it is incongruous to suggest that mitochondrial superoxide is what mediates the oxidation of NDP52 as superoxide (being a charged molecule) will not cross the mitochondrial membranes.

Minor:
p10. It is inaccurate to state "oligomycin, which blocks electron transport chain at the level of Complex V" since Complex V is not part of the ETC which is comprised of Complexes I-IV.

p12. It is also inaccurate to say "all 4 [Cys] residues present only in apes " as all 4 are also present in cow and wolf, while one (C321) is absent in macaque.

Referee #1:

The authors have addressed my concerns. The provided additional data and controls in my opinion strengthened the manuscript and I would thus support publication in EMBO J.

Referee #2:

I find the the authors´ responses and revision work related to my comments satisfactory. It is still a question, as I find it, if the story will be more suited for EMBO Reports.

We would like to thank both Reviewers for their critical appraisal of our work.

Referee #3:

The authors have responded to my original comments adequately although the 'expanded discussion' points are limited to simple mentions, but they have also added substantial new data that provides further insight into the potential mechanism at play.
Notwithstanding, there are several points mainly on the new data and interpretation but also on Materials and Methods that require further clarification.
p7/Fig. 3A. This figure (and associated data) is a valuable addition as it adds greater depth to the potential molecular mechanism of DLCs but it is confusingly described: "...indicated that C163 and C321 form intra-dimer..." - C321 is not shown on this figure; "...C153 is predicted to be positioned away from the dimer interface ..." - this looks to be close to the interface. Do you mean C18? If the authors consider C153 'away from the interface' it could be beneficial to indicate the interface region.

As shown in new Fig 3A and EV2A, whilst our simulation model indicates the interaction and disulphide formation between C163-C163 as well as C321-C321 within a dimer, C153 is predicted to be faced away from the dimer interface, suggesting C153 is available for the interaction between two dimers to generate an anti-parallel tetramer and higher order oligomers as visualised in Fig 3A. Our model indicates that C18 is also exposed to the surface of the SKICH domain, which could further involve C18-mediated oligomerisation (i.e. hexamer, octamer and so on). We have revised the manuscript to explain this clearly.

Several of the experimental procedures require more detail and some clarifications. For instance, for the Mitophagy assay, more detail is needed on the method to identify (and isolate for quantification) the mitolysosomes. I doubt that the authors simply subtract the 480nm signal from the 561nm signal, as stated, as this would not exclude inappropriately large or small objects. Some thresholding is surely used.

As pointed out by the Reviewer, we indeed applied thresholding to quantify the number of mitolysosomes. We have now revised the text and added detailed procedure to the Methods section.

Likewise, I'm confused at how the MitoSOX is being used. Indeed, it is a reasonable reporter for mitochondrial superoxide but requires a substantial mitochondrial membrane potential to accumulate there, so it is surprising that in conditions that the authors say depolarises (e.g. RO in Fig. 5, EV4) that MitoSOX is still accumulating in mitochondria. One would expect

this distribution to look more like it does in AO. This raises the question of what ROS is being reported in AO conditions (cytosolic?) and indeed how the quantifications are done. It is well known, and evident in the AO images, that MitoSOX can intercalate nuclear DNA. Do the authors take steps to remove this from their quantification as it would artificially inflate the result. It might be beneficial to the authors to use an alternative approach to measure ROS that doesn't rely so much on mitochondrial membrane potential.
Regarding the response to my main original critique concerning the nature of the ROS moiety, it is incongruous to suggest that mitochondrial superoxide is what mediates the oxidation of NDP52 as superoxide (being a charged molecule) will not cross the mitochondrial membranes.

As the Reviewer pointed out, MitoSOX relies on mitochondrial membrane potential (negative mitochondrial matrix) to accumulate in mitochondria through its lipophilic triphenylphosphonium cation (TPP^+) moiety (Polster *et al*, 2014). Indeed, RO induced mitochondrial depolarisation assessed by TMRM staining, however, TMRM signal in RO-treated cells was still detectable and clearly higher than the signal from AO-treated cells, which suggests that residual membrane potential was sufficient to the MitoSOX accumulation in mitochondria (Fig EV4B). Apart from its dependency of mitochondrial membrane potential on mitochondrial targeting, ROS-sensing by MitoSOX is conferred by its non-red fluorescent hydroethidine (HE) moiety (Robinson *et al*, 2006). HE is oxidised by O_2^- to form 2-hydroxyethidium (2-OH-E^+) and further oxidised by O_2^- and other oxidants to form ethidium (E^+). Both 2-OH-E^+ and E^+ are red fluorescent and cationic, which becomes able to intercalate into DNA and double-stranded RNA (Shchepinova *et al*, 2017). Thus, although the signal from nucleus does not report where MitoSOX is oxidised, it indicates overall intracellular ROS is elevated as MitoSOX is oxidised (in mitochondrial or in cytosol) and then relocates to the nucleus. Consistently, no nucleus stained by MitoSOX was observed at basal conditions, suggesting increase of ROS is required to observe nuclear staining (Fig 5B). Indeed, we quantified signal intensity from whole area of each cell and did not remove the signals from nucleus, and we agree that the data do not display “ROS in the mitochondria”. Thus, we conclude AO elevates overall intracellular ROS, and we have rephrased “mitochondrial ROS” to “ROS induced by mitochondrial damage”, or “ROS released from mitochondria”. Both mitochondrial depolarisation and ROS induction by mitochondrial damage are required for NDP52-mediated mitophagy (Fig 4D, 5A-D, EV3E, F, EV4B). Mitochondrial membrane depolarisation is associated with the opening of mitochondrial permeability transition pore (mPTP), which leads to the release of ROS (superoxide and H_2O_2) from the mitochondria (Zorov *et al*, 2014). Thus, AO/RO-induced mitochondrial damage could be speculated to elevate ROS both in the mitochondria (triggered by complex I/III inhibition) and in the cytosol (released from mitochondria), which can oxidise NDP52. As described above, HE oxidation is mainly mediated by superoxide, therefore, we conclude that oxidation of NDP52 is primarily mediated by superoxide produced by and released from mitochondria. We have added our interpretation of these data to the discussion section.

Minor:
p10. It is inaccurate to state "oligomycin, which blocks electron transport chain at the level of Complex V" since Complex V is not part of the ETC which is comprised of Complexes I-IV.

As suggested by the reviewer, we have revised the text.

p12. It is also inaccurate to say "all 4 [Cys] residues present only in apes " as all 4 are also present in cow and wolf, while one (C321) is absent in macaque.

Thank you for this comment. We examined two available sequences of cow (cattle, *Bos taurus*) NDP52 (Accession number: XP_024835379.1 and XP_005220542.3), however, both of them do not contain C321. Instead, wolf (*Canis lupus dingo*) NDP52 sequence (Accession number: XP_025295713.1) has the four Cys residues which could be speculated to result from convergent evolution. We also agree that the residue of C321 is not conserved in macaques (*Macaca fascicularis* and *Macaca mulatta*) (Accession number: XP_005583642.1 and XP_014974835.2, respectively) however macaque is not classified as apes (Hominoidea). We have revised the relevant text accordingly.

References

Polster BM, Nicholls DG, Ge SX, Roelofs BA (2014) Use of potentiometric fluorophores in the measurement of mitochondrial reactive oxygen species. *Methods Enzymol* 547: 225-250

Robinson KM, Janes MS, Pehar M, Monette JS, Ross MF, Hagen TM, Murphy MP, Beckman JS (2006) Selective fluorescent imaging of superoxide in vivo using ethidium-based probes. *Proc Natl Acad Sci U S A* 103: 15038-15043

Shchepinova MM, Cairns AG, Prime TA, Logan A, James AM, Hall AR, Vidoni S, Arndt S, Caldwell ST, Prag HA *et al* (2017) MitoNeoD: A Mitochondria-Targeted Superoxide Probe. *Cell Chem Biol* 24: 1285-1298 e1212

Zorov DB, Juhaszova M, Sollott SJ (2014) Mitochondrial reactive oxygen species (ROS) and ROS-induced ROS release. *Physiol Rev* 94: 909-950

Dear Viktor,

I am pleased to inform you that your manuscript has been accepted for publication in the EMBO Journal.

Congratulations on a really nice study!

Referee #3:

The authors have answered my new comments very well and clarified the remaining confusions. The additional amendments made from this round have further increased the quality and accuracy of the manuscript and I'm happy to support publication. Congratulations to the authors on elegant work.

Please note that it is EMBO Journal policy for the transcript of the editorial process (containing referee reports and your response letter) to be published as an online supplement to each paper. If you do NOT want this, you will need to inform the Editorial Office via email immediately. More information is available here: https://www.embopress.org/page/journal/14602075/authorguide#transparentprocess

Your manuscript will be processed for publication in the journal by EMBO Press. Manuscripts in the PDF and electronic editions of The EMBO Journal will be copy edited, and you will be provided with page proofs prior to publication. Please note that supplementary information is not included in the proofs.

You will be contacted by Wiley Author Services to complete licensing and payment information. The required 'Page Charges Authorization Form' is available here: https://www.embopress.org/pb-assets/embo-site/tej_apc.pdf - please download and complete the form and return to embopressproduction@wiley.com

EMBO Press participates in many Publish and Read agreements that allow authors to publish Open Access with reduced/no publication charges. Check your eligibility: https://authorservices.wiley.com/author-resources/Journal-Authors/open-access/affiliation-policies-payments/index.html

Should you be planning a Press Release on your article, please get in contact with embojournal@wiley.com as early as possible, in order to coordinate publication and release dates.

If you have any questions, please do not hesitate to call or email the Editorial Office. Thank you for your contribution to The EMBO Journal.

Best wishes,

William

William Teale, PhD
Editor
The EMBO Journal
w.teale@embojournal.org

** Click here to be directed to your login page: https://emboj.msubmit.net

EMBO Press Author Checklist

Corresponding Author Name: Viktor Korolchuk	
Journal Submitted to: EMBO Journal	
Manuscript Number: EMBOJ-2022-111372	

USEFUL LINKS FOR COMPLETING THIS FORM
The EMBO Journal - Author Guidelines
EMBO Reports - Author Guidelines
Molecular Systems Biology - Author Guidelines
EMBO Molecular Medicine - Author Guidelines

Reporting Checklist for Life Science Articles (updated January 2022)
This checklist is adapted from Materials Design Analysis Reporting (MDAR) Checklist for Authors. MDAR establishes a minimum set of requirements in transparent reporting in the life sciences (see Statement of Task: 10.31222/osf.io/9sm4x). Please follow the journal's guidelines in preparing your manuscript.
Please note that a copy of this checklist will be published alongside your article.

Abridged guidelines for figures
1. Data
The data shown in figures should satisfy the following conditions:
- the data were obtained and processed according to the field’s best practice and are presented to reflect the results of the experiments in an accurate and unbiased manner.
- ideally, figure panels should include only measurements that are directly comparable to each other and obtained with the same assay.
- plots include clearly labeled error bars for independent experiments and sample sizes. Unless justified, error bars should not be shown for technical replicates.
- if n<5, the individual data points from each experiment should be plotted. Any statistical test employed should be justified.
- Source Data should be included to report the data underlying figures according to the guidelines set out in the authorship guidelines on Data Presentation.

2. Captions
Each figure caption should contain the following information, for each panel where they are relevant:
- a specification of the experimental system investigated (eg cell line, species name).
- the assay(s) and method(s) used to carry out the reported observations and measurements.
- an explicit mention of the biological and chemical entity(ies) that are being measured.
- an explicit mention of the biological and chemical entity(ies) that are altered/varied/perturbed in a controlled manner.
- the exact sample size (n) for each experimental group/condition, given as a number, not a range;
- a description of the sample collection allowing the reader to understand whether the samples represent technical or biological replicates (including how many animals, litters, cultures, etc.).
- a statement of how many times the experiment shown was independently replicated in the laboratory.
- definitions of statistical methods and measures:
  - common tests, such as t-test (please specify whether paired vs. unpaired), simple χ2 tests, Wilcoxon and Mann-Whitney tests, can be unambiguously identified by name only, but more complex techniques should be described in the methods section;
  - are tests one-sided or two-sided?
  - are there adjustments for multiple comparisons?
  - exact statistical test results, e.g., P values = x but not P values < x;
  - definition of ‘center values’ as median or average;
  - definition of error bars as s.d. or s.e.m.

Please complete ALL of the questions below.
Select "Not Applicable" only when the requested information is not relevant for your study.

Materials

Newly Created Materials	Information included in the manuscript?	In which section is the information available? (Reagents and Tools Table, Materials and Methods, Figures, Data Availability Section)
New materials and reagents need to be available; do any restrictions apply?	Not Applicable	

Antibodies	Information included in the manuscript?	In which section is the information available? (Reagents and Tools Table, Materials and Methods, Figures, Data Availability Section)
For **antibodies** provide the following information:<br>- Commercial antibodies: RRID (if possible) or supplier name, catalogue number and or/clone number<br>- Non-commercial: RRID or citation	Yes	Materials and Methods

DNA and RNA sequences	Information included in the manuscript?	In which section is the information available? (Reagents and Tools Table, Materials and Methods, Figures, Data Availability Section)
**Short novel DNA or RNA including primers, probes**: provide the sequences.	Yes	Appendix Table S1-4

Cell materials	Information included in the manuscript?	In which section is the information available? (Reagents and Tools Table, Materials and Methods, Figures, Data Availability Section)
**Cell lines:** Provide species information, strain. Provide accession number in repository **OR** supplier name, catalog number, clone number, and/**OR** RRID.	Yes	Materials and Methods
**Primary cultures:** Provide species, strain, sex of origin, genetic modification status.	Not Applicable	
Report if the cell lines were recently **authenticated** (e.g., by STR profiling) and tested for mycoplasma contamination.	Not Applicable	

Experimental animals	Information included in the manuscript?	In which section is the information available? (Reagents and Tools Table, Materials and Methods, Figures, Data Availability Section)
**Laboratory animals or Model organisms:** Provide species, strain, sex, age, genetic modification status. Provide accession number in repository **OR** supplier name, catalog number, clone number, **OR** RRID.	Not Applicable	
**Animal observed in or captured from the field:** Provide species, sex, and age where possible.	Not Applicable	
Please detail **housing and husbandry conditions**.	Not Applicable	

Plants and microbes	Information included in the manuscript?	In which section is the information available? (Reagents and Tools Table, Materials and Methods, Figures, Data Availability Section)
**Plants:** provide species and strain, ecotype and cultivar where relevant, unique accession number if available, and source (including location for collected wild specimens).	Not Applicable	
**Microbes:** provide species and strain, unique accession number if available, and source.	Not Applicable	

Human research participants	Information included in the manuscript?	In which section is the information available? (Reagents and Tools Table, Materials and Methods, Figures, Data Availability Section)
If collected and within the bounds of privacy constraints report on age, sex and gender or ethnicity for all study participants.	Not Applicable	

Core facilities	Information included in the manuscript?	In which section is the information available? (Reagents and Tools Table, Materials and Methods, Figures, Data Availability Section)
If your work benefited from core facilities, was their service mentioned in the acknowledgments section?	Yes	Acknowledgement

Design

Study protocol	Information included in the manuscript?	In which section is the information available? (Reagents and Tools Table, Materials and Methods, Figures, Data Availability Section)
If study protocol has been **pre-registered, provide DOI in the manuscript**. For clinical trials, provide the trial registration number **OR** cite DOI.	Not Applicable	
Report the **clinical trial registration number** (at ClinicalTrials.gov or equivalent), where applicable.	Not Applicable	

Laboratory protocol	Information included in the manuscript?	In which section is the information available? (Reagents and Tools Table, Materials and Methods, Figures, Data Availability Section)
Provide DOI OR other citation details if **external detailed step-by-step protocols** are available.	Not Applicable	

Experimental study design and statistics	Information included in the manuscript?	In which section is the information available? (Reagents and Tools Table, Materials and Methods, Figures, Data Availability Section)
Include a statement about **sample size** estimate even if no statistical methods were used.	Yes	Materials and Methods
Were any steps taken to minimize the effects of subjective bias when allocating animals/samples to treatment (e.g. **randomization procedure**)? If yes, have they been described?	Not Applicable	
Include a statement about **blinding** even if no blinding was done.	Yes	Materials and Methods
Describe **inclusion/exclusion criteria** if samples or animals were excluded from the analysis. Were the criteria pre-established?<br><br>If sample or data points were omitted from analysis, report if this was due to attrition or intentional exclusion and provide justification.	Not Applicable	
For every figure, are **statistical tests** justified as appropriate? Do the data meet the assumptions of the tests (e.g., normal distribution)? Describe any methods used to assess it. Is there an estimate of variation within each group of data? Is the variance similar between the groups that are being statistically compared?	Yes	Materials and Methods

Sample definition and in-laboratory replication	Information included in the manuscript?	In which section is the information available? (Reagents and Tools Table, Materials and Methods, Figures, Data Availability Section)
In the figure legends: state number of times the experiment was **replicated** in laboratory.	Yes	Figure legends
In the figure legends: define whether data describe **technical or biological replicates**.	Yes	Figure legends

Ethics

Ethics	Information included in the manuscript?	In which section is the information available? (Reagents and Tools Table, Materials and Methods, Figures, Data Availability Section)
Studies involving **human participants**: State details of **authority granting ethics approval** (IRB or equivalent committee(s), provide reference number for approval.	Not Applicable	
Studies involving **human participants**: Include a statement confirming that **informed consent** was obtained from all subjects and that the experiments conformed to the principles set out in the WMA Declaration of Helsinki and the Department of Health and Human Services Belmont Report.	Not Applicable	
Studies involving **human participants:** For publication of **patient photos**, include a statement confirming that consent to publish was obtained.	Not Applicable	
Studies involving experimental **animals**: State details of **authority granting ethics approva**l (IRB or equivalent committee(s), provide reference number for approval. Include a statement of compliance with ethical regulations.	Not Applicable	
Studies involving **specimen and field samples:** State if relevant **permits** obtained, provide details of authority approving study; if none were required, explain why.	Not Applicable	

Dual Use Research of Concern (DURC)	Information included in the manuscript?	In which section is the information available? (Reagents and Tools Table, Materials and Methods, Figures, Data Availability Section)
Could your study fall under dual use research restrictions? Please check biosecurity documents and list of **select agents and toxins** (CDC): https://www.selectagents.gov/sat/list.htm	Not Applicable	
If you used a select agent, is the security level of the lab appropriate and reported in the manuscript?	Not Applicable	
If a study is subject to dual use research of concern regulations, is the name of the **authority granting approval and reference number** for the regulatory approval provided in the manuscript?	Not Applicable	

Reporting

The MDAR framework recommends adoption of discipline-specific guidelines, established and endorsed through community initiatives. Journals have their own policy about requiring specific guidelines and recommendations to complement MDAR.

Adherence to community standards	Information included in the manuscript?	In which section is the information available? (Reagents and Tools Table, Materials and Methods, Figures, Data Availability Section)
State if relevant guidelines or checklists (e.g., **ICMJE, MIBBI, ARRIVE, PRISMA**) have been followed or provided.	Not Applicable	
For **tumor marker prognostic studies**, we recommend that you follow the **REMARK** reporting guidelines (see link list at top right). See author guidelines, under 'Reporting Guidelines'. Please confirm you have followed these guidelines.	Not Applicable	
For **phase II and III randomized controlled trials**, please refer to the **CONSORT** flow diagram (see link list at top right) and submit the CONSORT checklist (see link list at top right) with your submission. See author guidelines, under 'Reporting Guidelines'. Please confirm you have submitted this list.	Not Applicable	

Data Availability

Data availability	Information included in the manuscript?	In which section is the information available? (Reagents and Tools Table, Materials and Methods, Figures, Data Availability Section)
Have **primary datasets** been deposited according to the journal's guidelines (see 'Data Deposition' section) and the respective accession numbers provided in the Data Availability Section?	Not Applicable	
Were **human clinical and genomic datasets** deposited in a public access-controlled repository in accordance to ethical obligations to the patients and to the applicable consent agreement?	Not Applicable	
Are **computational models** that are central and integral to a study available without restrictions in a machine-readable form? Were the relevant accession numbers or links provided?	Not Applicable	
If publicly available data were reused, provide the respective **data citations in the reference list**.	Not Applicable	
