# Supplementary material for: NDP52 acts as a redox sensor in PINK1/Parkin‐mediated mitophagy
Source: EMBO J. 2022 Dec 14;42(5):e111372. doi: 10.15252/embj.2022111372 (PMC9975939; doi:10.15252/embj.2022111372)

Appendix Fig S1

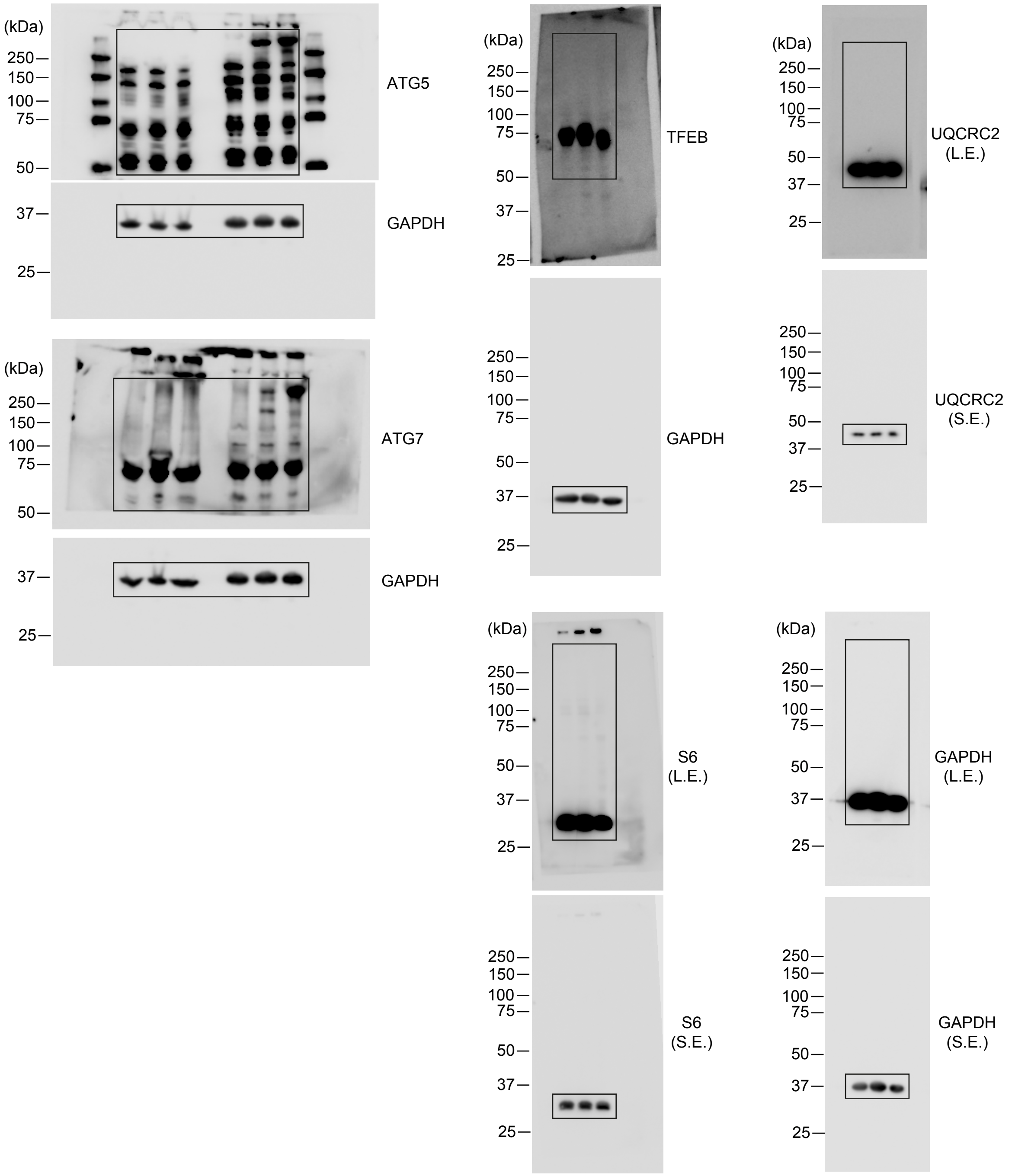

Supplement: Supplementary file 3 — Source Data for Expanded View and Appendix [file EMBJ-42-e111372-s001.zip › EMBOJ-2022-111372_SourceDataForExpandedViewandAppendixv2/EMBOJ-2022-111372_SourceDataForAppendixFigures.zip!/EMBOJ-2022-111372_SourceDataForAppendixFigureS1.pdf]
